# Supplementary material for: Burden of lower respiratory infections and associated risk factors across regions in Ethiopia: a subnational analysis of the Global Burden of Diseases 2019 study
Source: BMJ Open. 2023 Sep 4;13(9):e068498. doi: 10.1136/bmjopen-2022-068498 (PMC10481843; doi:10.1136/bmjopen-2022-068498)
Supplement: Supplementary data [file bmjopen-2022-068498supp001.pdf]

Supplementary material to "The burden of lower respiratory infections and associated risk factors across regions in Ethiopia: A subnational analysis of the Global Burden of Diseases 2019 Study"

This supplementary material provides supplemental figures and tables more detailed results for ""The burden of lower respiratory infections and associated risk factors across regions in Ethiopia: A subnational analysis of the Global Burden of Diseases 2019 Study"

Contents

Lists of Tables and Figures 2
Supplemental Figure 1: Trend in age-standardized incidence rate of LRIs per 100,000 people in Ethiopia, 1990-2019 2
Supplemental Figure 2: Children under 5 years of age incidence rate per 100,000 populations due to LRI in Ethiopia, 1990- 2019 3
Supplemental Figure 3: Trend in LRIs age-standardized mortality rates per 100,000 people in Ethiopia, 1990-2019 4
Supplemental Figure 4: Children younger than 5 years mortality rate per 100,000 populations for lower respiratory infections in Ethiopia, 1990-2019 4
Supplemental Figure 5: Adults older than 70 years mortality rate per 100,000 populations for lower respiratory infections in Ethiopia, 1990-2019 4
Supplemental Figure 6: Children younger than 5 years YLL per 100,000 populations for lower respiratory infections in Ethiopia, 1990-2019 5
Supplemental Figure 8: Attribution of the risk factors to LRIs death rate per 100,000 population in all age groups between 1990 and 2019 for Ethiopia and its regions, both sexes, number of death, 2019. SNNPs: Southern Nations, Nationalities, and Peoples. 6
Supplemental Figure 10: Trends in the risk factors between 1990 and 2019 for all ages for Ethiopia ... 8
Tables........ 9
Supplemental Table 1: Number and percentage changes of episode attributable to LRIs in 1990 and 2019 for Ethiopia, its regions, both sexes 9
Supplemental Table 2: Rate and percentage changes of episodes attributable to LRIs in 1990 and 2019 for Ethiopia and its regions, both sexes 10
Supplemental Table 3: Number and percentage changes of death attributable to LRIs in 1990 and 2019 for Ethiopia and its regions, both sexes. 11
Supplemental Table 4: Age standardized mortality rate, Ethiopia and its regions, by sex, 2019........ 12
Supplemental Table 5: Number and percentage changes of YLL attributable to LRIs in 1990 and 2019 for Ethiopia and its regions, both sexes 12
Supplemental Table 6: Rate and percentage changes of YLL attributable to LRIs in 1990 and 2019 for Ethiopia and its regions, both sexes. 13

Authors contributions 15

Lists of Tables and Figures

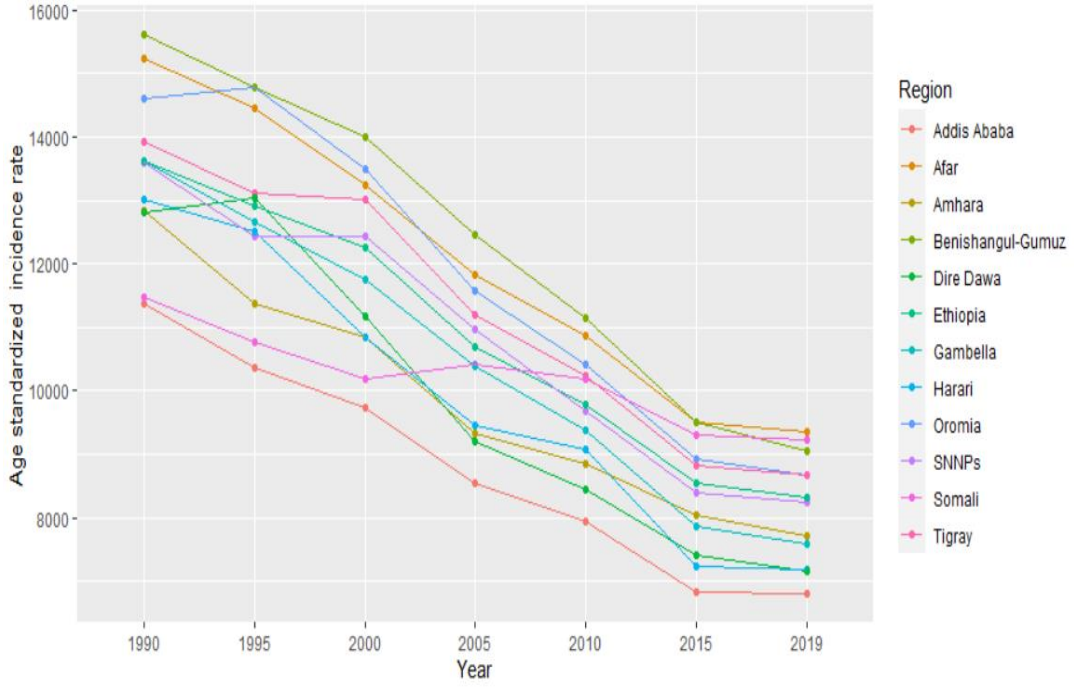

Supplemental Figure 1: Trend in age-standardized incidence rate of LRIs per 100,000 people in Ethiopia, 1990-2019

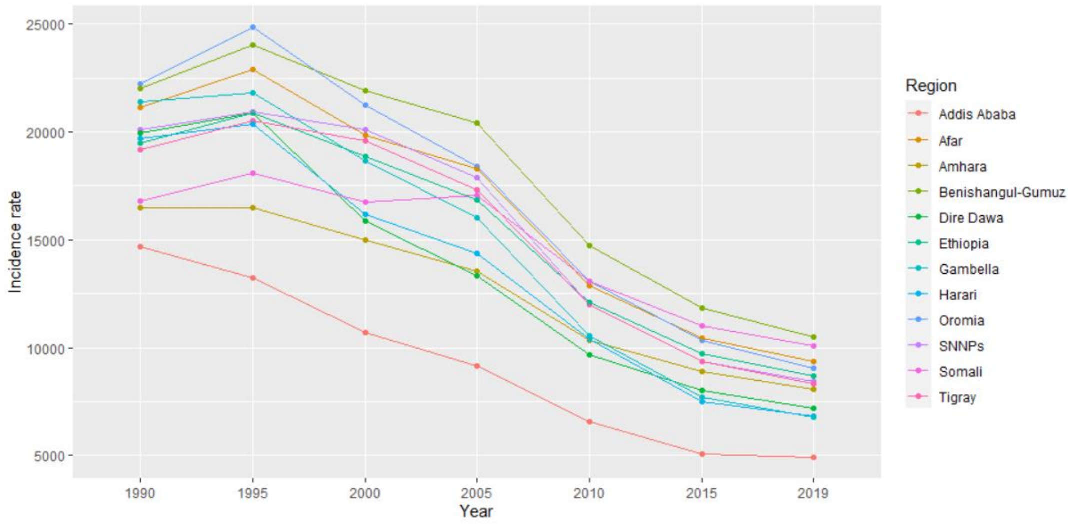

Supplemental Figure 2: Children under 5 years of age incidence rate per 100,000 populations due to LRI in Ethiopia, 1990- 2019

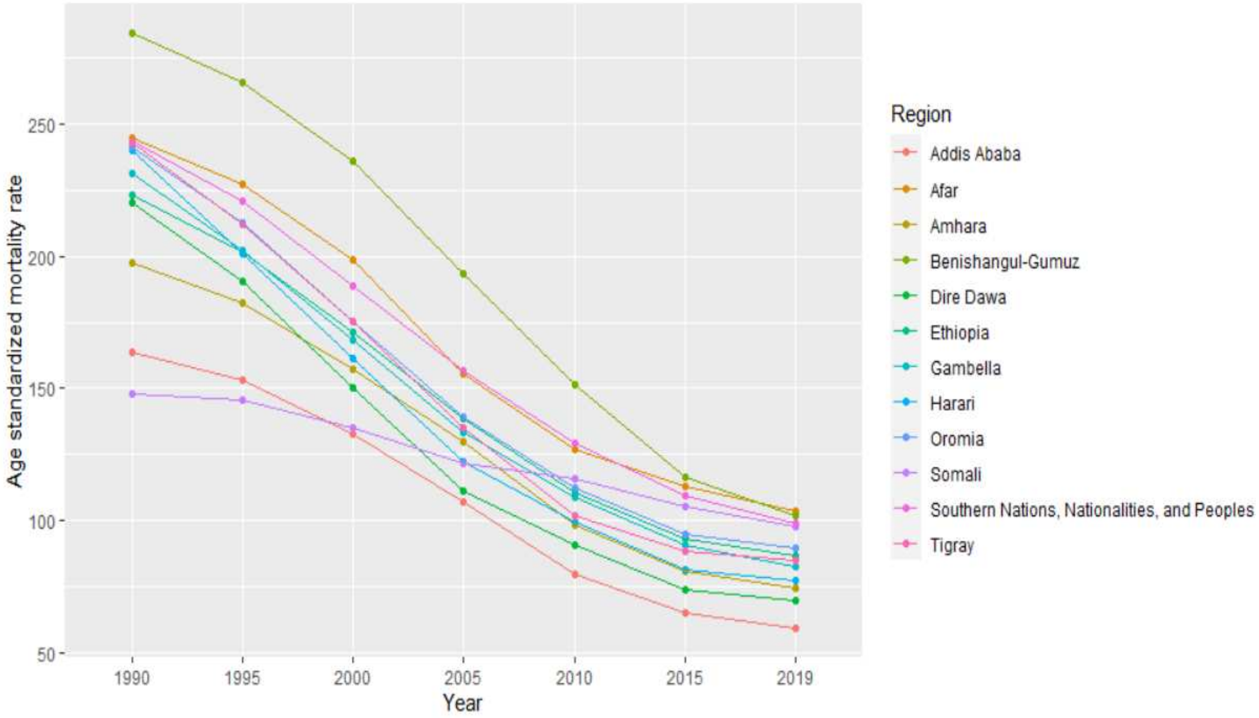

Supplemental Figure 3: Trend in LRIs age-standardized mortality rates per 100,000 people in Ethiopia, 1990-2019

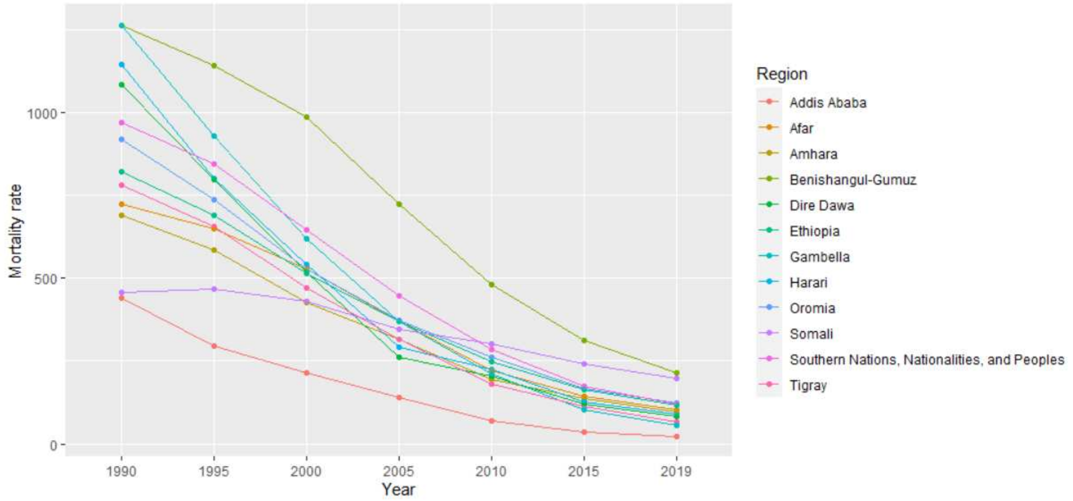

Supplemental Figure 4: Children younger than 5 years mortality rate per 100,000 populations for lower respiratory infections in Ethiopia, 1990-2019

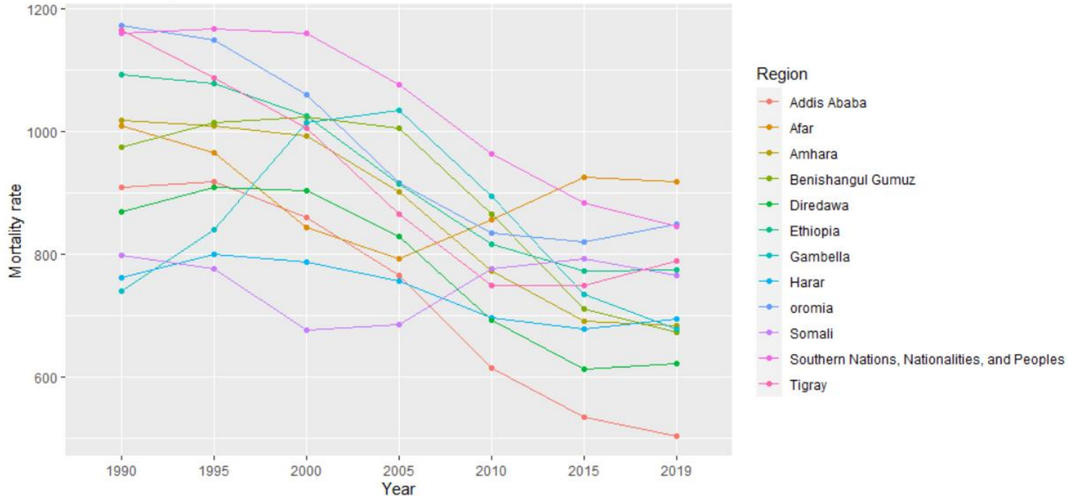

Supplemental Figure 5: Adults older than 70 years mortality rate per 100,000 populations for lower respiratory infections in Ethiopia, 1990-2019

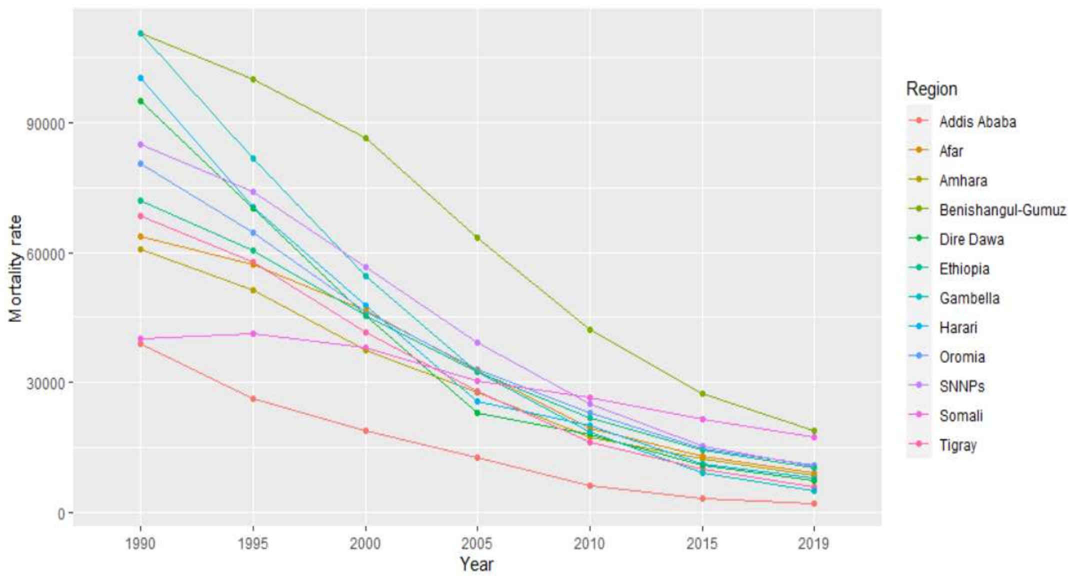

Supplemental Figure 6: Children younger than 5 years YLL per 100,000 populations for lower respiratory infections in Ethiopia, 1990-2019

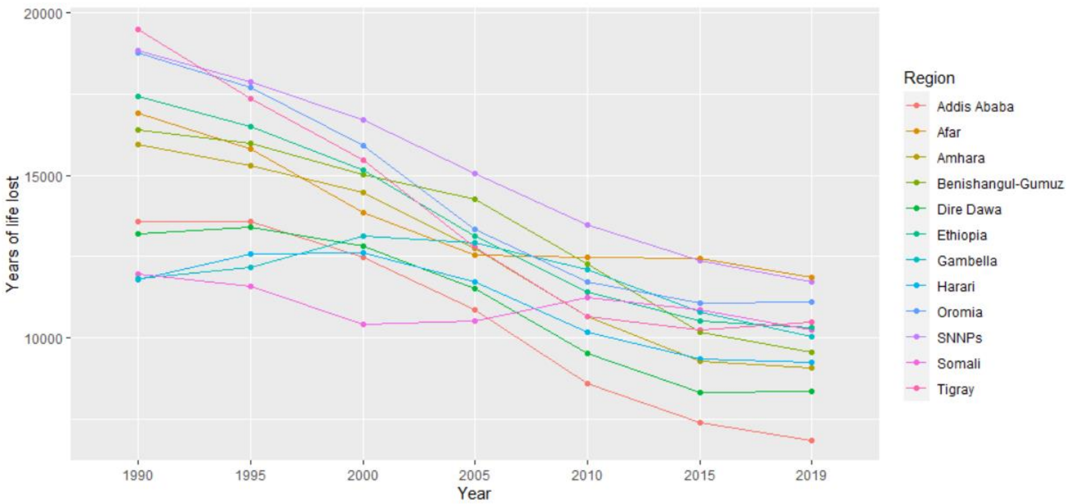

Supplemental Figure 7: Adults older than 70 years YLL per 100,000 populations for lower respiratory infections in Ethiopia, 1990-2019

Location	Alcohol use	Ambient particulate matter pollution	Child stunting	Child underweight	child wasting	High temperature	Household air pollution from solid fuel-	Low birth weight	Low temperature	No access to handwashing facility	Non-exclusive breastfeeding	Secondhand smoke	Short gestation	Smoking
Addis Ababa	1.4	4	0.1	0.1	0.7	0	1.7	0.7	3.7	4.8	0.1	0.4	0.7	1
Oromia	0.6	2.5	2.5	1.9	11.2	0.9	21.6	4.3	2.2	10.3	0.9	0.6	3.5	1.3
Amhara	0.4	2.5	1.8	1.4	6.7	0.9	19.8	3.3	2.4	9.5	0.4	0.6	2.6	0.4
SNNPs	0.8	2.4	2.7	2.2	10.2	1.8	19.9	3.5	1.4	9.6	0.8	0.5	2.9	0.9
Tigray	0.7	3.3	0.8	0.8	4	1.1	17.7	2.8	1.2	9.4	0.3	0.5	2.4	0.4
Harari	1	3.8	0.9	0.7	5.3	0	9.6	2.4	1.1	7.6	0.6	0.6	2.1	3.2
Afar	0.4	2.1	1.8	1.9	8.5	6.3	21	4.5	0.1	9.4	0.9	0.6	3.6	1.4
Somali	0.3	1.6	3.3	3.8	22.2	4.8	31.6	7.2	0.4	13.3	2.5	0.9	5.7	1.8
Benishangul-Gumuz	0.4	3.4	4.5	3.5	20	4.7	27.7	4.9	0.6	13.3	1.3	0.7	4	0.8
Dire Dawa	0.9	3.6	0.7	0.7	5.1	0.4	8.3	2.4	0.5	6.9	0.4	0.5	2	2.1
Gambella	0.8	2.2	0.4	0.4	3.2	2.5	9.3	2.2	0.1	5.4	0.4	0.3	1.9	2.1
Ethiopia	0.6	2.5	2.2	1.8	9.9	1.4	20.5	3.9	1.9	9.9	0.8	0.6	3.2	1

Supplemental Figure 8: Attribution of the risk factors to LRIs death rate per 100,000 population in all age groups between 1990 and 2019 for Ethiopia and its regions, both sexes, number of death, 2019. SNNPs: Southern Nations, Nationalities, and Peoples.

	Ambient particulate matter pollution	child stunting	Child under weight	child wasting	high temperature	Household air pollution from solid fuels	Low birth weight	Low temperature	No access to handwashing facility	Non-exclusive breastfeeding	Secondhand smoke	Short gestation
Addis Ababa	3.63	0.82	0.64	8.35	0.00	1.75	8.86	3.29	4.27	1.32	0.31	7.98
Oromia	6.41	14.75	11.45	67.14	2.34	60.08	25.95	6.05	28.32	5.25	1.66	21.03
Amhara	5.40	13.01	9.84	48.10	2.02	46.86	23.47	5.46	21.99	2.81	1.36	18.88
SNNPs	6.36	16.67	13.77	63.88	5.03	57.68	21.95	4.12	27.49	5.28	1.38	18.31
Tigray	5.03	6.15	5.75	29.88	1.87	29.89	20.59	2.00	15.32	1.91	0.79	17.45
Harari	9.12	7.21	5.80	43.66	0.01	25.51	19.63	2.74	19.30	5.00	1.62	17.18
Afar	4.88	11.42	11.57	52.76	15.77	53.12	28.03	0.26	23.50	5.49	1.33	22.31
Somali	5.40	18.08	20.92	121.83	16.76	110.21	39.76	1.46	46.07	13.99	3.18	31.30
Benishangul-Gumuz	12.63	28.22	22.25	125.72	17.81	105.51	30.75	2.22	50.23	8.05	2.61	24.89
Dire Dawa	9.38	5.50	5.64	42.47	1.11	23.88	19.72	1.42	18.88	3.67	1.50	16.71
Gambella	4.75	3.13	3.08	25.92	6.16	23.51	18.20	0.18	13.02	2.86	0.60	15.31
Ethiopia	6.06	14.39	11.93	64.00	4.28	58.22	25.08	4.73	27.20	5.22	1.58	20.40

Supplemental Figure 9: Attribution of the risk factors to LRIs death rate per 100,000 population in children younger than 5 years between 1990 and 2019 for Ethiopia and its regions, both sexes, number of death, 2019. SNNPs: Southern Nations, Nationalities, and Peoples.

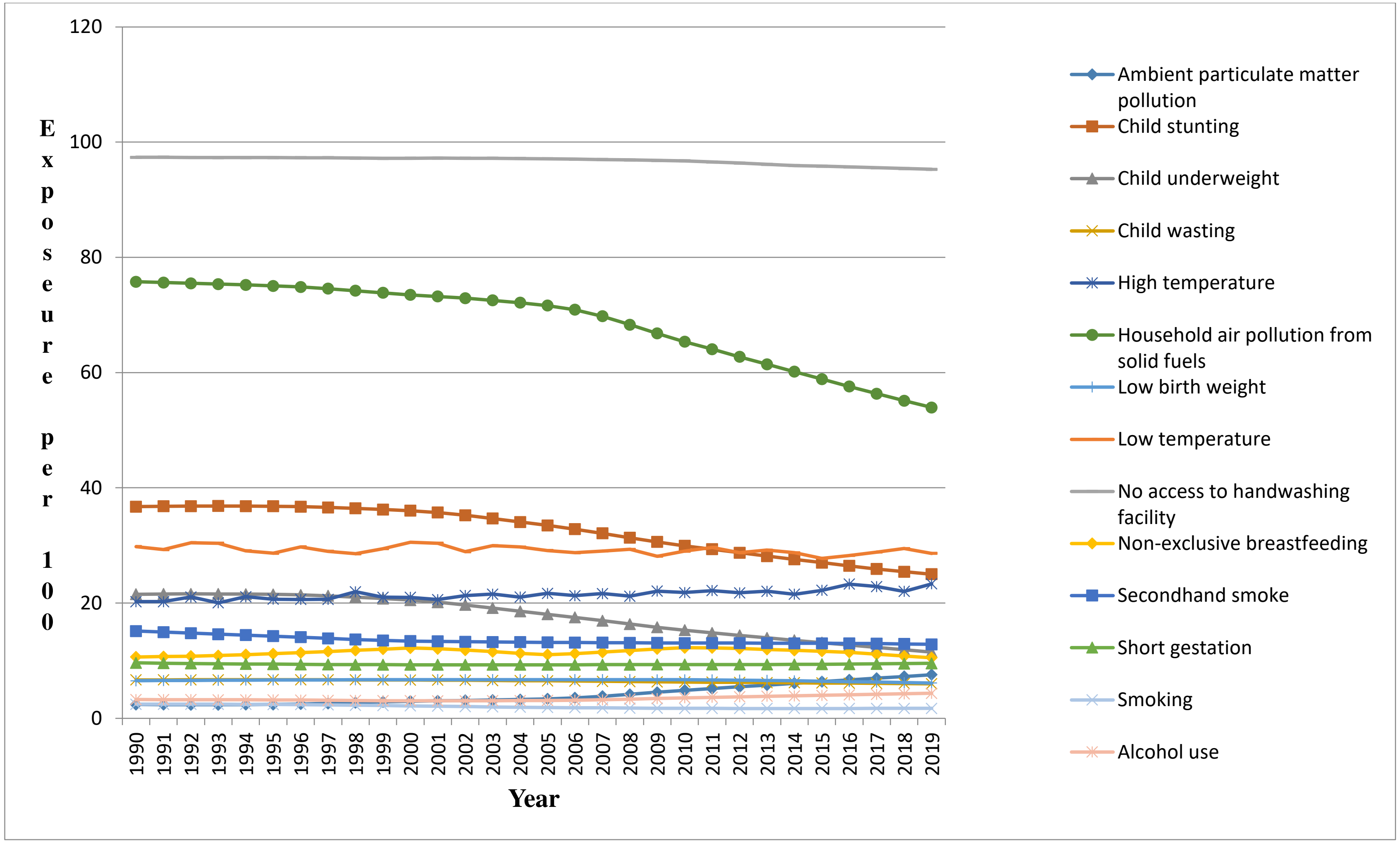

Supplemental Figure 10: Trends in the risk factors between 1990 and 2019 for all ages for Ethiopia

Tables

Supplemental Table 1: Number and percentage changes of episode attributable to LRIs in 1990 and 2019 for Ethiopia, its regions, both sexes

	All ages			Children younger than 5 years			People above 70		
Location	Episode, (95%UI),1990	Episode, (95%UI),2019	Change, %	Episode, (95%UI),1990	Episode, (95%UI),2019	Change, %	Episode, (95%UI),1990	Episode, (95%UI),2019	Change,%
Addis Ababa	194122.9(175403.8-213856.2)	176027.6(162152.3-190557.8)	10	44940.6(35673.3-56859.6)	14522.3(11237-18367.5)	68	8099.5(6891.6-9463.2)	24034.2(20749.5-27702.3)	196**
Oromia	2164243.7(1957719.5-2397957.9)	2597863.9(2372089.2-2859925.6)	20	739239.6(578477-935022.8)	613676.6(480687.2-765626.5)	17	110627.2(95303.7-129579.9)	292179.4(254670.4-342438.5)	164**
Amhara	1508431.4(1368059.8-1671550.1)	1400682.4(1285323-1530939.8)	8	423999.3(334700.8-538426.8)	261397.4(206754.7-327889.7)	39	116332.8(98774.4-137194.5)	198977.5(173809.4-229925.5)	71**
SNNPs	1235554.9(1108317.9-1368520.3)	1330491.5(1217176.7-1452694.1)	7	421825.6(335087.1-526155.2)	307771.3(248326.4-385053.7)	28	68084.5(57450-79640.8)	99723(88168.3-113438)	46**
Tigray	360795.1(328276.3-400714.9)	409829.8(376290.4-445030.2)	13	109047.9(86435.2-137691.1)	69958.2(55210.7-87468.6)	36	20653.3(17469.9-24256.6)	62677.2(53933.1-72927.6)	203**
Harari	15145.9(13618.9-16851.9)	13765.3(12657-15054.7)	10	4693.6(3691.4-5990.1)	2188.4(1723.8-2761.3)	54	307.2(259.6-357)	1614.8(1396-1863.6)	425**
Afar	113506.8(102317-126084.3)	113210.3(102987.9-124578)	1	32991.6(25672.4-42147.6)	26177.2(20744.1-32743.3)	21	2640.2(2212.5-3150.9)	7222.5(6246.8-8559.1)	173**
Somali	300306.3(268988-336331.8)	463220.4(420429.8-516009.4)	54	103526.3(81391.3-131352.8)	126089.6(99611.9-160423.3)	21**	6182.3(5316-7370.4)	30203.2(25942.8-35038.5)	388**
BG	68501.6(61804.9-75736.4)	71675.7(64972.1-79105.8)	4	20970.5(16626.7-26454.5)	18175.6(14339.5-22922.1)	14	3288.9(2784.8-3911.4)	4311.3(3743.8-5113.9)	31**
Dire Dawa	30607.1(27359.8-34252.2)	26221.1(24010.1-28647.8)	15	9547.4(7523-12185)	4387.7(3439.6-5583.9)	55	1274.8(1090.1-1483.5)	2840.1(2469-3289.5)	122**
Gambella	19687.9(17723.9-21945.7)	25685.2(23516.6-28093.2)	30**	6747.7(5338.7-8613.7)	4335.4(3428.4-5400.9)	36	1568.1(1316.6-1825.8)	1489.6(1281.4-1737.3)	6
Ethiopia*	6010904.2(5467801.6-6648644.3)	6628673.6(6108786.2-7230986.3)	10**	1917530.8(1518850.1-2400978.5)	1448680.4(1150089.8-1799704.4)	25	339059.4(293491.9-387514.2)	725273.3(640315.4-837746.3)	**113****

SNNP: Southern Nations, Nationalities, and Peoples; BG: Benishangul Gumuz; *country's estimate; ***percentage increase between 1990 and 2019*

Supplemental Table 2: Rate and percentage changes of episodes attributable to LRIs in 1990 and 2019 for Ethiopia and its regions, both sexes

	**Age standardized**			**Children younger than 5 years**			**People above70**		
**Location**	Episode per 100,000 people (95%UI),1990	Episode per 100,000 people (95%UI),2019	Change,%	Episode per 100,000 people (95%UI),1990	Episode per 100,000 people (95%UI),2019	Change,%	Episode per 100,000 people (95%UI),1990	Episode per 100,000 people (95%UI),2019	Change,%
Addis Ababa	11373(10484.2-12197.4)	6788.1(6285.1-7339.1)	40	14666.9(11642.4-18556.9)	4927.2(3812.5-6231.8)	67	35138(29897.7-41054.2)	30748.4(26546-35441.2)	13
Oromia	14613.1(13525.9-15725.8)	8659.9(8040.1-9411.6)	41	22232(17397.2-28120)	9039.4(7080.5-11277.7)	60	46498.3(40057.6-54464.4)	41564.6(36228.7-48714.4)	11
Amhara	12826.4(11830-13882.8)	7716.9(7162.3-8321.3)	40	16487.1(13014.7-20936.6)	8061.7(6376.4-10112.3)	52	43580(37002.4-51395.2)	35377.7(30902.9-40880.2)	19
SNNPs	13591.9(12538.6-14646.8)	8235.5(7670.5-8846.4)	39	20106.4(15972-25079.3)	8427.1(6799.4-10543.2)	59	44628.1(37657.5-52203.1)	36616.1(32373.5-41652)	18
Tigray	13927.2(12858.2-15100.7)	8663.8(8034-9382.3)	38	19185.4(15207-24224.8)	8342.1(6583.5-10430.1)	57	43748.4(37005.1-51381)	40859(35158.8-47541.2)	7
Harari	13014.6(11941-14066.7)	7190.5(6684.1-7718.6)	45	19672.6(15471.9-25106.9)	6821.6(5373.5-8607.3)	66	33916.7(28665.1-39416.7)	32797.4(28354-37850.7)	4
Afar	15245.7(14050.8-16525.6)	9350.1(8648.8-10157.4)	39	21151.6(16459.1-27021.7)	9365.5(7421.7-11714.7)	56	39849(33393.8-47557.2)	44368.7(38375.2-52580.1)	11**
Somali	11482.7(10638.1-12368.7)	9220(8515.8-10046.3)	20	16792.9(13202.4-21306.6)	10062.7(7949.6-12802.8)	41	32748.3(28159.7-39042.1)	41031.2(35243.4-47600)	25**
BG	15628.1(14481-16809.3)	9054.6(8394.2-9766)	42	22041.1(17475.5-27805)	10481.8(8269.5-13219)	53	42911.6(36334.5-51032.5)	37592.7(32643.9-44591.2)	13
Dire Dawa	12806.6(11807.9-13871.4)	7148.8(6634.6-7750.7)	44	19968.2(15734.3-25484.7)	7191.5(5637.6-9152.1)	64	38333(32779.4-44607)	32033.6(27847.4-37101.4)	17
Gambella	13623.1(12637.4-14572.4)	7575.8(7002.3-8128.2)	44	21419.8(16947.2-27343.2)	6791.1(5370.4-8460.1)	69	40197.8(33751.8-46803.7)	32657.1(28093.9-38087.2)	19
Ethiopia*	13619.7(12640.8-14588.6)	8313.7(7757.6-8918)	39	19486.4(15434.9-24399.3)	8685.3(6895.1-10789.8)	56	44092.2(38166.5-50393.4)	38394.4(33896.9-44348.5)	13

SNNP: Southern Nations, Nationalities, and Peoples; BG: Benishangul Gumuz; *country's estimate*; **percentage increase between 2019 and 1990*

Supplemental Table 3: Number and percentage changes of death attributable to LRIs in 1990 and 2019 for Ethiopia and its regions, both sexes.

	**All age**			**Children younger than 5**			**People above 70 years**		
**Location**	death, (95%UI),1990	death, (95%UI),2019	Change,%	death, (95%UI), 1990	Death, (95%UI),2019	Change, %	Death , (95%UI),1990	Death, (95%UI),2019	Change,%
Addis Ababa	2159.3(1731.9-2690.7)	918.8(767.4-1116.9)	58	1353(981.9-1846.7)	69.1(41.7-107.4)	95	209.5(154.3-291.5)	393.6(320.9-495.3)	87**
Oromia	40004.4(29432.4-51942.2)	18206.1(15193.3-21745.4)	55	30614.3(20932.5-42033.5)	8306.2(5763.5-11611.8)	73	2793.5(2005.1-3673)	5971.6(4831.7-7093.4)	113**
Amhara	25449.3(20869.5-30724.8)	9525.5(7530.5-11872.2)	63	17780.1(13674.3-22720.1)	3079.5(1805.3-4737.3)	83	2717.2(2016.4-3535.8)	3839.9(3022.6-4825.6)	41**
SNNPs	26044.7(20108-33017)	9494.7(7713.5-11649)	-64	20390.6(14627.6-26712.9)	4326.6(2891.3-6157.2)	79	1771.7(1254.5-2417.6)	2302.6(1867.3-2772.8)	29**
Tigray	6463.8(5272.1-7762.9)	2551.4(2090.3-3028.6)	61	4436(3373.5-5736)	564.2(364.9-825)	88	550.8(395.7-749.1)	1209.7(978.5-1467.8)	119**
Harari	336(233.3-448.8)	93.1(73.8-116)	73	273.5(174.1-382.7)	28.8(16.4-44.3)	90	6.9(3.7-11)	34.1(27.1-41.8)	395**
Afar	1717.5(1300.7-2243.4)	706.2(567.7-869.6)	-59	1128.9(751.1-1596.7)	284.2(182.5-426)	75	66.9(43.5-102.1)	149.3(117.3-186.6)	123**
Somali	3603.8(2620-4713.6)	3907.5(3007.4-4959.4)	#N/A	2811.1(1929.6-3874.8)	2472.2(1695.2-3506.8)	13	150.8(98-222.3)	563.1(433.4-715.8)	273**
BG	1549.2(1131.1-2097)	619(470.7-803)	61	1204.8(805.6-1703.9)	373(245.3-539.3)	70	74.7(50.3-105.7)	77(61.1-97.3)	3**
Dire Dawa	632.3(445.5-830.9)	154.6(120.6-193.8)	76	518.4(329.5-718.7)	51(27.3-82.9)	91	28.8(20.1-40)	55(44-67.9)	90**
Gambella	475.9(328.5-635.7)	123.2(98.2-151.9)	75	398.6(253.7-555.7)	36.4(20.7-57.2)	91	28.8(18.6-41.9)	30.9(24.6-38.1)	7**
Ethiopia*	108436.6(87669-132758.3)	46300.7(39515.8-54642.2)	58	80909.9(62491.6-103448.8)	19591.8(14018.4-26899)	76	8400.1(6490.3-10700.9)	14627.6(12393.7-16892.8)	74**

SNNP: Southern Nations, Nationalities, and Peoples; BG: Benishangul Gumuz; *country's estimate; ***percentage increase between 2019 and 1990*

Supplemental Table 4: Age standardized mortality rate, Ethiopia and its regions, by sex, 2019.

Age standardized mortality rate per 100,000 people, 2019		
**Location**	Female	Male
Addis Ababa	47(35.5-62.5)	73.6(55-98.3)
Oromia	77.3(62-92.4)	100.3(79.9-123.5)
Amhara	57.9(44.1-73.7)	92.5(70.5-122.1)
SNNPs	79.2(63.5-95.2)	118.8(94.3-146.3)
Tigray	73.2(55.9-93.5)	96.9(74.8-122.3)
Harari	63(49-79.9)	95.7(72.5-123.4)
Afar	112.4(87.3-142.3)	98.7(76.9-127.3)
Somali	88.3(69-111.4)	101.7(77.2-130.7)
Benishangul-Gumuz	109.2(82.6-138.8)	95.8(74.6-124.1)
Dire Dawa	56.2(42.5-71.3)	85.4(67.3-109.5)
Gambella	47.9(34.6-60.1)	120.2(95.6-149.5)
Ethiopia	71.8(60.2-82.9)	100.6(84-121.4)

SNNP: Southern Nations, Nationalities, and Peoples

Supplemental Table 5: Number and percentage changes of YLL attributable to LRIs in 1990 and 2019 for Ethiopia and its regions, both sexes

	**all age**			**Children younger than 5 years**			**People above 70**		
	YLL, (95%UI),1990	YLL, (95%UI),2019	Change,%	YLL, (95%UI),1990	YLL, (95%UI),2019	Change,%	YLL, (95%UI),1990	YLL, (95%UI),2019	Change,%
Addis Ababa	149543.9(115542.6-195437)	29703.1(23833.4-36855.7)	81	118961.7(86499.1-162678.7)	6098.2(3693.1-9483.3)	95	3131.8(2301.7-4374.6)	5359.8(4334.8-6816.8)	71**
Oromia	3019031.9(2144916.2-4057496.3)	981808.4(748459.2-1278135)	68	2680397.2(1832260.4-3680767.1)	729313.8(506226.4-1021016.9)	73	44619.5(31636-59105.6)	78166.1(62837.5-93527.9)	75**
Amhara	1813522.3(1445106.4-2246142.2)	428277.8(309674.7-583000.2)	77	1560078.3(1201305.4-1993362.8)	270909.2(158846-416496.1)	83	42578.2(31294.7-56216.9)	50955.1(39726.1-64844.6)	19**
SNNPs	1993643.3(1489598.4-2577052.3)	543135.8(405565.6-711397.4)	73	1785675.1(1284021-2339272.5)	380166.4(254486.8-541224.9)	79	28746.2(20086.2-39789.1)	31964.4(25673.6-38778.5)	11**
Tigray	455613.4(364406.1-569702.1)	96429.9(74139.8-122350.5)	79	389399.3(296618.1-504230.2)	49712.6(32198.3-72764.9)	88	9207.1(6568.7-12629.1)	16086.6(12946.2-19581.8)	74**
Harari	26755.3(17866.5-36380.3)	4273.6(2999.1-5798.4)	85	23959.3(15245.4-33513.8)	2541.6(1449.6-3887.3)	90	106.7(57.6-175.1)	455.9(361.3-558.3)	327**

Afar	124052(89386.4-166880.5)	39507.6(29535.5-52178.5)	69	99155.3(65972.9-140018.8)	24998.1(16028.5-37426.3)	75	1121.4(723.7-1722.7)	1931.7(1521.3-2395.6)	72**
Somali	281532.2(201275.3-374880.8)	266379.4(193669.7-357880.7)	6	247138.5(169803.8-339740.3)	216954(148995.2-307369.5)	13	2256.6(1434.6-3376.4)	7542.1(5740-9680.2)	234**
BG	119211.2(83517.3-165095.7)	42245.6(30388.8-57591.8)	-65	105463.5(70756-148983.1)	32718.1(21576.4-47307.2)	69	1256.7(836.7-1780.3)	1094.3(857.5-1389.1)	13
Dire Dawa	50024.5(33464.5-67580.6)	7267.7(5038-10264.7)	86	45377.4(28907.2-62914.9)	4497.4(2411.8-7298.3)	91	439.2(306.6-609.5)	742.1(586.6-922.8)	68**
Gambella	37680.8(24783.6-51516.1)	6064.4(4389.3-7853.6)	84	34899.5(22233.1-48609.4)	3212.2(1830.4-5034.1)	91	460.7(299.3-663.6)	457.5(352.6-577.6)	1
Ethiopia*	8070611.3(6356905.2-10091563.7)	2445093.7(1934420.8-3119838.6)	70	7090505.4(5482895.7-9059421.2)	1721122.3(1231032.1-2362958.7)	76	133924.6(102756.8-170932.9)	194756.2(165462-225502.1)	45**

SNNP: Southern Nations, Nationalities, and Peoples; BG: Benishangul Gumuz; *country's estimate; ***percentage increase between 2019 and 1990*

Supplemental Table 6: Rate and percentage changes of YLL attributable to LRIs in 1990 and 2019 for Ethiopia and its regions, both sexes.

	**all age**			**Children younger than 5 years**			**People above 70**		
**Location**	YLL per 100,000 people,1990	YLL per 100,000 people, 2019	Change,%	YLL per 100,000 people,1990	YLL per 100,000 people, 2019	Change,%	YLL per 100,000 people,1990	YLL per 100,000 people, 2019	Change,%
Addis Ababa	6362.2(5161.9-7847.9)	1285.6(1065-1561.8)	80	38824.7(28230.1-53092.4)	6857.1(5545.8-8721.2)	95	13587(9985.4-18978.3)	6857.1(5545.8-8721.2)	50
Oromia	11217.6(8517-14318)	2433.8(2042.2-2879.5)	78	80610.7(55103.7-110696)	11119.7(8939.1-13305)	87	18754.2(13297.1-24843)	11119.7(8939.1-13305)	41
Amhara	8690.4(7146.3-10453.8)	2016.4(1551.4-2541.8)	77	60663.3(46712.5-77511.5)	9059.7(7063.2-11529.2)	87	15950.4(11723.4-21059.7)	9059.7(7063.2-11529.2)	44
SNNPs	11633(9119.4-14477.9)	2698.1(2243.5-3250.2)	77	85114.7(61203.2-111502.1)	11736.6(9426.7-14238.6)	88	18842.6(13166.1-26081)	11736.6(9426.7-14238.6)	38
Tigray	10346.4(8522.9-12340.3)	1977.1(1593.9-2395)	81	68509.3(52185.7-88712.2)	10486.8(8439.5-12765.3)	92	19502.8(13914.1-26751.2)	10486.8(8439.5-12765.3)	47
Harari	12826.5(9336.5-16985.4)	2060.4(1595.6-2623.1)	84	100421.7(63898.7-140467.8)	9260.4(7338.7-11340.3)	93	11782.6(6360.4-19341.1)	9260.4(7338.7-11340.3)	22
Afar	10993.5(8520.8-14193.6)	2824.6(2323-3414)	74	63570.5(42296.6-89769)	11867.1(9346-14716.9)	86	16925.5(10923.7-26001.7)	11867.1(9346-14716.9)	30
Somali	6286.9(4860.5-7970)	3236.4(2537.4-4006)	49	40088.1(27543.7-55109)	10246(7797.8-13150.6)	57	11953.6(7599.2-17885.5)	10246(7797.8-13150.6)	15
BG	14965.1(11197.4-19998.6)	3571.1(2772.4-4510.7)	76	110847.2(74368-156588.4)	9541.9(7476.9-12112.3)	83	16397.3(10917-23228.5)	9541.9(7476.9-12112.3)	42

Dire Dawa	11749.7(8605.4-15249.5)	1832.8(1407.3-2363.1)	84	94905.3(60458.5-131584.5)	8371(6616.3-10408.2)	93	13207.9(9219.6-18328.2)	8371(6616.3-10408.2)	37
Gambella	13175.8(9291-17411)	1937.4(1567.6-2344.6)	85	110783.8(70576-154304.1)	10030.9(7730.2-12664.9)	96	11811.4(7673.5-17012.5)	10030.9(7730.2-12664.9)	16
Ethiopia*	10189.1(8347.5-12201.8)	2404.5(2059.4-2833.3)	76	72055.4(55718.5-92064)	10309.9(8759.2-11937.6)	86	17415.9(13362.7-22228.5)	10309.9(8759.2-11937.6)	41

SNNP: Southern Nations, Nationalities, and Peoples; BG: Benishangul Gumuz; *country's estimate*; **percentage increase between 2019 and 1990*

This table indicate the rates of the years of life lost due to lower respiratory infection per 100,000 population for the year 1990 and 2019 and the percentage changes between the two years for three age categories (for all age groups, children younger than 5 years and people above 70 years).

Authors contributions

Providing data or critical feedback on data sources

Semagn Mekonnen Abate, Mesafint Molla Adane, Addis Aklilu, Dejene Tsegaye Alem, Mulusew A Asemahagn, Hunegnaw Abebe, Melaku Ashagrie Belete, Tekleberhan Hailemariam, Tezera Moshago Berheto, Belay Boda Abule Bodicha, Daniel Baza Gargamo, Alemayehu Hailu, Awoke Misganaw, Mohsen Naghavi, Negussie Boti Sidamo, Yonatan Solomon, Shambel Wedajo, Melat Weldemariam, Amanuel Yigezu, Fentabil Getnet, and Yazachew Yismaw.

Developing methods or computational machinery

Semagn Mekonnen Abate, Tezera Moshago Berheto, Alemayehu Hailu, Mohsen Naghavi, Negussie Boti Sidamo, and Amanuel Yigezu.

Providing critical feedback on methods or results

Semagn Mekonnen Abate, Mesafint Molla Adane, Gizachew Taddesse Akalu, Addis Aklilu, Dejene Tsegaye Alem, Zeleke Gebru, Mulusew Andualem Asemahagn, Daniel Atlaw, Tewachew Awoke, Hunegnaw Abebe, Melaku Ashagrie Belete, Tekleberhan Hailemariam, Tezera Moshago Berheto, Alemeshet Yirga, Setognal Birara Aychiluhm, Belay Boda Abule Bodicha, Chuchu Churko, Feleke Mekonnen Demeke, Abebaw Alemayehu Desta, Lankamo Ena, Tahir Eyayu, Zinabu Fentaw, Daniel Baza Gargamo, Mesfin Damtew Gebrehiwot, Mathewos Alemu Gebremichael, Melaku Getachew, Ababi Zergaw, Alemayehu Hailu, Getahun Molla, Awoke Misganaw, Mohsen Naghavi, Biniyam Sahiledengle, Bereket Beyene, Migbar Sibhat, Negussie Boti Sidamo, Damtew Damtew Solomon, Yonatan Solomon, Birhanu Wagaye, Shambel Wedajo, Melat Weldemariam, Amanuel Yigezu, Fentabil Getnet, and Yazachew Yismaw.

Drafting the work or revising is critically for important intellectual content

Semagn Mekonnen Abate, Gizachew Taddesse Akalu, Mulusew A Asemahagn, Daniel Atlaw, Niguss Cherie Bekele, Melaku Ashagrie Belete, Tezera Moshago Berheto, Setognal Birara Aychiluhm, Belay Boda Abule Bodicha, Chuchu Churko, Tahir Eyayu, Zinabu Fentaw, Daniel Baza Gargamo, Melaku Getachew, Ababi Zergaw, Firehiwot Abebe Gobena, Muluken Argaw Haile, Alemayehu Hailu, Solomon Tessema Memirie, Awoke Misganaw, Mohsen Naghavi, Biniyam Sahiledengle, Bereket Beyene, Negussie Boti Sidamo, Yonatan Solomon, Dereje Mengistu Tolosa, Birhanu Wagaye, Ally Walker, Amanuel Yigezu, and Fentabil Getnet.

Managing the estimation or publications process

Semagn Mekonnen Abate, Awoke Misganaw, Mohsen Naghavi, Negussie Boti Sidamo, and Amanuel Yigezu.
