# Supplementary material for: Burden of lower respiratory infections and associated risk factors across regions in Ethiopia: a subnational analysis of the Global Burden of Diseases 2019 study
Source: BMJ Open. 2023 Sep 4;13(9):e068498. doi: 10.1136/bmjopen-2022-068498 (PMC10481843; doi:10.1136/bmjopen-2022-068498)
Supplement: Author's manuscript [file bmjopen-2022-068498.draft_revisions.pdf]

BMJ Open is committed to open peer review. As part of this commitment we make the peer review history of every article we publish publicly available.

When an article is published we post the peer reviewers' comments and the authors' responses online. We also post the versions of the paper that were used during peer review. These are the versions that the peer review comments apply to.

The versions of the paper that follow are the versions that were submitted during the peer review process. They are not the versions of record or the final published versions. They should not be cited or distributed as the published version of this manuscript.

BMJ Open is an open access journal and the full, final, typeset and author-corrected version of record of the manuscript is available on our site with no access controls, subscription charges or pay-per-view fees (http://bmjopen.bmj.com).

If you have any questions on BMJ Open's open peer review process please email info.bmjopen@bmj.com

BMJ Open

# The Burden of Lower Respiratory Infections and the Risk Factors across Regions in Ethiopia: Global Burden of Diseases Study 1990-2019

| | |
|---|---|
| Journal: | *BMJ Open* |
| Manuscript ID | bmjopen-2022-068498 |
| Article Type: | Original research |
| Date Submitted by the Author: | 13-Oct-2022 |
| Complete List of Authors: | Yigezu, Amanuel; Ethiopian Public Health Institute<br>Misganaw, Awoke; University of Washington, Department of Health Metrics Sciences; Ethiopian Public Health Association,<br>Getnet, Fentabil; Jigjiga University; Ethiopian Public Health Institute<br>Berheto, Tezera; Ethiopian Public Health Institute<br>Walker, Ally ; University of Washington, Department of Health Metrics Sciences<br>Zergaw, Ababi; Ethiopian Public Health Institute; Addis Ababa University College of Health Sciences, Department of Health Systems and Policy<br>Gobena, Firehiwot ; Ethiopian Health Insurance Service<br>Haile, Muluken ; Ethiopian Health Insurance Service<br>Hailu, Alemayehu; University of Bergen, Department of Global Public Health and Primary Care Medicine<br>Memirie, Solomon Tessema; Addis Ababa University, Addis Center for Ethics and Priority Setting; Harvard University, Harvard T.H. Chan School of Public Health<br>Tolosa, Dereje ; Ethiopian Health Insurance Service<br>Abate, Semagn Mekonnen; Dilla University College of Health Sciences, Department of Anesthesiology<br>Molla Adane, Mesafint; Bahir Dar University College of Medical and Health Sciences<br>Akalu, Gizachew ; St Paul's Hospital Millennium Medical College; Addis Ababa University College of Health Sciences<br>Aklilu, Addis ; Arba Minch University<br>Alem, Dejene; Debre Markos University College of Health Science, Adult health nursing;<br>Gebru, Zeleke; Arba Minch University, public health; Arba minch university<br>Asemahagn, Mulusew; Bahir Dar University College of Medical and Health Sciences, School of Public Health;<br>Atlaw, Daniel; Madda Walabu University, ;<br>Awoke, Tewachew ; Bahir Dar University<br>Abebe, Hunegnaw ; Wollo University, public health<br>Belete, Melaku; Wollo University<br>Hailemariam, Tekleberhan; Mekelle University<br>Yirga, Alemeshet; Bahir Dar University, Department of pharmacy<br>Birara, Setognal; Samara University, Public Health<br>Bodicha, Belay ; Arba Minch University<br>Churko, Chuchu ; Arba Minch University, Public health |

Demeke, Feleke ; Bahir Dar University College of Medical and Health Sciences
Desta, Abebaw ; University of Gondar
Ena, Lankamo; Arba Minch University,
Eyayu, Tahir; Debre Tabor University, Department of Medical Laboratory Sciences
Fentaw, Zinabu; Wollo University, Department of Epidemiology and Biostatistics; Wollo University, Epidemiology and Biostatistics
Gargamo, Daniel ; Wolaita Sodo University
Gebrehiwot, Mesfin ; Wollo University
Gebremichael, Mathewos ; Arba Minch University
Getachew, Melaku ; Haramaya University College of Health and Medical Sciences
Molla, Getahun ; University of Gondar, University of Gondar Hospital
Sahiledengle, Biniyam; Madda Walabu University, Public Health
Beyene, Bereket; Arba Minch University
Sibhat, Migbar; Dilla University College of Health Sciences, Nursing;
Sidamo, Negussie ; Arba Minch University, Public Health
Solomon, Damtew ; Madda Walabu University
Solomon, Yonatan ; Dire Dawa University
Wagaye, Birhanu; Wollo University; Ethiopian Public Health Institute
Wedajo, Shambel; Wollo University,
Weldemariam, Melat; Arba Minch University, Department of Medical Laboratory Sciences
Yismaw, Yazachew ; Bahir Dar University
Naghavi, Moshen; University of Washington School of Public Health,

Keywords: Epidemiology < INFECTIOUS DISEASES, Respiratory infections < THORACIC MEDICINE, Health policy < HEALTH SERVICES ADMINISTRATION & MANAGEMENT

SCHOLARONE™
Manuscripts

*I, the Submitting Author has the right to grant and does grant on behalf of all authors of the Work (as defined in the below author licence), an exclusive licence and/or a non-exclusive licence for contributions from authors who are: i) UK Crown employees; ii) where BMJ has agreed a CC-BY licence shall apply, and/or iii) in accordance with the terms applicable for US Federal Government officers or employees acting as part of their official duties; on a worldwide, perpetual, irrevocable, royalty-free basis to BMJ Publishing Group Ltd ("BMJ") its licensees and where the relevant Journal is co-owned by BMJ to the co-owners of the Journal, to publish the Work in this journal and any other BMJ products and to exploit all rights, as set out in our licence.*

*The Submitting Author accepts and understands that any supply made under these terms is made by BMJ to the Submitting Author unless you are acting as an employee on behalf of your employer or a postgraduate student of an affiliated institution which is paying any applicable article publishing charge ("APC") for Open Access articles. Where the Submitting Author wishes to make the Work available on an Open Access basis (and intends to pay the relevant APC), the terms of reuse of such Open Access shall be governed by a Creative Commons licence – details of these licences and which Creative Commons licence will apply to this Work are set out in our licence referred to above.*

*Other than as permitted in any relevant BMJ Author's Self Archiving Policies, I confirm this Work has not been accepted for publication elsewhere, is not being considered for publication elsewhere and does not duplicate material already published. I confirm all authors consent to publication of this Work and authorise the granting of this licence.*

For peer review only

# The Burden of Lower Respiratory Infections and the Risk Factors across Regions in Ethiopia: Global Burden of Diseases Study 1990-2019

Ethiopia Subnational-Level Lower Respiratory Infections Burden collaborators

Amanuel Yigezu*,[1] Awoke Misganaw*,[2,1] Fentabil Getnet,[1,3] Tezera Moshago Berheto,[4] Ally Walker,[5] Ababi Zergaw,[6,1] Firehiwot Abebe Gobena,[7] Muluken Argaw Haile,[8] Alemayehu Hailu,[9] Solomon Tessema Memirie,[10] Dereje Mengistu Tolosa,[11] Semagn Mekonnen Abate,[12] Mesafint Molla Adane,[13] Gizachew Taddesse Akalu,[14,15] Addis Aklilu,[16] Dejene Tsegaye Alem,[17] Zeleke Gebru,[18] Mulusew Andualem Asemahagn,[19] Daniel Atlaw,[20] Tewachew Awoke,[21] Hunegnaw Abebe[22], Melaku Ashagrie Belete,[23] Tekleberhan Hailemariam,[24] Alemeshet Yirga,[25] Setognal Birara Aychiluhm,[26] Belay Boda Abule Bodicha,[27] Chuchu Churko,[18] Feleke Mekonnen Demeke,[21] Abebaw Alemayehu Desta,[28] Lankamo Ena,[29] Tahir Eyayu,[30] Zinabu Fentaw,[31] Daniel Baza Gargamo,[32] Mesfin Damtew Gebrehiwot,[33] Mathewos Alemu Gebremichael,[34] Melaku Getachew,[35] Getahun Molla,[36] Biniyam Sahiledengle,[37] Bereket Beyene,[38] Migbar Sibhat,[39] Negussie Boti Sidamo,[18] Damtew Solomon,[40] Yonatan Solomon,[41] Birhanu Wagaye,[42,43] Shambel Wedajo,[22] Melat Weldemariam,[16] Yazachew Yismaw,[44,45] Mohsen Naghavi.[5,2]

*Joint first authors

## Affiliations

[1]National Data Management Center for Health (NDMC), Ethiopian Public Health Institute, Addis Ababa, Ethiopia; [2]Department of Health Metrics Sciences, School of Medicine, University of Washington, Seattle, WA, USA; [3]Department of Epidemiology, Jigjiga University, Jigjiga, Ethiopia; [4]HIV and TB Research Directorate, Ethiopian Public Health Institute, Addis Ababa, Ethiopia; [5]Institute for Health Metrics and Evaluation, University of Washington, Seattle, WA, USA; [6]Department of Health Systems and Policy, Addis Ababa University, Addis Ababa, Ethiopia; [7] Ethiopian Health Insurance Service, Addis Ababa, Ethiopia; [8]Program Section, Ethiopian Health Insurance Service, Addis Ababa, Ethiopia; [9]Department of Global Public Health and Primary Care, University of Bergen, Bergen, Norway; [10]Addis Center for Ethics and Priority Setting, Addis Ababa University, Addis Ababa, Ethiopia; [11]Ethiopian Health Insurance Service, Ministry of Health, Addis Ababa, Ethiopia; [12]Department of Anesthesiology, Dilla University, Addis Ababa, Ethiopia; [13]College of Medicine and Health Sciences, Bahir Dar University, Bahir Dar, Ethiopia; [14]Department of Microbiology, Immunology and Parasitology, St. Paul's Hospital Millennium Medical College, Addis Ababa, Ethiopia; [15]Department of Microbial, Cellular and Molecular Biology, Addis Ababa University, Addis Ababa, Ethiopia; [16]Department of Medical Laboratory Sciences, Arba Minch University, Arba Minch, Ethiopia; [17]Department of Nursing, Debre Markos University, Debremarkos, Ethiopia; [18]School of Public Health, Arba Minch University, Arba Minch, Ethiopia; [19]School of Public Health, Bahir Dar University, Bahir Dar, Ethiopia; [20]Department of Biomedical Science, Madda Walabu University, Bale Robe, Ethiopia; [21]Department of Medical Laboratory Sciences, Bahir Dar University, Bahir Dar, Ethiopia; [22]Department of Public Health, Wollo University, Dessie, Ethiopia; [23]Department of Medical Laboratory Sciences, Wollo University, Dessie, Ethiopia; [24]Department of Medical Physiology,



For peer review only - http://bmjopen.bmj.com/site/about/guidelines.xhtml

Mekelle University, Mekelle, Ethiopia; [25]School of Health Science, Bahir Dar University, Bahir Dar, Ethiopia; [26]Department of Public Health, Samara University, Samara, Ethiopia; [27]Department of Biomedical Sciences , Arba Minch University, Arba Minch, Ethiopia; [28]Department of Surgical Nursing, University of Gondar, Gondar, Ethiopia; [29]Department of Comprehensive Nursing, Arba Minch University, Arba Minch, Ethiopia; [30]Department of Medical Laboratory Sciences, Debre Tabor University, Debre Tabor, Ethiopia; [31]Department of Epidemiology and Biostatistics, Wollo University, Dessie, Ethiopia; [32]Department of Pediatrics and Neonatal Nursing, Wolaita Sodo University, Wolaita Sodo, Ethiopia; [33]Department of Environmental Health, Wollo University, Dessie, Ethiopia; [34]Department of Epidemiology and Biostatistics, Arba Minch University, Arba Minch, Ethiopia; [35]Department of Emergency and Critical Care Medicine, Haramaya University, Harar, Ethiopia; [36]Department of Epidemiology and Biostatistics, University of Gondar, Gondar, Ethiopia; [37]Department of Public Health, Madda Walabu University, Bale Robe, Ethiopia; [38]Department of Nursing, Arba Minch University, Arba Minch, Ethiopia; [39]Department of Pediatrics and Child Health Nursing, Dilla University, Dilla, Ethiopia; [40]Department of Anatomy, Madda Walabu University, Bale Robe, Ethiopia; [41]Department of Nursing, Dire Dawa University, Dire Dawa, Ethiopia; [42]College of Medicine and Health Sciences, Wollo University, Dessie, Ethiopia; [43]Water, Sanitation and Hygiene Unit, Ethiopian Public Health Institute, Addis Ababa, Ethiopia; [44]Department of Pharmacology, Bahir Dar University, Bahir Dar, Ethiopia; [45]Pharmacy Department, Alkan Health Science, Business and Technology College, Bahir Dar, Ethiopia.

Corresponding Author: Amanuel Yigezu; yigezuamanuel@yahoo.com

Submitting Author: Awoke Misganaw

- Main text word count: Current: 4487



For peer review only - http://bmjopen.bmj.com/site/about/guidelines.xhtml

**Abstract**

**Objective:** This analysis is to present the burden and trends of morbidity and mortality due to LRIs, their contributing risk factors, and the disparity across administrative regions and cities from 1990 to 2019.

**Design:** This analysis is part of a collaborative and comparable systematic Global Burden of Disease (GBD 2019) study.

**Study setting:** The study includes nine region states and two chartered cities of Ethiopia

**Outcome Measures:** We calculated incidence, death, and years of life lost (YLLs) due to LRIs and contributing risk factors using all accessible data sources. We calculated 95% uncertainty intervals (UI) for the point estimates.

**Results:** In 2019, LRIs incidence, death, and YLLs among all age groups were 8313.7 (95% UI: 7757.6–8918), 59.4 (49.8–71.4) and 2404.5 (2059.4–2833.3) per 100000 people, respectively. From 1990, the corresponding decline rates were 39%, 61%, and 76%, respectively. Children under the age of five years account for 20% of episodes, 42% of mortalities, and 70% of the YLL of the total burden of LRIs in 2019. The mortality rate was significantly higher in predominantly pastoralist regions–Benishangul-Gumuz 101.8 (84.0–121.7) and Afar 103.7 (86.6–122.6). The Somali region showed the least decline in mortality rates. More than three-fourths of under-five child deaths due to LRIs were attributed to malnutrition. Household air pollution from solid fuel attributed to nearly half of the risk factors for all age mortalities due to LRIs in the country.

**Conclusion:** In Ethiopia, LRIs have reduced significantly across the regions over the years (except in elders), however are still the third leading cause of mortality, disproportionately affecting children younger than five years old, and predominantly pastoralist regions. Interventions need to consider leading risk factors, targeted age groups and pastoralist and cross-border communities.

***Keywords****: Lower respiratory infections, regions, chartered cities, Ethiopia*



For peer review only - http://bmjopen.bmj.com/site/about/guidelines.xhtml

***What is already known on this topic***

Lower respiratory infections (LRIs) remain a significant contributor to Ethiopia's morbidity and mortality. LRIs are one of the leading causes of under-five mortality and are expected to increase because of the current conflict, internal displacements, health care service interruption and the COVID-19 pandemic. This work is among the first to present a comprehensive analysis of LRIs effect on morbidity and mortality, and their risk factors across all the regional states of Ethiopia over an extended time period to support health decision making at subnational levels.

***What this study adds***

This is the first collaborative effort to provide the effects of LRIs and its risk factors on morbidity and mortality for Ethiopia's nine regions and two chartered cities from 1990 to 2019. This analysis highlights substantial decline morbidities and mortalities caused by LRIs over the last three decades, as well as the disparities between regions and chartered cities, and the opportunity to reduce LRIs burden of premature mortality by addressing specific geographic locations, risk factors and age-groups with cost-effective interventions.

***How this study might affect research, practice or policy***

The results identify regions and segments of the population highly affected by LRIs , and the leading risk factors contributing to premature mortalities. These could be potential priorities for action against LRIs that would reduce premature mortality. There is a need to enhance strategies addressing predominantly pastoralist regions and cross-border communities and for quality health-care services in underserved areas. Although morbidity and mortality have shown improvements over the past three decades, LRIs burden is still high in parts of Ethiopia.



For peer review only - http://bmjopen.bmj.com/site/about/guidelines.xhtml

## INTRODUCTION

Lower Respiratory Infections (LRIs) have been a predominant health problem worldwide, causing more than 2.3 million deaths in 2016 alone, amounting to a mortality rate of 32.2 per 100,000 people (1). LRIs comprise diseases of the lower airways such as pneumonia, bronchitis, and bronchiolitis, among others (2). Nearly all (99%) of LRI deaths occur in low and middle income countries and highly affect children under the age of 5 years (3). In sub-Sahara African countries, the mortality rate is 66.4 per 100,000 people, which is four times the mortality rate in East Asia and twice the global average (2).

Ethiopia ranked in the top three African countries in the number of under-five child deaths from LRIs (4). To prevent child death from LRIs and other diseases in early life, Ethiopia has been implementing the "Integrated management of childhood illness" program since 1997 which was scaled up to the national level in 2007 (5). LRIs, and pneumonia in particular, have been among the top three leading causes of childhood mortality in the country (6). Among LRIs' aetiologies, S. pneumonia contributed to more deaths than the other LRIs aetiologies combined (3). In 2011, the country introduced 10-valent pneumococcal conjugate vaccine (PCV 10) into its national immunization program to reduce the burden of Streptococcal pneumonia (7).

Morbidity and mortality from LRIs are attributable to multiple underlying factors. Malnutrition is one of the main underlying risk factors (8, 9). The other main attributable factor are poor living conditions that include household crowding, parental smoking, high use of household solid fuel/biomass consumption, poor ventilation, and lack of hand-washing facilities (10-12). In addition, bottle feeding also contributes to the burden of LRIs in children (13).

The flagship Ethiopian Health Extension Program (HEP) has been the backbone of the country's health system strategies to reduce the burden of LRIs and other diseases through preventive and


For peer review only - http://bmjopen.bmj.com/site/about/guidelines.xhtml

health promotion activities at the community level. The HEP has also improved broader access to health care, availability of essential antibiotics, and immunization mainly to the rural population since 2004 (14). In 2010, the country also introduced the "integrated community case management" (ICCM) approach to treat pneumonia through trained health cadres of health extension workers (HEWs) implementing HEP (15).

Currently, Ethiopia is implementing its Health Sector Transformation Plan-2 (HSTP-2) which is adapted from the Sustainable Development Goals. Some of the aims include increasing the proportion of under-five children with pneumonia who received antibiotics from 48% to 69% and improving full vaccination coverage from 44% to 69% between 2020 and 2025 (16). Commitment to international goals such as The Global Action Plan for the Prevention and Control of Pneumonia and Diarrhoea by 2025 could be reached if enough investment is made in high LRI burden countries like Ethiopia (17).

As Ethiopia is a country of stark contrasts in socioeconomic, epidemiological, and geographical variations, estimating disease burden at the regional level could provide valid and reliable information to inform policy decisions, including efficient resource allocation to match the burden in the subnational states. Hence, this paper presents the 2019 Global Burden of Diseases, Injuries, and Risk Factors study (GBD) results on the burden, trends and regional variations of LRIs in Ethiopia from 1990 to 2019.

## METHODS

### Study Setting

Ethiopia is the second-most populous country in Africa next to Nigeria, with an estimated population of 112 million in 2019 (18). More than half of the country's population is under 20, and



For peer review only - http://bmjopen.bmj.com/site/about/guidelines.xhtml

over 80% of the population resides in rural areas (19). The country is subdivided into ten regional states (Afar, Amhara, Benishangul-Gumuz, Gambella, Harari, Oromia, Somali, Sidama, Southern Nations and Nationalities and Peoples (SNNP), and Tigray) and two chartered cities (Addis Ababa and Dire Dawa). During this study, Sidama was a zonal administration under the SNNP region. Oromia, Amhara, and SNNP are the highly populated regions. In this study, we classified the regions into urban (Addis Ababa, Dire Dawa, and Harari), agrarian (Oromia, Amhara, SNNP, Tigray), and pastoralist (Benishangul-Gumuz, Afar, Gambella, and Somali). The healthcare system of the country is a three-tiered system consisting of primary, secondary, and tertiary levels of healthcare delivery units with 21,154 functioning health facilities and 159,545 health workforce in 2019 (16). The primary health care unit (PHCU) consists of health posts (staffed by HEWs), health centres, and primary hospitals. The secondary level of care consists of general hospitals and the tertiary level of care includes national referral hospitals which provide specialized services (16).

**Data Sources and Analysis**

The analysis and findings of LRIs presented in this paper were produced by the Ethiopia Subnational Burden of Disease Initiative, a collaborative endeavour between the National Data Management Center for Health (NDMC) at the Ethiopian Public Health Institute (EPHI) and the Institute for Health Metrics and Evaluation (IHME), as part of GBD. The details of the methodology were described elsewhere (20). In brief, woreda (district) level geographic boundary mapping of regions and cities was used because woredas were relatively stable government structures (compared to lower or higher level administrative structures) during political or government changes and through the three census years (1984, 1994, and 2007). First, the analysis was estimated by mapping population and demography at the district level by time and region.



For peer review only - http://bmjopen.bmj.com/site/about/guidelines.xhtml

Then, the data sources were mapped by regions before processing the data in the GBD analysis based on GBD protocol. EPHI, in collaboration with IHME, gathered all accessible data sources by location for Ethiopia and all regions and cities that included census, demographic surveillance, household surveys, diseases registry, health service utilization, disease notification, and other data for this analysis. A comprehensive description of data sources, quality, and modelling for GBD 2019 has been reported on the following online portal: (http://ghdx.healthdata.org/gbd-2019/data-input-sources).

**GBD Methods and Tools**

The GBD details are reported elsewhere (21). Diseases and injuries within the GBD were organized into levels: Level 1 being the broadest causes of death and disability to Level 4 being the most specific. Within the three Level 1 causes (communicable, maternal, neonatal, and nutritional diseases; non-communicable diseases; and injuries), there were 174 Level 3 causes. The GBD 2019 study has estimated the burden of disease, including LRIs, for Ethiopia's national and subnational states. LRIs comprise diseases of the lower airways such as pneumonia, bronchitis, and bronchiolitis, among others (2). LRI mortality was estimated by age, sex, geography, and year using a modelling platform called the Cause of Death Ensemble model. LRI morbidity, including incidence, was modelled using a meta-regression platform known as DisMod-MR, a Bayesian, hierarchical, mixed-effects meta-regression platform (22). Years of life lost (YLLs) were computed by multiplying cause-specific deaths by the life expectancy at the age of death (23, 24). Population risk assessments over time and among risks were estimated using the comparative risk assessment approach developed for the GBD study (25, 26). The GBD risk factors were categorized as follows: Level 1 risk factors are behavioural, environmental, occupational, and


For peer review only - http://bmjopen.bmj.com/site/about/guidelines.xhtml

metabolic; Level 2 risk factors include 20 clusters of risks; Level 3 consists of 52 clusters of risks; and Level 4 contains 69 specific risk factors (21).

**Presentation of results**

We present the burden of LRIs in Ethiopia and its regional states using incidence, deaths, and YLLs categorized by sex, age groups, and year. We used numbers, rates, and percent change for the quantification of the burden. We also estimated the risk factors contributing to LRIs in Ethiopia and the percent change between 1990 and 2019. We reported GBD causes and risk factors using level 3 classifications, with 95% Uncertainty Intervals (UI). Additional tables and figures are attached in the supplementary appendix.

**Patient and public involvement**

Patients and the public were not involved in this study.

## RESULTS

**Morbidity Due to LRIs**

In 2019, an estimated 6,628,673.6 (95% UI: 6,108,786.2–7,230,986.3) new cases of LRIs occurred in Ethiopia resulting in an age-standardized incidence rate of 8313.7 per 100,000 people (7757.6–8918). Out of the total LRI episodes, 22% (1,448,680.0 new cases [1,150,089.8–1,799,704.4]) of new cases occurred among children younger than 5 years, yielding an annual incidence rate of 8685.0 per 100,000 children (6895.1–10,789.8). In adults older than 70 years, there were 725,273.3 (640,315.4–837,746.3) new cases of LRIs with an annual incidence of 38,394.4 per 100,000 people (33,896.9–44,348.5) (Appendix Table 1 and Table 2).



For peer review only - http://bmjopen.bmj.com/site/about/guidelines.xhtml

Compared to national estimates, a significantly lower age-standardized incidence rate of LRIs per 100,000 people was observed in the chartered cities (6788.1 [6285.1–7339.1] in Addis Ababa, 7148.8 [6634.6–7750.7] in Dire Dawa), and in Harari region (7190.5 [6684.1–7718.6]). The highest rates of age-standardized incidence per 100,000 people were observed in Afar (9350.2 [8648.8–10,157.4]), Somali (9220.0 [8515.9–10,046.4]), and Benishangul-Gumuz (9054.6 [8394.2–9766.0]) although not significant compared to the national estimate (Figure 1; Figure 2; Appendix Table 2).

In children younger than 5 years, the lowest incidence rates were observed in Addis Ababa (4927.2 [3812.5–6231.8]), Harari (6821.6 [5373.5–8607.3]), and Gambella (6791.1 [5370.4–8460.1]) per 100,000 people, substantially below the national estimate. The highest incidence of LRIs per 100,000 children were recorded in Benishangul-Gumuz (10,481.8 [8269.5–13,219.0]), Somali (10,062.7 [7949.6–12,802.8]), Afar (9365.5 [7421.7–11,714.7]), and Oromia (9039.4 [7080.5–11,277.7]).

The age-standardized decline rate between 1990 and 2019 was 39% for both sexes, and it was 56% in under-five children and 13% in adults older than 70. The lowest decline in age-standardized incidence rate was in Somali (19%); while it was between 38% and 44% for the remaining regions (Appendix Table 2).

In children younger than 5, the incidence rates increased slightly between 1990 and 1995, except in Addis Ababa and Amhara region. The highest decline rates were found between the years 2005 and 2015 across all regions (Appendix Figure 1).

**Mortality due to LRIs**



For peer review only - http://bmjopen.bmj.com/site/about/guidelines.xhtml

248 In 2019, LRIs caused 46,300.7 (95% UI: 39,515–54,642) deaths in Ethiopia, giving the age-standardized mortality rate of 86.4 (75.3-97.6) per 100,000 people. Under-five mortality accounted for 42% (19,591.8 [14,018.4–26,899.0]) of all deaths due to LRIs which resulted in a mortality rate of 117.4 (84.0–161.2) per 100,000 children. Of the under-five deaths, 71% (13,919.0 [9946.0–18,860.0]) occurred in the first year of life. In adults older than 70 years, LRIs caused 14,627.6 (12,393.7–16,892.8) deaths, which was a mortality rate of 774.3 (656–894.2) deaths per 100,000 adults (Table 1; Appendix Table 3).

In 2019, the number of deaths in all age groups by region were highest in Oromia (18,206 [15,193–21745]), Amhara (9525 [7530–11872]), SNNP (9494 [7713–11649]), and Somali (3907.5 [3007.4–4959.4]), followed by Tigray (2551.4 [2090.3–3028.6]), Afar (706.2 [567.7–869.6]), Benishangul-Gumuz (619 [470.7–803.0]), Dire Dawa (154.6 [120.6–193.8]) and Gambella (123.2 [98.2–151.9]). Harari (93.1 [73.8–116.0) had the lowest number of deaths. (Appendix Table 3).

Between 1990 and 2019, the age-standardized mortality rate declined by 61%, and the decline in under-five children and adults over 70 years was 86% and 30%, respectively (Table 1).

For peer review only



For peer review only - http://bmjopen.bmj.com/site/about/guidelines.xhtml

*Table 1: Lower Respiratory Infections mortality rates and percentage changes between 1990 and 2019 in Ethiopia, both sexes, with different age groups*

| | Age standardized | | | Children younger than 5 | | | People older than 70 | | |
|---|---|---|---|---|---|---|---|---|---|
| Location | Deaths per 100 000 people [95% UI], 1990 | Deaths per 100 000 people [95% UI], 2019 | Cha-nge, % | Deaths per 100 000 children [95% UI], 1990 | Deaths per 100 000 children [95% UI], 2019 | Cha-Nge,% | Deaths per 100 000 people [95% UI], 1990 | Deaths per 100 000 people [95% UI], 2019 | Cha-nge,% |
| Addis Ababa | 163.4[134.6-206.9] | 59.4[49.8-71.4] | 64 | 441.5[320.4-602.7] | 23.4[14.1-36.4] | 95 | 909.2[669.5-1264.6] | 503.6[410.5-633.7] | 45 |
| Oromia | 241.3[188.5-292.8] | 89.3[75.9-103.1] | 63 | 920.7[629.5-1264.1] | 122.3[84.8-171] | 87 | 1174.1[842.8-1543] | 849.5[687.3-1009] | 28 |
| Amhara | 197.7[163.5-236.5] | 74.3[59.4-91] | 62 | 691.3[531.7-883.4] | 94.9[55.6-146.1] | 87 | 1017.9[755.4-1324] | 682.7[537.4-857.9] | 33 |
| SNNPs | 243.7[196.4-296.9] | 98.9[83.8-116.6] | 59 | 971.9[697.2-1273.2] | 118.4[79-168.5] | 88 | 1161.3[584.7-822.3] | 845.4[685.6-018.1] | 28 |
| Tigray | 242.7[200.5-292] | 84.6[69.2-100.1] | 65 | 780.4[593.5-1009.1] | 67.2[43.5-98.3] | 92 | 1166.7[838.2-1586] | 788.6[637.9-956.8] | 33 |
| Harari | 240.2[182.6-303.1] | 77[63-92.5] | 68 | 1146.7[730-1604.1] | 89.9[51.1-138] | 93 | 762.3[412-1223.7] | 694.3[551.9-849] | 9 |
| Afar | 244.8[186.5-316.9] | 103.7[86.6-122.6] | 58 | 723.7[481.5-1023.7] | 101.7[65-152.4] | 86 | 1010[658-1541.7] | 917.6[720.6-146.8] | 10 |
| Somali | 147.6[113.8-192.6] | 97.5[79.2-118.6] | 34 | 455.9[313-628.5] | 197.3[135-279.8] | 57 | 798.8[519.2-1177.6] | 765.1[588.8-972.4] | 5 |
| BG | 284.1[221.8-358.1] | 101.8[84-121.7] | 64 | 1266.4[846.7-790.9] | 215.1[141.4-311] | 84 | 975.3[656.5-1379.3] | 671.9[532.7-848.7] | 32 |
| Dire Dawa | 220.1[171.6-270.9] | 69.9[56.5-84] | 68 | 1084.2[689.1-503.2] | 83.7[44.7-135.9] | 93 | 868.7[604.5-1205.5] | 621.3[496.4-766.1] | 29 |
| Gambella | 231.5[176.8-297.4] | 82.4[68.3-97.1] | 64 | 1265.5[805.4-764.3] | 57[32.4-89.6] | 96 | 739.6[477.7-1074.9] | 678.3[540.4-835.3] | 9 |
| Ethiopia* | 223[184.7-264.3] | 86.4[75.3-97.6] | 61 | 822.2[635-1051.2] | 117.4[84-161.2] | 86 | 1092.3[844-1391.5] | 774.3[656-894.2] | 30 |

N.B: all changes are in decreasing percent. SNNPs: Southern Nations, Nationalities, and Peoples; BG: Benishangul-Gumuz; *national estimate

For peer review only



For peer review only - http://bmjopen.bmj.com/site/about/guidelines.xhtml

In Ethiopia, the age-standardized mortality rate was higher among males (100.6 [84.1-121.4]) than females (71.8 [60.2–82.9]) (Appendix Table 4).

In 1990, the age-standardized mortality rate per 100,000 people was the highest in Benishangul-Gumuz (284.1 [221.8–358.1]), Afar (244.8 [186.5–316.9]) and SNNP (243.7 [196.4–296.9]), although it is not significantly different from the national estimate. On the other hand, the lowest age standardized mortality rate was exhibited in Somali (147.6 [113.8–192.6]). Although the value was not significantly different from the national value in 1990, Addis Ababa showed the second lowest age-standardized mortality rate (163.4 [134.6–206.9]) (Table 1).

In 2019, the age-standardized mortality rate per 100,000 people was significantly lower in Addis Ababa (59.4 [49.8–71.4]) when compared to other regions, although not significantly lower than Gambella and other urban areas (Harari and Dire Dawa). Dire Dawa showed a significantly lower age-standardized mortality rate when compared to Benishangul-Gumuz, Afar, and SNNP. Compared to the national estimate, the regions of Afar (103.7 [86.6–122.6]) and Benishangul-Gumuz (101.8 [84.0–121.7]) recorded the highest age-standardized mortality rates per 100,000 people, although the difference is not statistically significant (Table 1). Compared to the 1990s, there was a 58% to 68% decrease in the age-standardized mortality rates across all regions. However, the Somali region recorded a 34% reduction in mortality rates (Table 1 and Figure 3).

Among children below the age of 5, the mortality rate per 100,000 people was significantly lower in Addis Ababa (23.4 ([14.1–36.4]) than other regions, although it was not significantly less than Gambella in 2019. The mortality rate was the highest in Benishangul-Gumuz (215.1 [141.4–311.0]), Somali (197.3 [135.2–279.8]), and Oromia (122.3 [84.8–171.0]) despite being not significantly higher than the national estimate (Table 1). Dire Dawa showed a significantly lower mortality rate than Benishangul-Gumuz and Somali. Harari had a significantly lower mortality rate



For peer review only - http://bmjopen.bmj.com/site/about/guidelines.xhtml

than Benishangul-Gumuz. For all regions, the mortality rate declined for children younger than 5-years between 1990 and 2019 by between 84% (in Benishangul-Gumuz) and 96% (in Gambella), except in Somali, which showed a 57% decline (Table 1). The mortality rate increased slightly between 1990 and 1995 and between 2010 and 2015 in Somali region (Appendix Figure 2).

The mortality rate in adults older than 70 was the lowest in Addis Ababa (503.6 [410.5–633.7]). Other regions and cities have not shown a statistically significant difference from the national estimate. The decline in mortality rates between 1990 and 2019 is below 50% across all regions (Table 1). The mortality rate increased in Afar and Somali between 2005 and 2019 and in Gambella between 1990 and 2005 (Appendix Figure 3).

**Premature Mortality due to LRIs**

In 2019, premature death due to LRIs was 2,445,093.7 (95% UI: 1,934,420.8–3,119,838.6) YLLs, yielding an age-standardized rate of 2404.5 per 100,000 people (2059.4–2833.3). Compared to 1990, the age-standardized YLL rate declined by 76% in 2019. In parallel, 70% of all premature mortality occurred in children younger than five, which accounted for 1,721,122.3 (1,231,032.1–2,362,958.7) YLLs. The YLL rate of 72,055.4 (55,718.5–92,064) per 100,000 under-five children in 2019 declined by 86% compared to the YLL rate in 1990. Adults over 70 years contributed 8% of all YLLs due to LRIs (194,756.2 [165,462.0–225,502.1]), yielding a rate of 10,309.9 (8759.2–11,937.6) YLLs per 100,000 people (Table 2; Appendix Table 5 and Table 6).

Compared to 1990, the number of YLLs has decreased by 70% in all age groups and by 76% in children younger than five in 2019. However, the number of YLLs has increased by 45% in adults older than 70 (Appendix Table 5).



For peer review only - http://bmjopen.bmj.com/site/about/guidelines.xhtml

The age-standardized YLL rate was significantly lower in Addis Ababa (1285.6 [1065–1561.8]) in 2019 compared to the national average. The highest age standardized YLL rate were in Benishangul-Gumuz (3571.1 [2772.4–4510.7]), Somali (3236.4 [2537.4–4006]), and Afar (2824.6 [2323–3414]), although not statistically significant compared to the national estimate (Table 2).

In children younger than five years, Addis Ababa (2069 [1253–3217.5]) had a significantly lower YLL rate than the national estimate. The YLL rate was observed to be the lowest in Gambella (5031.8 [2867.2–7885.7]), although not significantly lower than the national estimate (Table 2).

For peer review only



For peer review only - http://bmjopen.bmj.com/site/about/guidelines.xhtml

*Table 2: Lower Respiratory Infections YLL rates and percentage changes between 1990 and 2019 in Ethiopia, both sexes, with different age groups*

| | **Age-standardized** | | | **Children younger than 5 years** | | | People older than 70 | | |
|---|---|---|---|---|---|---|---|---|---|
| **Location** | YLL per 100,000 people,1990 | YLL per 100,000 people, 2019 | Change,% | YLL per 100,000 people,1990 | YLL per 100,000 people, 2019 | Change,% | YLL per 100,000 people,1990 | YLL per 100,000 people, 2019 | Change,% |
| Addis Ababa | 6362.2(5161.9-7847.9) | 1285.6(1065-1561.8) | 80 | 38824.7(28230.1-53092.4) | 2069(1253-3217.5) | 95 | 13587(9985.4-18978.3) | 6857.1(5545.8-8721.2) | 50 |
| Oromia | 11217.6(8517-14318) | 2433.8(2042.2-2879.5) | 78 | 80610.7(55103.7-110696) | 10742.8(7456.7-15039.6) | 87 | 18754.2(13297.1-24843) | 11119.7(8939.1-13305) | 41 |
| Amhara | 8690.4(7146.3-10453.8) | 2016.4(1551.4-2541.8) | 77 | 60663.3(46712.5-77511.5) | 8355(4898.9-12845) | 87 | 15950.4(11723.4-21059.7) | 9059.7(7063.2-11529.2) | 44 |
| SNNPs | 11633(9119.4-14477.9) | 2698.1(2243.5-3250.2) | 77 | 85114.7(61203.2-111502.1) | 10409.3(6968.1-14819.3) | 88 | 18842.6(13166.1-26081) | 11736.6(9426.7-14238.6) | 38 |
| Tigray | 10346.4(8522.9-12340.3) | 1977.1(1593.9-2395) | 81 | 68509.3(52185.7-88712.2) | 5927.9(3839.4-8676.8) | 92 | 19502.8(13914.1-26751.2) | 10486.8(8439.5-12765.3) | 47 |
| Harari | 12826.5(9336.5-16985.4) | 2060.4(1595.6-2623.1) | 84 | 100421.7(63898.7-140467.8) | 7922.5(4518.7-12117.2) | 93 | 11782.6(6360.4-19341.1) | 9260.4(7338.7-11340.3) | 22 |
| Afar | 10993.5(8520.8-14193.6) | 2824.6(2323-3414) | 74 | 63570.5(42296.6-89769) | 8943.7(5734.6-13390.2) | 86 | 16925.5(10923.7-26001.7) | 11867.1(9346-14716.9) | 30 |
| Somali | 6286.9(4860.5-7970) | 3236.4(2537.4-4006) | 49 | 40088.1(27543.7-55109) | 17314.3(11890.7-24530) | 57 | 11953.6(7599.2-17885.5) | 10246(7797.8-13150.6) | 15 |
| BG | 14965.1(11197.4-19998.6) | 3571.1(2772.4-4510.7) | 76 | 110847.2(74368-156588.4) | 18868.3(12442.9-27281.8) | 83 | 16397.3(10917-23228.5) | 9541.9(7476.9-12112.3) | 42 |
| Dire Dawa | 11749.7(8605.4-15249.5) | 1832.8(1407.3-2363.1) | 84 | 94905.3(60458.5-131584.5) | 7371.3(3953-11962.1) | 93 | 13207.9(9219.6-18328.2) | 8371(6616.3-10408.2) | 37 |
| Gambella | 13175.8(9291-17411) | 1937.4(1567.6-2344.6) | 85 | 110783.8(70576-154304.1) | 5031.8(2867.2-7885.7) | 96 | 11811.4(7673.5-17012.5) | 10030.9(7730.2-12664.9) | 16 |
| Ethiopia* | 10189.1(8347.5-12201.8) | 2404.5(2059.4-2833.3) | 76 | 72055.4(55718.5-92064) | 10318.6(7380.4-14166.6) | 86 | 17415.9(13362.7-22228.5) | 10309.9(8759.2-11937.6) | 41 |

SNNPs: Southern Nations, Nationalities, and Peoples; BG: Benishangul-Gumuz; *national estimate



For peer review only - http://bmjopen.bmj.com/site/about/guidelines.xhtml

The age-standardized premature mortality rate between 1990 and 2019 showed a continuous decline in all regions, except Somali. Benishangul-Gumuz showed the highest burden between 1990 and 2019 (Figure 4).

The change in premature mortality in children younger than five was significant throughout the years. However, the reduction in premature mortality among adults older than 70 was not as significant (Appendix Figure 4 and Figure 5).

**Risk Factors**

Across all population groups, about half (48%) of the mortalities (measured in rates) due to LRIs in Ethiopia were attributed to household air pollution from solid fuel. In addition, lack of access to a hand-washing facility (23%), childhood wasting (23%), low birth weight (9%), short gestation period (7%), and ambient particulate matter pollution (6%) were also risk factors for mortality due to LRIs. The contribution of the risk factors to death due to LRIs in all regions was similar to the national estimate, except in Addis Ababa, where lack of access to hand-washing facilities, ambient particulate matter pollution, and low temperature were the main contributing factors. Ambient particulate matter pollution was also relatively higher in Dire Dawa, Harari, and Tigray regions (Appendix Figure 6).

In children younger than five, more than three-fourths of deaths due to LRIs were attributed to childhood wasting (54%) stunting (12%), and child underweight (10%) in Ethiopia. In addition, 50% of the LRIs mortalities were attributed to household air pollution from solid fuel. Lack of access to hand-washing facilities (23%), low birth weight (23%), short gestation period (17%), high (4%) or low temperature (4%), absence of exclusive breastfeeding (4%) were also the risk factors with evident contribution. The distribution of risk factors varied among the regions. Child



For peer review only - http://bmjopen.bmj.com/site/about/guidelines.xhtml

wasting, low birth weight, and pre-term birth were the contributing factors of mortality in Addis Ababa. In Harari and Dire Dawa, child wasting, lack of access to hand-washing facilities, pre-term birth, and household air pollution from solid fuels contributed more to the death from LRIs than the other risk factors in 2019. All the risk factors except for ambient particulate air matter and low temperature were the highest in Somali and Benishangul-Gumuz (Appendix Figure 7).

## Discussion

The findings from this study indicate that although the burden of LRIs, measured in incidence or mortality rates, have shown a significant decline, they are still the third leading cause of death after neonatal disorders and diarrheal diseases in 2019 in Ethiopia. Cities and predominantly urban areas had lower mortality rates than predominantly pastoralist regions of the country. The rate of decline in mortality between 1990 and 2019 varied slightly across regions and chartered cities. The mortality rate decreased by more than three-fourths among children under the age of five and only by one-third among adults older than 70 between 1990 and 2019. Half of LRIs mortalities are attributed to household air pollution from solid fuels in all age groups. About three-fourths of LRIs in children were attributed to malnutrition.

The mortality rate of LRIs among children below the age of 5 has declined by 86% between 1990 and 2019. This decline could be attributed to improvements in living conditions, access to healthcare, and immunization. The national health delivery infrastructure has grown from 2,600 health facilities in 1997 to 21,154 facilities, including 314 hospitals, 3,678 health centers, and 17,162 health posts and private health facilities in 2019. As a result, the health workforce has increased from 46,000 in 2007 to 159,545 in 2019 (16). The introduction of HEP since 2004 to provide preventive, health promotion, and curative treatment for pneumonia, malaria and diarrhoea



For peer review only - http://bmjopen.bmj.com/site/about/guidelines.xhtml

had improved health outcomes for children (14). Moreover, Ethiopia has been implementing holistic child health improvement programs like IMCI since 1997 (15) and ICCM (5). Vaccination against Streptococcus pneumonia, which is responsible for about half of the LRIs mortality in African countries, with the PCV 10 vaccine since 2011 helped reduce the burden of LRIs (4). In addition, improved socioeconomic conditions have supported the reduction of the burden of LRIs in the country (27).

Despite the achievements reached in improving child health outcomes in Ethiopia, children are still disproportionately affected by LRIs. Out of the total burden of LRIs, 42% of all the deaths were among children younger than five. In that regard, this study has shown that one out of ten child deaths are due to LRIs. However, another study estimated that pneumonia alone shared about 17% of all deaths in children younger than five years (28)**.** Among children under five, children younger than one carry the highest burden of LRIs. Furthermore, the mortality rate among children younger than one (397.7 per 100,000 people) was more than nine times higher than that of children between one and four years old (43 per 100,000 people). Although most studies on the high burden of LRIs corroborate our findings, the findings from Global Health Observatory (GHO) estimated a much higher mortality rate than the estimates of this study (i.e. 481.9 in children less than one year and 51.1 per 1000,000 population in children between one and four years) (8). Although we could not explain why this variation occurs, the estimates from GBD are also less than the estimate from findings of the Child Health Epidemiology Reference Group (CHERG), which is primarily due to the difference in the types of data used (29).

Urban areas of the country, mainly Addis Ababa and Dire Dawa, had significantly lower mortality rates than pastoralist areas such as Benishangul-Gumuz and Somali. These regional variations could be attributed to gaps in availability and access to healthcare and socioeconomic status



For peer review only - http://bmjopen.bmj.com/site/about/guidelines.xhtml

differences among the subnational states. A previously conducted study indicated that disease burden is high among people in the poorest wealth quintile and people located mainly in Afar, Somali, Oromia, SNNP, and Benishangul-Gumuz and also have the lowest level of health service utilization (30). The rural area of the country has less healthcare coverage and utilization than the urban areas. The coverage of all basic vaccination is 43% in the country. Across the regions, the coverage of all basic vaccination is lowest in Afar (20%) and highest in Addis Ababa (83%). Coverage of pentavalent vaccines in children is 72% among the urban population while it is 56% among the rural population (15, 28). There is a variation in the performance of immunization across regions. Addis Ababa, Ethiopia's capital has PCV3 coverage of 93% among 1-year-olds in 2019, whereas this coverage is only 23% in Afar and Somali. The wasting rate in Somali was 21% in children under five, while it was just 2% in Addis Ababa in 2019 (31)

In addition to children, LRIs affects people older than 70 years. The number of people dying from LRIs increased over the years in people older than 70 years, partly due to the increase in the aging population. However, the mortality rate declined by 30% between 1990 and 2019 (3, 32). The incidence rate did not show a significant improvement across the study years, showing only 15% reductions between 1990 and 2019. Among the regions, Somali and Afar have recorded an increased incidence rate between 1990 and 2019. This could partly be explained by poor accessibility and availability of health facilities in the regions (31).

Wasting, stunting, and underweight were major risk factors contributing to the death of children younger than five years due to LRIs (33-35). More than 37% of children under five are stunted, with a higher percentage in rural areas (41%) than in urban areas (26%). Similarly, the prevalence of child underweight and wasting is 21% in the rural and 7% in the urban parts of Ethiopia (36). This indicates that more investment is needed to reduce the burden on malnutrition among children



For peer review only - http://bmjopen.bmj.com/site/about/guidelines.xhtml

and in the rural parts of the country to attain better health outcomes, protecting against LRIs. Although the prevalence of stunting, wasting, and underweight has decreased markedly over time, they are still major risk factors for death caused by LRIs. In the Millennium Development Goals (MDG) era, between 1990 and 2015, about half of the deaths averted due to LRIs were attributed to improvement in the nutritional status of children (reduction in wasting and stunting) (15).

Ambient particulate matter and household air pollution from solid fuel use were the two essential components of air pollution. Household air pollution from solid fuel use  the was the second leading risk factor for LRIs, and ambient particulate matter is the eight leading risk factor among the top ten risk factors for LRIs  (37-39). This indicates that improvement in socio-demographic factors will have a more substantial contribution as the use of electricity and natural gas for cooking increases (3).

To reduce the burden of LRIs, both national and global efforts are underway. The Global Action Plan for the Prevention and Control of Pneumonia and Diarrhoea (GAPPD), established by the World Health Organization, set goals in 2013 to reduce child LRI mortality rates to below 3 in 1,000 live births and to reduce severe LRI incidence by 75% of the 2010 baseline by 2025. To achieve these goals, reaching 90% of children with full-dose vaccine coverage, 90% access to pneumonia treatment, 50% coverage of exclusive breastfeeding in the first six months, and exclusive breastfeeding promotion were set as prerequisites (17). However, a 6% average annual mortality reduction was recorded between 2000 and 2018 in Ethiopia. With this, the country can only reach the 2025 GAPPD target in 2035, ten years behind the target, according to the Maternal and Child Epidemiology Estimation Group (MICE) estimation (28).

Ethiopia’s major health sector strategic plan, the Health Sector Transformation Plan-2 (HSTP-2) for the year 2021-2024, aims to reduce the infant mortality rate to 35, the neonatal mortality rate



For peer review only - http://bmjopen.bmj.com/site/about/guidelines.xhtml

to 21 and the children under-5 mortality rate to 43 per 1,000 live births. Similarly, the 2030 Sustainable Development Goals (SDG) has set targets to reduce childhood mortality significantly. These targets can be achieved if the country implements high-impact priority curative and preventive interventions against LRIs (40). To achieve this, concerted action to improve policies, increase investment, foster innovations, and scale-up evidence-based interventions has paramount importance. Parallel to this, an estimated $274 billion for health is required to achieve the health-related SDGs by 2030 in 67 low and middle-income countries, including Ethiopia. One of the strategies to mobilize the needed resources is to increase government expenditure by 15% and share the population's costs through taxes or insurances (41).

This study is not without limitations; limitations in the GBD methods also apply to this study. One of the limitations is the incompleteness of data. A previously conducted study showed that most countries in Africa, including Ethiopia, have had a vast amount of incomplete vital records data (42). This increases the uncertainty in the estimates, which might compromise the accuracy of the findings and reduce the use of these findings for policy decision-making (1). However, to improve the quality of the estimates, the best available data are used and GBD estimates are continuously updated.

## Conclusion

Despite the substantial reduction in morbidity and mortality at national and regional states, LRIs still remain one of the leading causes of the burden of disease in Ethiopia. Children and elders are still disproportionately affected by LRIs. The burden of illness and death due to LRIs varies across regional states in Ethiopia, with lower rates in cities and predominantly urban areas while predominantly pastoralist areas of the country have higher rates. Efforts should be made to tackle



For peer review only - http://bmjopen.bmj.com/site/about/guidelines.xhtml

the major risk factors contributing to death by LRIs. Improving child nutrition, access to immunization and curative health services, as well as universal electrification to reduce indoor air pollution will be very useful strategies to reduce deaths due to LRIs in Ethiopia. Furthermore, improvement in socioeconomic factors will also help to reduce LRI burden at national and regional levels. To reach the targets set at the national and international level, mobilizing resources to health and improving the provision of health services to the community according to the needs of the regions is of paramount importance.

## List of abbreviations

EPHI: Ethiopian Public Health Institute; GAPPD: Global Action Plan for the Prevention and Control of Pneumonia and Diarrhoea GBD: Global Burden of Disease; GHO: Global Health Observatory; HSTP: Health Sector Transformation Plan; ICCM: integrated community case management; IHME: Institute of Health and Metric Evaluation; LRIs: Lower Respiratory Infections; NDMC: National Data Management Center for health; PCV: Pneumococcal Conjugate Vaccine; SDG: Sustainable Development Goals; SNNPs: Southern Nations, Nationalities and Peoples; UI: Uncertainty Interval; WHO: World Health Organization; YLL: Years of Life Lost.

**Ethics approval and consent to participate**

This manuscript was produced as part of the GBD Collaborator Network and in accordance with the GBD Protocol.

**Consent for publication**

Not applicable.

**Availability of data and material**



For peer review only - http://bmjopen.bmj.com/site/about/guidelines.xhtml

All relevant data are submitted with this manuscript.

**Competing interests**

The authors declare that they have no competing interests.

**Funding**

Bill and Melinda Gates Foundation has funded Ethiopia subnational burden of disease EPHI-IHME collaborative initiative. The funder of this study had no role in study design, data collection, data analysis, data interpretation, or the writing of the report.

**Authors' contributions**

Please see appendix section C for more detailed information about individual author contributions to the research, divided into the following categories: providing data or critical feedback on data sources; developing methods or computational machinery; providing critical feedback on methods or results; drafting the manuscript or revising it critically for important intellectual content; and managing the estimation or publications process

**Declarations**

M A Gebremichael reports support for the present manuscript from the Bill and Melinda Gates Foundation.

**Acknowledgements**

The authors have no acknowledgements to declare

## References

1. Global, regional, and national age-sex specific mortality for 264 causes of death, 1980-2016: a systematic analysis for the Global Burden of Disease Study 2016. Lancet (London,



For peer review only - http://bmjopen.bmj.com/site/about/guidelines.xhtml

England). 2017 Sep 16;390(10100):1151-210. PubMed PMID: 28919116. Pubmed Central PMCID: PMC5605883. Epub 2017/09/19. eng.

2. Troeger C, Blacker B, Khalil IA, Rao PC, Cao J, Zimsen SRM, et al. Estimates of the global, regional, and national morbidity, mortality, and aetiologies of lower respiratory infections in 195 countries, 1990–2016: a systematic analysis for the Global Burden of Disease Study 2016. The Lancet Infectious Diseases. 2018;18(11):1191-210.

3. Moradi-Lakeh M, El Bcheraoui C, Charara R, Khalil I, Afshin A, Kassebaum NJ, et al. Burden of lower respiratory infections in the Eastern Mediterranean Region between 1990 and 2015: findings from the Global Burden of Disease 2015 study. International Journal of Public Health. 2018 2018/05/01;63(1):97-108.

4. Reiner RC, Welgan CA, Casey DC, Troeger CE, Baumann MM, Nguyen QP, et al. Identifying residual hotspots and mapping lower respiratory infection morbidity and mortality in African children from 2000 to 2017. Nature Microbiology. 2019 2019/12/01;4(12):2310-8.

5. Miller NP, Amouzou A, Tafesse M, Hazel E, Legesse H, Degefie T, et al. Integrated community case management of childhood illness in Ethiopia: implementation strength and quality of care. Am J Trop Med Hyg. 2014;91(2):424-34. PubMed PMID: 24799369. Epub 05/05. eng.

6. Alamneh YM, Adane F. Magnitude and Predictors of Pneumonia among Under-Five Children in Ethiopia: A Systematic Review and Meta-Analysis. Journal of Environmental and Public Health. 2020 2020/05/30;2020:1606783.

7. Wondimu A, Cao Q, Wilschut JC, Postma MJ. Factors associated with the uptake of newly introduced childhood vaccinations in Ethiopia: the cases of rotavirus and pneumococcal conjugate vaccines. BMC Public Health. 2019 2019/12/10;19(1):1656.

For peer review only

8. Gonakoti S, Osifo IF. Protein-Energy Malnutrition Increases Mortality in Patients Hospitalized With Bacterial Pneumonia: A Retrospective Nationwide Database Analysis. Cureus. 2021 Jan 12;13(1):e12645. PubMed PMID: 33585131. Pubmed Central PMCID: PMC7876587. Epub 2021/02/16. eng.

9. Yeo HJ, Byun KS, Han J, Kim JH, Lee SE, Yoon SH, et al. Prognostic significance of malnutrition for long-term mortality in community-acquired pneumonia: a propensity score matched analysis. The Korean journal of internal medicine. 2019 Jul;34(4):841-9. PubMed PMID: 30685962. Pubmed Central PMCID: PMC6610202. Epub 2019/01/29. eng.

10. Adane MM, Alene GD, Mereta ST, Wanyonyi KL. Prevalence and risk factors of acute lower respiratory infection among children living in biomass fuel using households: a community-based cross-sectional study in Northwest Ethiopia. BMC Public Health. 2020 2020/03/19;20(1):363.

11. Adane MM, Alene GD, Mereta ST, Wanyonyi KL. Effect of improved cookstove intervention on childhood acute lower respiratory infection in Northwest Ethiopia: a cluster-randomized controlled trial. BMC Pediatrics. 2021 2021/01/04;21(1):4.

12. Keleb A, Sisay T, Alemu K, Ademas A, Lingerew M, Kloos H, et al. Pneumonia remains a leading public health problem among under-five children in peri-urban areas of north-eastern Ethiopia. PLOS ONE. 2020;15(9):e0235818.

13. Ahmed KY, Page A, Arora A, Ogbo FA, Global M, Child Health Research c. Associations between infant and young child feeding practices and acute respiratory infection and diarrhoea in Ethiopia: A propensity score matching approach. PLOS ONE. 2020;15(4):e0230978.



For peer review only - http://bmjopen.bmj.com/site/about/guidelines.xhtml

14. Assefa Y, Gelaw YA, Hill PS, Taye BW, Van Damme W. Community health extension program of Ethiopia, 2003–2018: successes and challenges toward universal coverage for primary healthcare services. Globalization and Health. 2019 2019/03/26;15(1):24.

15. Ruducha J, Mann C, Singh NS, Gemebo TD, Tessema NS, Baschieri A, et al. How Ethiopia achieved Millennium Development Goal 4 through multisectoral interventions: a Countdown to 2015 case study. Lancet Glob Health. 2017;5(11):e1142-e51. PubMed PMID: 29025635. eng.

16. Federal Ministry of Health. Health Sector Transformation Plan 2020/21-2024/25 (2013 EFY - 2017 EFY), Addis Ababa Ethiopia. 2020.

17. Unicef, World Health O. Ending preventable child deaths from pneumonia and diarrhoea by 2025 : the integrated global action plan for pneumonia and diarrhoea (GAPPD) 2013. Available from: https://apps.who.int/iris/handle/10665/79200.

18. World B. World Bank data catalog. [Washington, D.C.]: World Bank.

19. CSA. Ethiopia. central statstical agency. 2020.

20. Misganaw ANMWAMAHGAZBTMWEAKJHEGTWTKDDA. Progress in health among regions of Ethiopia, 1990-2019: a subnational country analysis for the Global Burden of Disease Study 2019. Lancet Lancet (London, England). 2022;399(10332):1322-35. English.

21. Murray CJL, Aravkin AY, Zheng P, Abbafati C, Abbas KM, Abbasi-Kangevari M, et al. Global burden of 87 risk factors in 204 countries and territories, 1990–2019: a systematic analysis for the Global Burden of Disease Study 2019. The Lancet. 2020;396(10258):1223-49.

22. Collaborators GL. Estimates of the global, regional, and national morbidity, mortality, and aetiologies of lower respiratory tract infections in 195 countries: a systematic analysis for the Global Burden of Disease Study 2015. Lancet Infect Dis. 2017;17(11):1133-61. PubMed PMID: 28843578. Epub 08/23. eng.



For peer review only - http://bmjopen.bmj.com/site/about/guidelines.xhtml

23. DALYs GBD, Collaborators H. Global, regional, and national disability-adjusted life-years (DALYs) for 315 diseases and injuries and healthy life expectancy (HALE), 1990-2015: a systematic analysis for the Global Burden of Disease Study 2015. Lancet (London, England). 2016;388(10053):1603-58. PubMed PMID: 27733283. eng.

24. Dicker D, Nguyen G, Abate D, Abate KH, Abay SM, Abbafati C, et al. Global, regional, and national age-sex-specific mortality and life expectancy, 1950–2017: a systematic analysis for the Global Burden of Disease Study 2017. The Lancet. 2018;392(10159):1684-735.

25. Lim SS, Vos T, Flaxman AD, Danaei G, Shibuya K, Adair-Rohani H, et al. A comparative risk assessment of burden of disease and injury attributable to 67 risk factors and risk factor clusters in 21 regions, 1990-2010: a systematic analysis for the Global Burden of Disease Study 2010. Lancet (London, England). 2012;380(9859):2224-60. PubMed PMID: 23245609. eng.

26. Collaborators GBDRF, Forouzanfar MH, Alexander L, Anderson HR, Bachman VF, Biryukov S, et al. Global, regional, and national comparative risk assessment of 79 behavioural, environmental and occupational, and metabolic risks or clusters of risks in 188 countries, 1990-2013: a systematic analysis for the Global Burden of Disease Study 2013. Lancet (London, England). 2015;386(10010):2287-323. PubMed PMID: 26364544. Epub 09/11. eng.

27. Shibre G, Zegeye B, Idriss-Wheeler D, Yaya S. Trends of inequalities in care seeking behavior for under-five children with suspected pneumonia in Ethiopia: evidence from Ethiopia demographic and health surveys (2005–2016). BMC Public Health. 2021 2021/02/01;21(1):258.

28. UNICEF. Fighting for Breath in Ethiopia: a call to action to stop children dying from pneumonia. 2020.

29. Kovacs SD, Mullholland K, Bosch J, Campbell H, Forouzanfar MH, Khalil I, et al. Deconstructing the differences: a comparison of GBD 2010 and CHERG's approach to estimating

For peer review only



For peer review only - http://bmjopen.bmj.com/site/about/guidelines.xhtml

the mortality burden of diarrhea, pneumonia, and their etiologies. BMC Infectious Diseases. 2015 2015/01/16;15(1):16.

30. Federal Ministry of Health E. EVIDENCE SYSTHESIS BASED ON DHS KEY MCH AND NUTRITION INDICATORS. 2019.

31. Health FDRoE-Mo. Mini Demographic and Health Survey 2019. 2019.

32. Corrêa RA, José BPS, Malta DC, Passos VMA, França EB, Teixeira RA, et al. Burden of disease by lower respiratory tract infections in Brazil, 1990 to 2015: estimates of the Global Burden of Disease 2015 study. Revista brasileira de epidemiologia = Brazilian journal of epidemiology. 2017 May;20Suppl 01(Suppl 01):171-81. PubMed PMID: 28658381. Epub 2017/06/29. Carga de doença por infecções do trato respiratório inferior no Brasil, 1990 a 2015: estimativas do estudo Global Burden of Disease 2015. por

eng.

33. Salam RA, Das JK, Bhutta ZA. Current issues and priorities in childhood nutrition, growth, and infections. The Journal of nutrition. 2015 May;145(5):1116S-22S. PubMed PMID: 25833888. Pubmed Central PMCID: PMC4410495. Epub 2015/04/03. eng.

34. Akombi BJ, Agho KE, Hall JJ, Wali N, Renzaho AMN, Merom D. Stunting, Wasting and Underweight in Sub-Saharan Africa: A Systematic Review. International journal of environmental research and public health. 2017 Aug 1;14(8). PubMed PMID: 28788108. Pubmed Central PMCID: PMC5580567. Epub 2017/08/10. eng.

35. Ibrahim MK, Zambruni M, Melby CL, Melby PC. Impact of Childhood Malnutrition on Host Defense and Infection. Clinical microbiology reviews. 2017 Oct;30(4):919-71. PubMed PMID: 28768707. Pubmed Central PMCID: PMC5608884. Epub 2017/08/05. eng.



For peer review only - http://bmjopen.bmj.com/site/about/guidelines.xhtml

36. Fantay Gebru K, Mekonnen Haileselassie W, Haftom Temesgen A, Oumer Seid A, Afework Mulugeta B. Determinants of stunting among under-five children in Ethiopia: a multilevel mixed-effects analysis of 2016 Ethiopian demographic and health survey data. BMC Pediatrics. 2019 2019/06/01;19(1):176.

37. Dherani M, Pope D, Mascarenhas M, Smith KR, Weber M, Bruce N. Indoor air pollution from unprocessed solid fuel use and pneumonia risk in children aged under five years: a systematic review and meta-analysis. Bulletin of the World Health Organization. 2008 May;86(5):390-8C. PubMed PMID: 18545742. Pubmed Central PMCID: PMC2647443. Epub 2008/06/12. eng.

38. Kurmi OP, Lam KB, Ayres JG. Indoor air pollution and the lung in low- and medium-income countries. The European respiratory journal. 2012 Jul;40(1):239-54. PubMed PMID: 22362845. Epub 2012/03/01. eng.

39. Burnett RT, Pope CA, 3rd, Ezzati M, Olives C, Lim SS, Mehta S, et al. An integrated risk function for estimating the global burden of disease attributable to ambient fine particulate matter exposure. Environmental health perspectives. 2014 Apr;122(4):397-403. PubMed PMID: 24518036. Epub 2014/02/13. eng.

40. Federal Ministry of Health Addis Ababa E. Health Sector Transformation Plan 2015/16 - 2019/20 (2008-2012 EFY). . August 2015. .

41. Stenberg K, Hanssen O, Edejer TT-T, Bertram M, Brindley C, Meshreky A, et al. Financing transformative health systems towards achievement of the health Sustainable Development Goals: a model for projected resource needs in 67 low-income and middle-income countries. The Lancet Global Health. 2017;5(9):e875-e87.



For peer review only - http://bmjopen.bmj.com/site/about/guidelines.xhtml

42. Byass P, de Courten M, Graham WJ, Laflamme L, McCaw-Binns A, Sankoh OA, et al. Reflections on the global burden of disease 2010 estimates. PLoS Med. 2013;10(7):e1001477-e. PubMed PMID: 23843748. Epub 07/02. eng.

For peer review only



For peer review only - http://bmjopen.bmj.com/site/about/guidelines.xhtml

637 *Figure 1: Incidence of lower respiratory infections per 100,000 people in Ethiopia and its regions in 2019.*

639 *Figure 2: Trend in age-standardized incidence rate of LRIs per 100,000 people in Ethiopia, 1990-2019*

641 *Figure 3: Trend in LRIs age-standardized mortality rates per 100,000 people in Ethiopia, 1990-2019*

643 *Figure 4: Trend in LRIs age standardized years of life lost rates per 100,000 people in Ethiopia, 1990-2019*

645

For peer review only



For peer review only - http://bmjopen.bmj.com/site/about/guidelines.xhtml

*Figure 1: Incidence of lower respiratory infections per 100,000 people in Ethiopia and its regions in 2019.*

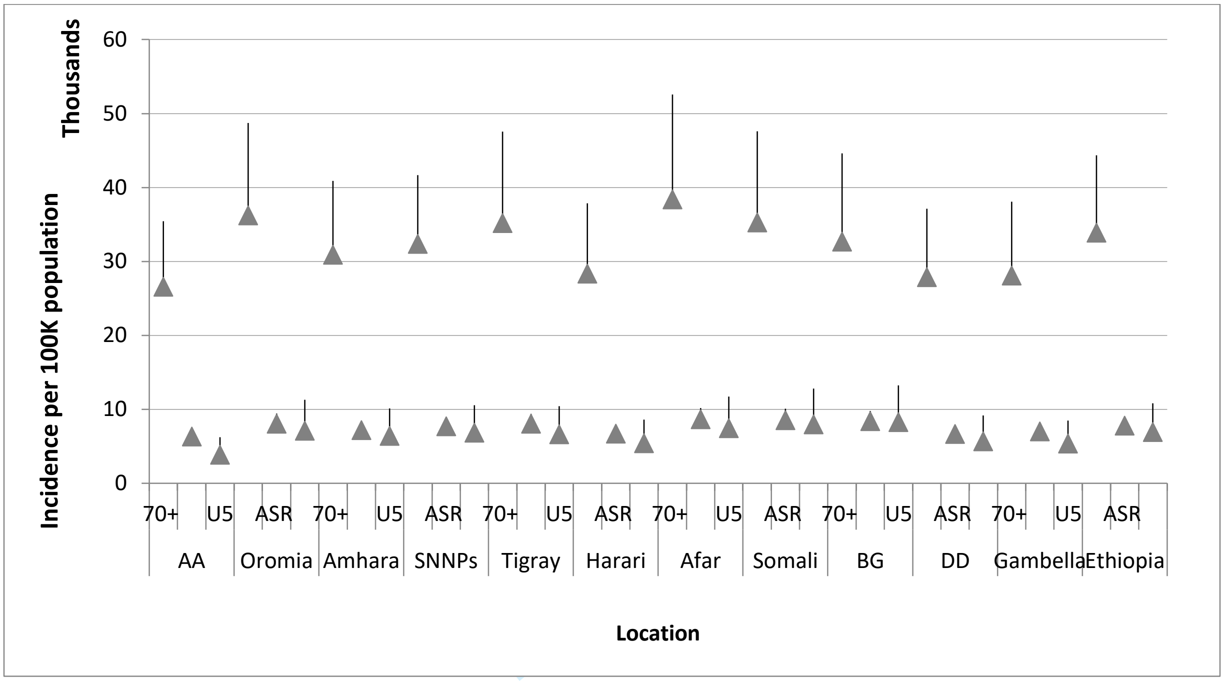


*Key: U5: children younger than 5 years; 70+: adults older than 70 years; ASR: Age-standardized rates; AA: Addis Ababa; DD: Dire Dawa; SNNPs: Southern Nations and Nationalities and Peoples; BG: Benishangul Gumuz*

For peer review only

*Figure 2: Trend in age-standardized incidence rate of LRIs per 100,000 people in Ethiopia, 1990-2019*

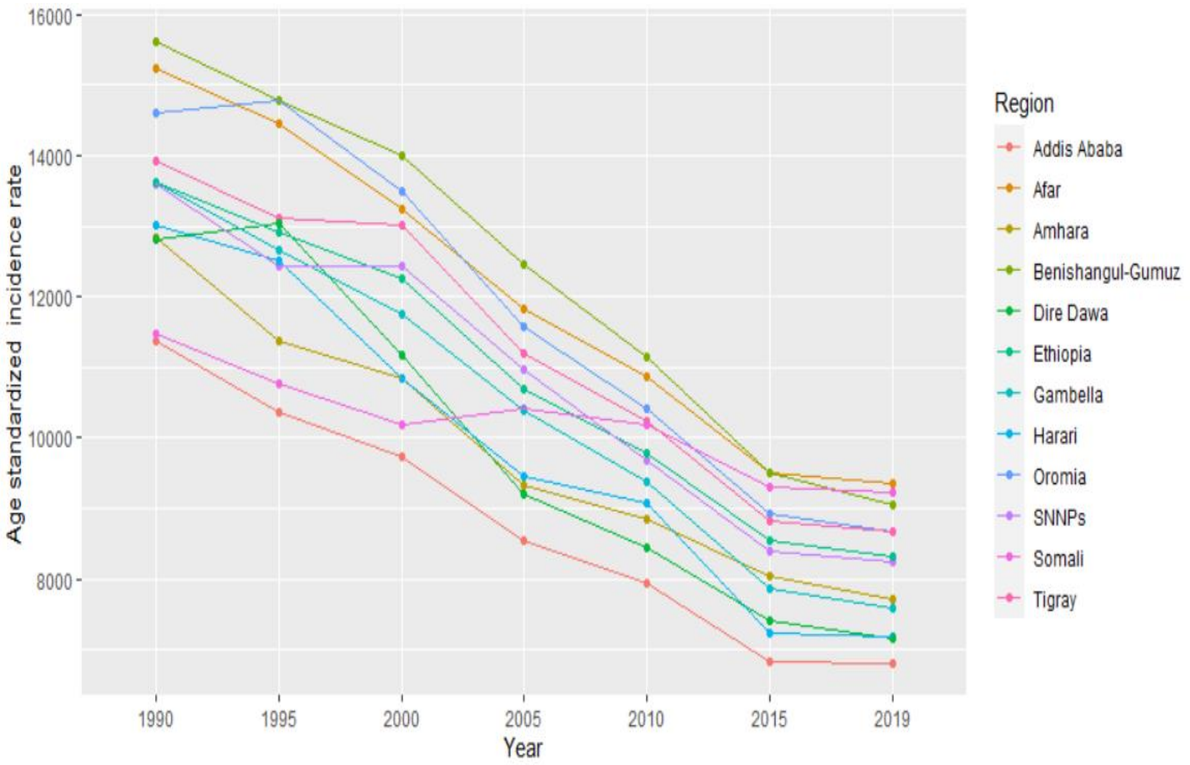

*Figure 3: Trend in LRIs age-standardized mortality rates per 100,000 people in Ethiopia, 1990-2019*

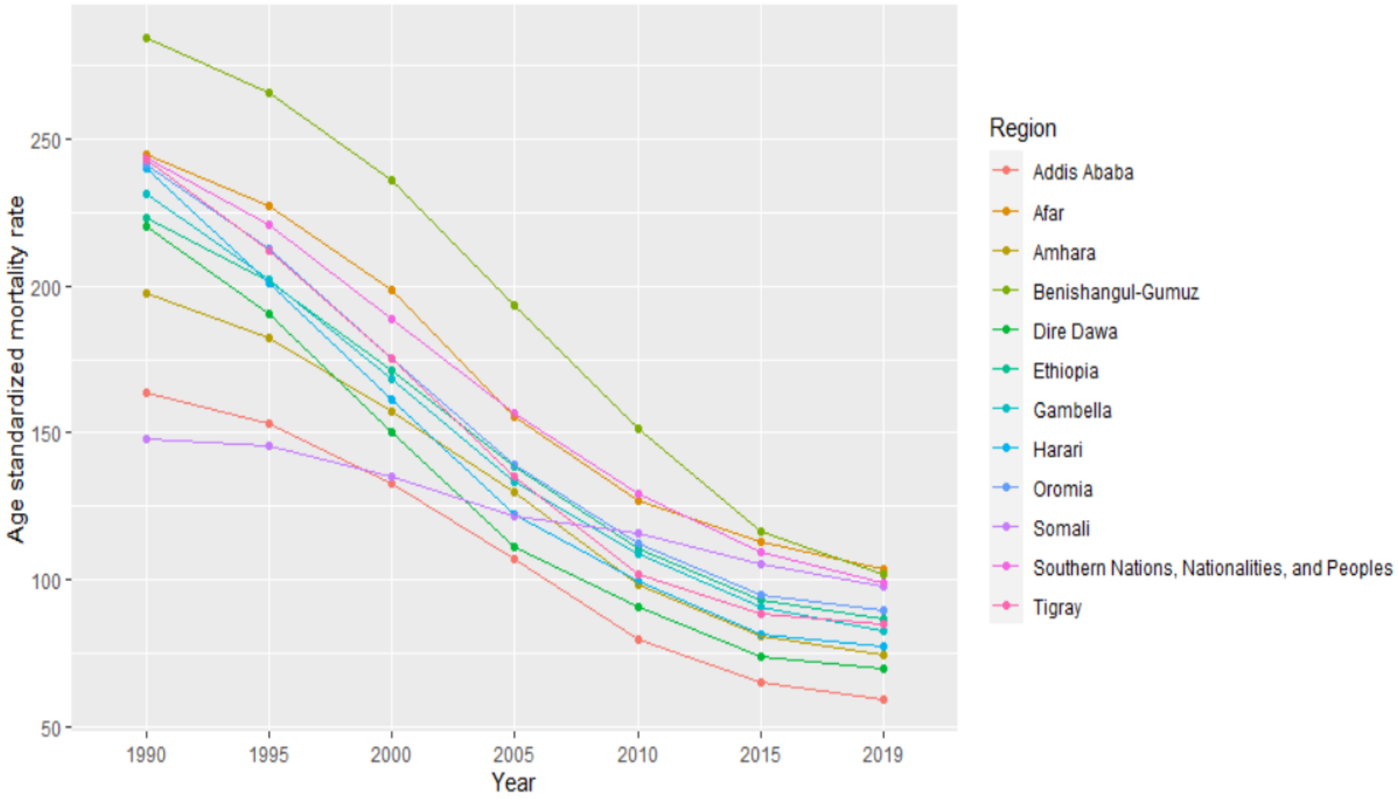


For peer review only

Figure 4: Trend in LRIs age standardized years of life lost rates per 100,000 people in Ethiopia, 1990-2019

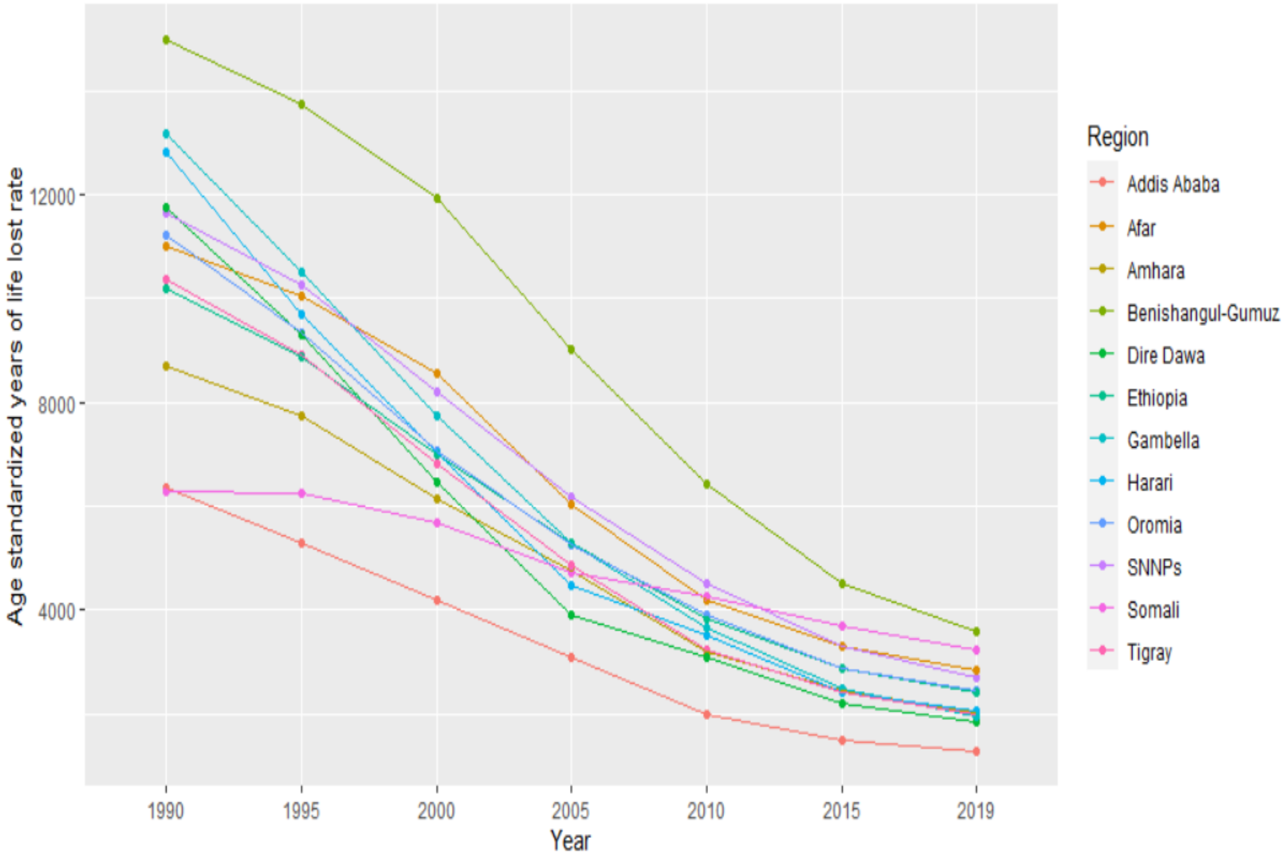


For peer review only - http://bmjopen.bmj.com/site/about/guidelines.xhtml

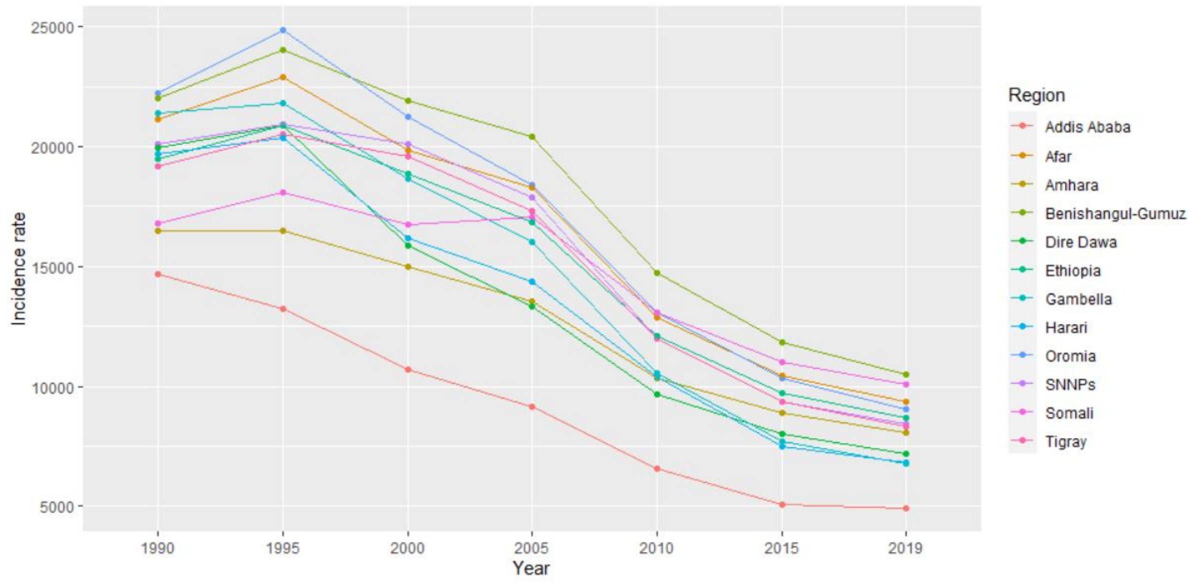


Appendix figure 1: Children under 5 years of age incidence rate per 100,000 populations due to LRI in Ethiopia, 1990- 2019

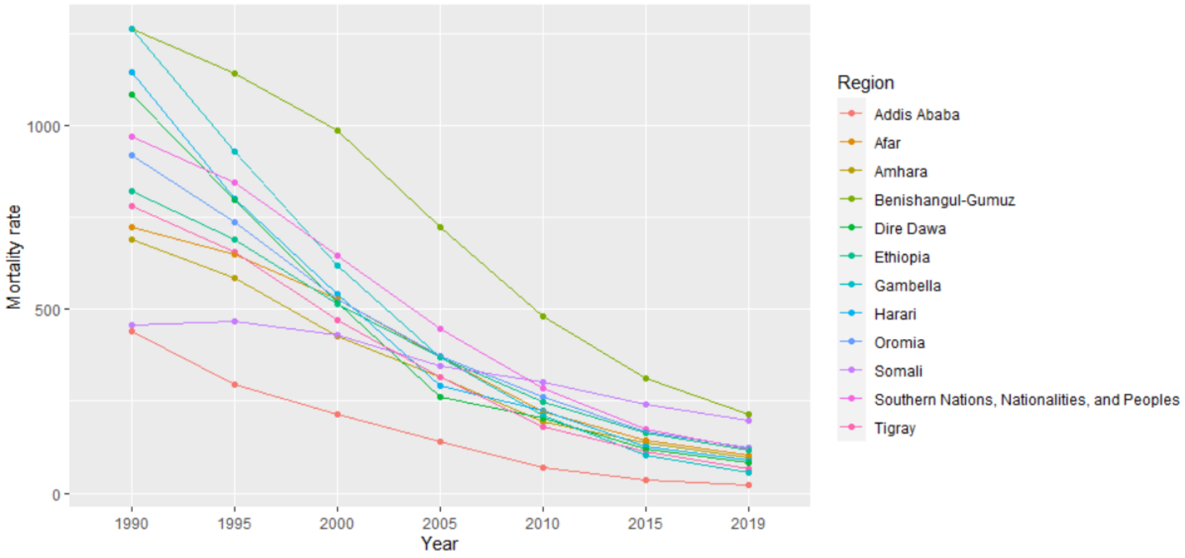


Appendix figure 2: Children younger than 5 years mortality rate per 100,000 populations for lower respiratory infections in Ethiopia, 1990-2019

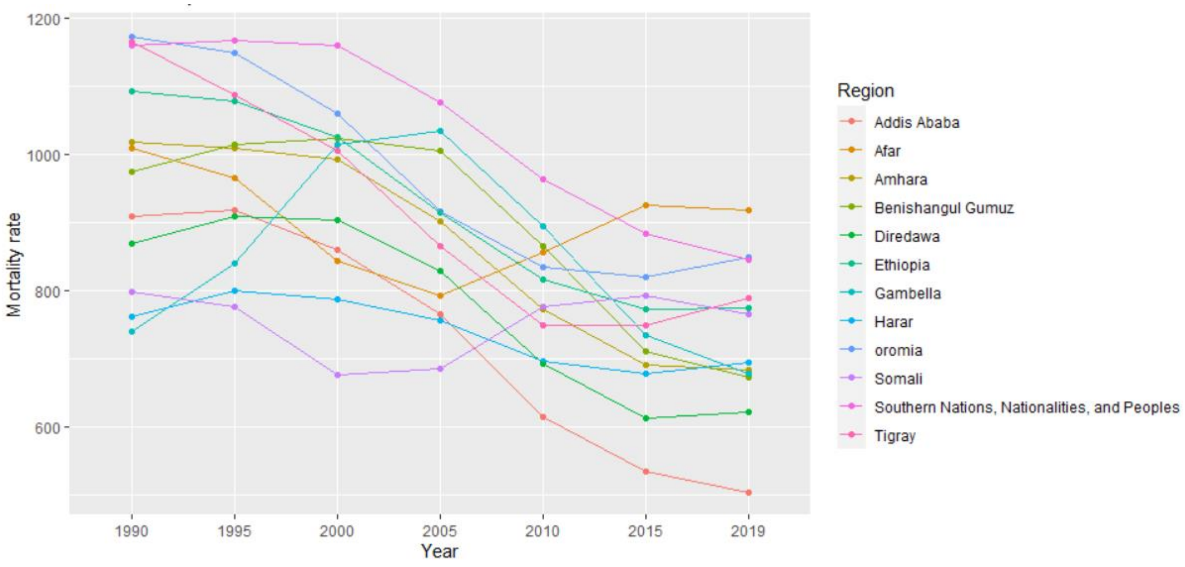


Appendix figure 3: Adults older than 70 years mortality rate per 100,000 populations for lower respiratory infections in Ethiopia, 1990-2019

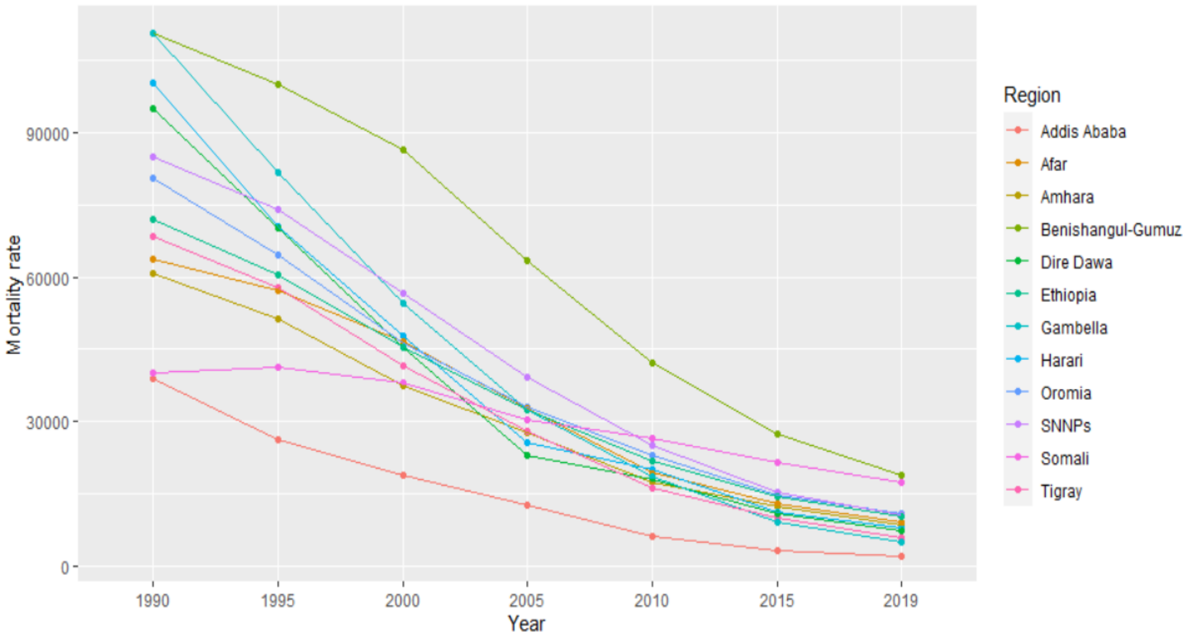


Appendix figure 4: Children younger than 5 years YLL per 100,000 populations for lower respiratory infections in Ethiopia, 1990-2019

For peer review only - http://bmjopen.bmj.com/site/about/guidelines.xhtml

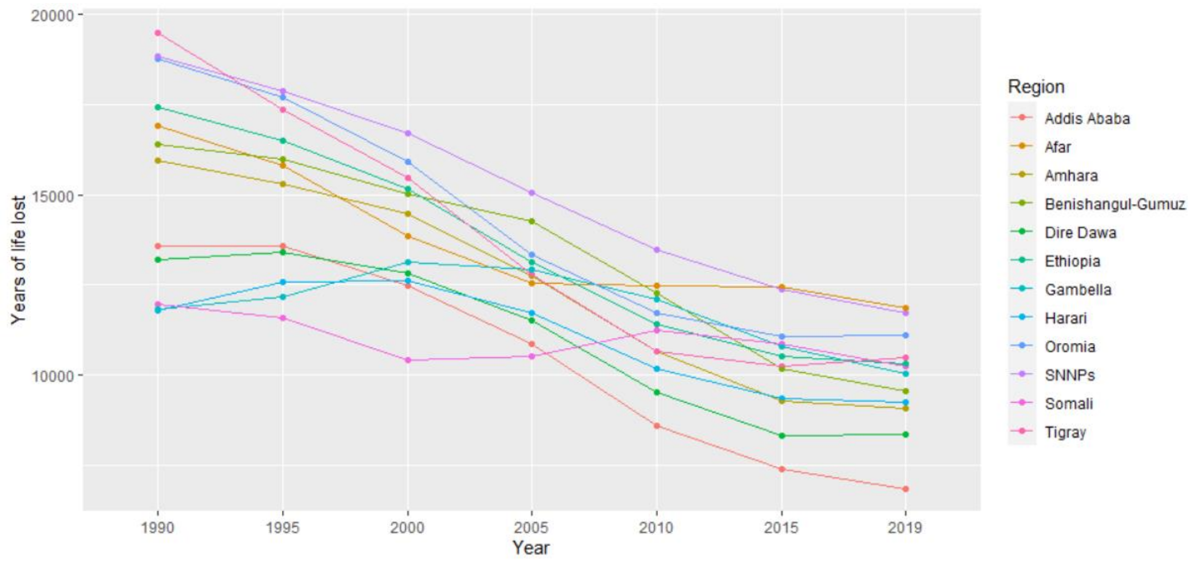


Appendix figure 5: Adults older than 70 years YLL per 100,000 populations for lower respiratory infections in Ethiopia, 1990-2019

| Location | Alcohol use | Ambient particulate matter pollution | Child stunting | Child underweight | child wasting | High temperature | Household air pollution from solid fuel- | Low birth weight | Low temperature | No access to handwashing facility | Non-exclusive breastfeeding | Secondhand smoke | Short gestation | Smoking |
|---|---|---|---|---|---|---|---|---|---|---|---|---|---|---|
| Addis Ababa | 1.4 | 4 | 0.1 | 0.1 | 0.7 | 0 | 1.7 | 0.7 | 3.7 | 4.8 | 0.1 | 0.4 | 0.7 | 1 |
| Oromia | 0.6 | 2.5 | 2.5 | 1.9 | 11.2 | 0.9 | 21.6 | 4.3 | 2.2 | 10.3 | 0.9 | 0.6 | 3.5 | 1.3 |
| Amhara | 0.4 | 2.5 | 1.8 | 1.4 | 6.7 | 0.9 | 19.8 | 3.3 | 2.4 | 9.5 | 0.4 | 0.6 | 2.6 | 0.4 |
| SNNPs | 0.8 | 2.4 | 2.7 | 2.2 | 10.2 | 1.8 | 19.9 | 3.5 | 1.4 | 9.6 | 0.8 | 0.5 | 2.9 | 0.9 |
| Tigray | 0.7 | 3.3 | 0.8 | 0.8 | 4 | 1.1 | 17.7 | 2.8 | 1.2 | 9.4 | 0.3 | 0.5 | 2.4 | 0.4 |
| Harari | 1 | 3.8 | 0.9 | 0.7 | 5.3 | 0 | 9.6 | 2.4 | 1.1 | 7.6 | 0.6 | 0.6 | 2.1 | 3.2 |
| Afar | 0.4 | 2.1 | 1.8 | 1.9 | 8.5 | 6.3 | 21 | 4.5 | 0.1 | 9.4 | 0.9 | 0.6 | 3.6 | 1.4 |
| Somali | 0.3 | 1.6 | 3.3 | 3.8 | 22.2 | 4.8 | 31.6 | 7.2 | 0.4 | 13.3 | 2.5 | 0.9 | 5.7 | 1.8 |
| Benishangul-Gumuz | 0.4 | 3.4 | 4.5 | 3.5 | 20 | 4.7 | 27.7 | 4.9 | 0.6 | 13.3 | 1.3 | 0.7 | 4 | 0.8 |
| Dire Dawa | 0.9 | 3.6 | 0.7 | 0.7 | 5.1 | 0.4 | 8.3 | 2.4 | 0.5 | 6.9 | 0.4 | 0.5 | 2 | 2.1 |
| Gambella | 0.8 | 2.2 | 0.4 | 0.4 | 3.2 | 2.5 | 9.3 | 2.2 | 0.1 | 5.4 | 0.4 | 0.3 | 1.9 | 2.1 |
| Ethiopia | 0.6 | 2.5 | 2.2 | 1.8 | 9.9 | 1.4 | 20.5 | 3.9 | 1.9 | 9.9 | 0.8 | 0.6 | 3.2 | 1 |

Appendix figure 6: Attribution of the risk factors to LRIs death rate per 100,000 population in all age groups between 1990 and 2019 for Ethiopia and its regions, both sexes, number of death, 2019. SNNPs: Southern Nations, Nationalities, and Peoples.

| | Ambient particulate matter pollution | child stunting | Child under weight | child wasting | high temperature | Household air pollution from solid fuels | Low birth weight | Low temperature | No access to handwashing facility | Non-exclusive breastfeeding | Secondhand smoke | Short gestation |
|---|---|---|---|---|---|---|---|---|---|---|---|---|
| Addis Ababa | 3.63 | 0.82 | 0.64 | 8.35 | 0.00 | 1.75 | 8.86 | 3.29 | 4.27 | 1.32 | 0.31 | 7.98 |
| Oromia | 6.41 | 14.75 | 11.45 | 67.14 | 2.34 | 60.08 | 25.95 | 6.05 | 28.32 | 5.25 | 1.66 | 21.03 |
| Amhara | 5.40 | 13.01 | 9.84 | 48.10 | 2.02 | 46.86 | 23.47 | 5.46 | 21.99 | 2.81 | 1.36 | 18.88 |
| SNNPs | 6.36 | 16.67 | 13.77 | 63.88 | 5.03 | 57.68 | 21.95 | 4.12 | 27.49 | 5.28 | 1.38 | 18.31 |
| Tigray | 5.03 | 6.15 | 5.75 | 29.88 | 1.87 | 29.89 | 20.59 | 2.00 | 15.32 | 1.91 | 0.79 | 17.45 |
| Harari | 9.12 | 7.21 | 5.80 | 43.66 | 0.01 | 25.51 | 19.63 | 2.74 | 19.30 | 5.00 | 1.62 | 17.18 |
| Afar | 4.88 | 11.42 | 11.57 | 52.76 | 15.77 | 53.12 | 28.03 | 0.26 | 23.50 | 5.49 | 1.33 | 22.31 |
| Somali | 5.40 | 18.08 | 20.92 | 121.83 | 16.76 | 110.21 | 39.76 | 1.46 | 46.07 | 13.99 | 3.18 | 31.30 |
| Benishangul-Gumuz | 12.63 | 28.22 | 22.25 | 125.72 | 17.81 | 105.51 | 30.75 | 2.22 | 50.23 | 8.05 | 2.61 | 24.89 |
| Dire Dawa | 9.38 | 5.50 | 5.64 | 42.47 | 1.11 | 23.88 | 19.72 | 1.42 | 18.88 | 3.67 | 1.50 | 16.71 |
| Gambella | 4.75 | 3.13 | 3.08 | 25.92 | 6.16 | 23.51 | 18.20 | 0.18 | 13.02 | 2.86 | 0.60 | 15.31 |
| Ethiopia | 6.06 | 14.39 | 11.93 | 64.00 | 4.28 | 58.22 | 25.08 | 4.73 | 27.20 | 5.22 | 1.58 | 20.40 |

Appendix figure 7: Attribution of the risk factors to LRIs death rate per 100,000 population in children younger than 5 years between 1990 and 2019 for Ethiopia and its regions, both sexes, number of death, 2019. SNNPs: Southern Nations, Nationalities, and Peoples.

Appendix table 1: Number and percentage changes of episode attributable to LRIs in 1990 and 2019 for Ethiopia, its regions, both sexes

| | **All ages** | | | **Children younger than 5 years** | | | **People above 70** | | |
|---|---|---|---|---|---|---|---|---|---|
| Location | Episode, (95%UI),1990 | Episode, (95%UI),2019 | Change, % | Episode, (95%UI),1990 | Episode, (95%UI),2019 | Change, % | Episode, (95%UI),1990 | Episode, (95%UI),2019 | Change,% |
| Addis Ababa | 194122.9(175403.8-213856.2) | 176027.6(162152.3-190557.8) | 10 | 44940.6(35673.3-56859.6) | 14522.3(11237-18367.5) | 68 | 8099.5(6891.6-9463.2) | 24034.2(20749.5-27702.3) | 196** |
| Oromia | 2164243.7(1957719.5-2397957.9) | 2597863.9(2372089.2-2859925.6) | 20 | 739239.6(578477-935022.8) | 613676.6(480687.2-765626.5) | 17 | 110627.2(95303.7-129579.9) | 292179.4(254670.4-342438.5) | 164** |
| Amhara | 1508431.4(1368059.8-1671550.1) | 1400682.4(1285323-1530939.8) | 8 | 423999.3(334700.8-538426.8) | 261397.4(206754.7-327889.7) | 39 | 116332.8(98774.4-137194.5) | 198977.5(173809.4-229925.5) | 71** |
| SNNPs | 1235554.9(1108317.9-1368520.3) | 1330491.5(1217176.7-1452694.1) | 7 | 421825.6(335087.1-526155.2) | 307771.3(248326.4-385053.7) | 28 | 68084.5(57450-79640.8) | 99723(88168.3-113438) | 46** |
| Tigray | 360795.1(328276.3-400714.9) | 409829.8(376290.4-445030.2) | 13 | 109047.9(86435.2-137691.1) | 69958.2(55210.7-87468.6) | 36 | 20653.3(17469.9-24256.6) | 62677.2(53933.1-72927.6) | 203** |
| Harari | 15145.9(13618.9-16851.9) | 13765.3(12657-15054.7) | 10 | 4693.6(3691.4-5990.1) | 2188.4(1723.8-2761.3) | 54 | 307.2(259.6-357) | 1614.8(1396-1863.6) | 425** |
| Afar | 113506.8(102317-126084.3) | 113210.3(102987.9-124578) | 1 | 32991.6(25672.4-42147.6) | 26177.2(20744.1-32743.3) | 21 | 2640.2(2212.5-3150.9) | 7222.5(6246.8-8559.1) | 173** |
| Somali | 300306.3(268988-336331.8) | 463220.4(420429.8-516009.4) | 54 | 103526.3(81391.3-131352.8) | 126089.6(99611.9-160423.3) | 21** | 6182.3(5316-7370.4) | 30203.2(25942.8-35038.5) | 388** |
| BG | 68501.6(61804.9-75736.4) | 71675.7(64972.1-79105.8) | 4 | 20970.5(16626.7-26454.5) | 18175.6(14339.5-22922.1) | 14 | 3288.9(2784.8-3911.4) | 4311.3(3743.8-5113.9) | 31** |
| Dire Dawa | 30607.1(27359.8-34252.2) | 26221.1(24010.1-28647.8) | 15 | 9547.4(7523-12185) | 4387.7(3439.6-5583.9) | 55 | 1274.8(1090.1-1483.5) | 2840.1(2469-3289.5) | 122** |
| Gambella | 19687.9(17723.9-21945.7) | 25685.2(23516.6-28093.2) | 30** | 6747.7(5338.7-8613.7) | 4335.4(3428.4-5400.9) | 36 | 1568.1(1316.6-1825.8) | 1489.6(1281.4-1737.3) | 6 |
| Ethiopia* | 6010904.2(5467801.6-6648644.3) | 6628673.6(6108786.2-7230986.3) | 10** | 1917530.8(1518850.1-2400978.5) | 1448680.4(1150089.8-1799704.4) | 25 | 339059.4(293491.9-387514.2) | 725273.3(640315.4-837746.3) | **113** ** |

SNNP: Southern Nations, Nationalities, and Peoples; BG: Benishangul Gumuz; *country's estimate; ***percentage increase between 1990 and 2019*

For peer review only

Appendix table 2: Rate and percentage changes of episodes attributable to LRIs in 1990 and 2019 for Ethiopia and its regions, both sexes

| | **Age standardized** | | | **Children younger than 5 years** | | | **People above70** | | |
|---|---|---|---|---|---|---|---|---|---|
| **Location** | Episode per 100,000 people (95%UI),1990 | Episode per 100,000 people (95%UI),2019 | Change,% | Episode per 100,000 people (95%UI),1990 | Episode per 100,000 people (95%UI),2019 | Change,% | Episode per 100,000 people (95%UI),1990 | Episode per 100,000 people (95%UI),2019 | Change,% |
| Addis Ababa | 11373(10484.2-12197.4) | 6788.1(6285.1-7339.1) | 40 | 14666.9(11642.4-18556.9) | 4927.2(3812.5-6231.8) | 67 | 35138(29897.7-41054.2) | 30748.4(26546-35441.2) | 13 |
| Oromia | 14613.1(13525.9-15725.8) | 8659.9(8040.1-9411.6) | 41 | 22232(17397.2-28120) | 9039.4(7080.5-11277.7) | 60 | 46498.3(40057.6-54464.4) | 41564.6(36228.7-48714.4) | 11 |
| Amhara | 12826.4(11830-13882.8) | 7716.9(7162.3-8321.3) | 40 | 16487.1(13014.7-20936.6) | 8061.7(6376.4-10112.3) | 52 | 43580(37002.4-51395.2) | 35377.7(30902.9-40880.2) | 19 |
| SNNPs | 13591.9(12538.6-14646.8) | 8235.5(7670.5-8846.4) | 39 | 20106.4(15972-25079.3) | 8427.1(6799.4-10543.2) | 59 | 44628.1(37657.5-52203.1) | 36616.1(32373.5-41652) | 18 |
| Tigray | 13927.2(12858.2-15100.7) | 8663.8(8034-9382.3) | 38 | 19185.4(15207-24224.8) | 8342.1(6583.5-10430.1) | 57 | 43748.4(37005.1-51381) | 40859(35158.8-47541.2) | 7 |
| Harari | 13014.6(11941-14066.7) | 7190.5(6684.1-7718.6) | 45 | 19672.6(15471.9-25106.9) | 6821.6(5373.5-8607.3) | 66 | 33916.7(28665.1-39416.7) | 32797.4(28354-37850.7) | 4 |
| Afar | 15245.7(14050.8-16525.6) | 9350.1(8648.8-10157.4) | 39 | 21151.6(16459.1-27021.7) | 9365.5(7421.7-11714.7) | 56 | 39849(33393.8-47557.2) | 44368.7(38375.2-52580.1) | 11** |
| Somali | 11482.7(10638.1-12368.7) | 9220(8515.8-10046.3) | 20 | 16792.9(13202.4-21306.6) | 10062.7(7949.6-12802.8) | 41 | 32748.3(28159.7-39042.1) | 41031.2(35243.4-47600) | 25** |
| BG | 15628.1(14481-16809.3) | 9054.6(8394.2-9766) | 42 | 22041.1(17475.5-27805) | 10481.8(8269.5-13219) | 53 | 42911.6(36334.5-51032.5) | 37592.7(32643.9-44591.2) | 13 |
| Dire Dawa | 12806.6(11807.9-13871.4) | 7148.8(6634.6-7750.7) | 44 | 19968.2(15734.3-25484.7) | 7191.5(5637.6-9152.1) | 64 | 38333(32779.4-44607) | 32033.6(27847.4-37101.4) | 17 |
| Gambella | 13623.1(12637.4-14572.4) | 7575.8(7002.3-8128.2) | 44 | 21419.8(16947.2-27343.2) | 6791.1(5370.4-8460.1) | 69 | 40197.8(33751.8-46803.7) | 32657.1(28093.9-38087.2) | 19 |
| Ethiopia* | 13619.7(12640.8-14588.6) | 8313.7(7757.6-8918) | 39 | 19486.4(15434.9-24399.3) | 8685.3(6895.1-10789.8) | 56 | 44092.2(38166.5-50393.4) | 38394.4(33896.9-44348.5) | 13 |

SNNP: Southern Nations, Nationalities, and Peoples; BG: Benishangul Gumuz; *country's estimate*; **percentage increase between 2019 and 1990*

For peer review only

Appendix table 3: Number and percentage changes of death attributable to LRIs in 1990 and 2019 for Ethiopia and its regions, both sexes.

| | All age | | | Children younger than 5 | | | People above 70 years | | |
|---|---|---|---|---|---|---|---|---|---|
| **Location** | death, (95%UI),1990 | death, (95%UI),2019 | Change,% | death, (95%UI), 1990 | Death, (95%UI),2019 | Change, % | Death , (95%UI),1990 | Death, (95%UI),2019 | Change,% |
| Addis Ababa | 2159.3(1731.9-2690.7) | 918.8(767.4-1116.9) | 58 | 1353(981.9-1846.7) | 69.1(41.7-107.4) | 95 | 209.5(154.3-291.5) | 393.6(320.9-495.3) | 87** |
| Oromia | 40004.4(29432.4-51942.2) | 18206.1(15193.3-21745.4) | 55 | 30614.3(20932.5-42033.5) | 8306.2(5763.5-11611.8) | 73 | 2793.5(2005.1-3673) | 5971.6(4831.7-7093.4) | 113** |
| Amhara | 25449.3(20869.5-30724.8) | 9525.5(7530.5-11872.2) | 63 | 17780.1(13674.3-22720.1) | 3079.5(1805.3-4737.3) | 83 | 2717.2(2016.4-3535.8) | 3839.9(3022.6-4825.6) | 41** |
| SNNPs | 26044.7(20108-33017) | 9494.7(7713.5-11649) | -64 | 20390.6(14627.6-26712.9) | 4326.6(2891.3-6157.2) | 79 | 1771.7(1254.5-2417.6) | 2302.6(1867.3-2772.8) | 29** |
| Tigray | 6463.8(5272.1-7762.9) | 2551.4(2090.3-3028.6) | 61 | 4436(3373.5-5736) | 564.2(364.9-825) | 88 | 550.8(395.7-749.1) | 1209.7(978.5-1467.8) | 119** |
| Harari | 336(233.3-448.8) | 93.1(73.8-116) | 73 | 273.5(174.1-382.7) | 28.8(16.4-44.3) | 90 | 6.9(3.7-11) | 34.1(27.1-41.8) | 395** |
| Afar | 1717.5(1300.7-2243.4) | 706.2(567.7-869.6) | -59 | 1128.9(751.1-1596.7) | 284.2(182.5-426) | 75 | 66.9(43.5-102.1) | 149.3(117.3-186.6) | 123** |
| Somali | 3603.8(2620-4713.6) | 3907.5(3007.4-4959.4) | #N/A | 2811.1(1929.6-3874.8) | 2472.2(1695.2-3506.8) | 13 | 150.8(98-222.3) | 563.1(433.4-715.8) | 273** |
| BG | 1549.2(1131.1-2097) | 619(470.7-803) | 61 | 1204.8(805.6-1703.9) | 373(245.3-539.3) | 70 | 74.7(50.3-105.7) | 77(61.1-97.3) | 3** |
| Dire Dawa | 632.3(445.5-830.9) | 154.6(120.6-193.8) | 76 | 518.4(329.5-718.7) | 51(27.3-82.9) | 91 | 28.8(20.1-40) | 55(44-67.9) | 90** |
| Gambella | 475.9(328.5-635.7) | 123.2(98.2-151.9) | 75 | 398.6(253.7-555.7) | 36.4(20.7-57.2) | 91 | 28.8(18.6-41.9) | 30.9(24.6-38.1) | 7** |
| Ethiopia* | 108436.6(87669-132758.3) | 46300.7(39515.8-54642.2) | 58 | 80909.9(62491.6-103448.8) | 19591.8(14018.4-26899) | 76 | 8400.1(6490.3-10700.9) | 14627.6(12393.7-16892.8) | 74** |

SNNP: Southern Nations, Nationalities, and Peoples; BG: Benishangul Gumuz; *country's estimate; ***percentage increase between 2019 and 1990*

For peer review only - http://bmjopen.bmj.com/site/about/guidelines.xhtml

Appendix table 4: Age standardized mortality rate, Ethiopia and its regions, by sex, 2019.

| Age standardized mortality rate per 100,000 people, 2019 | | |
|---|---|---|
| **Location** | Female | Male |
| Addis Ababa | 47(35.5-62.5) | 73.6(55-98.3) |
| Oromia | 77.3(62-92.4) | 100.3(79.9-123.5) |
| Amhara | 57.9(44.1-73.7) | 92.5(70.5-122.1) |
| SNNPs | 79.2(63.5-95.2) | 118.8(94.3-146.3) |
| Tigray | 73.2(55.9-93.5) | 96.9(74.8-122.3) |
| Harari | 63(49-79.9) | 95.7(72.5-123.4) |
| Afar | 112.4(87.3-142.3) | 98.7(76.9-127.3) |
| Somali | 88.3(69-111.4) | 101.7(77.2-130.7) |
| Benishangul-Gumuz | 109.2(82.6-138.8) | 95.8(74.6-124.1) |
| Dire Dawa | 56.2(42.5-71.3) | 85.4(67.3-109.5) |
| Gambella | 47.9(34.6-60.1) | 120.2(95.6-149.5) |
| Ethiopia | 71.8(60.2-82.9) | 100.6(84-121.4) |

SNNP: Southern Nations, Nationalities, and Peoples

Appendix table 5: Number and percentage changes of YLL attributable to LRIs in 1990 and 2019 for Ethiopia and its regions, both sexes

| | **all age** | | | **Children younger than 5 years** | | | **People above 70** | | |
|---|---|---|---|---|---|---|---|---|---|
| | YLL, (95%UI),1990 | YLL, (95%UI),2019 | Change,% | YLL, (95%UI),1990 | YLL, (95%UI),2019 | Change,% | YLL, (95%UI),1990 | YLL, (95%UI),2019 | Change,% |
| Addis Ababa | 149543.9(115542.6-195437) | 29703.1(23833.4-36855.7) | 81 | 118961.7(86499.1-162678.7) | 6098.2(3693.1-9483.3) | 95 | 3131.8(2301.7-4374.6) | 5359.8(4334.8-6816.8) | 71** |
| Oromia | 3019031.9(2144916.2-4057496.3) | 981808.4(748459.2-1278135) | 68 | 2680397.2(1832260.4-3680767.1) | 729313.8(506226.4-1021016.9) | 73 | 44619.5(31636-59105.6) | 78166.1(62837.5-93527.9) | 75** |
| Amhara | 1813522.3(1445106.4-2246142.2) | 428277.8(309674.7-583000.2) | 77 | 1560078.3(1201305.4-1993362.8) | 270909.2(158846-416496.1) | 83 | 42578.2(31294.7-56216.9) | 50955.1(39726.1-64844.6) | 19** |
| SNNPs | 1993643.3(1489598.4-2577052.3) | 543135.8(405565.6-711397.4) | 73 | 1785675.1(1284021-2339272.5) | 380166.4(254486.8-541224.9) | 79 | 28746.2(20086.2-39789.1) | 31964.4(25673.6-38778.5) | 11** |

| | | | | | | | | | |
|---|---|---|---|---|---|---|---|---|---|
| Tigray | 455613.4(364406.1-569702.1) | 96429.9(74139.8-122350.5) | 79 | 389399.3(296618.1-504230.2) | 49712.6(32198.3-72764.9) | 88 | 9207.1(6568.7-12629.1) | 16086.6(12946.2-19581.8) | 74** |
| Harari | 26755.3(17866.5-36380.3) | 4273.6(2999.1-5798.4) | 85 | 23959.3(15245.4-33513.8) | 2541.6(1449.6-3887.3) | 90 | 106.7(57.6-175.1) | 455.9(361.3-558.3) | 327** |
| Afar | 124052(89386.4-166880.5) | 39507.6(29535.5-52178.5) | 69 | 99155.3(65972.9-140018.8) | 24998.1(16028.5-37426.3) | 75 | 1121.4(723.7-1722.7) | 1931.7(1521.3-2395.6) | 72** |
| Somali | 281532.2(201275.3-374880.8) | 266379.4(193669.7-357880.7) | 6 | 247138.5(169803.8-339740.3) | 216954(148995.2-307369.5) | 13 | 2256.6(1434.6-3376.4) | 7542.1(5740-9680.2) | 234** |
| BG | 119211.2(83517.3-165095.7) | 42245.6(30388.8-57591.8) | -65 | 105463.5(70756-148983.1) | 32718.1(21576.4-47307.2) | 69 | 1256.7(836.7-1780.3) | 1094.3(857.5-1389.1) | 13 |
| Dire Dawa | 50024.5(33464.5-67580.6) | 7267.7(5038-10264.7) | 86 | 45377.4(28907.2-62914.9) | 4497.4(2411.8-7298.3) | 91 | 439.2(306.6-609.5) | 742.1(586.6-922.8) | 68** |
| Gambella | 37680.8(24783.6-51516.1) | 6064.4(4389.3-7853.6) | 84 | 34899.5(22233.1-48609.4) | 3212.2(1830.4-5034.1) | 91 | 460.7(299.3-663.6) | 457.5(352.6-577.6) | 1 |
| Ethiopia* | 8070611.3(6356905.2-10091563.7) | 2445093.7(1934420.8-3119838.6) | 70 | 7090505.4(5482895.7-9059421.2) | 1721122.3(1231032.1-2362958.7) | 76 | 133924.6(102756.8-170932.9) | 194756.2(165462-225502.1) | 45** |

SNNP: Southern Nations, Nationalities, and Peoples; BG: Benishangul Gumuz; *country's estimate; ***percentage increase between 2019 and 1990*

Appendix table 6: Rate and percentage changes of YLL attributable to LRIs in 1990 and 2019 for Ethiopia and its regions, both sexes.

| | **all age** | | | **Children younger than 5 years** | | | **People above 70** | | |
|---|---|---|---|---|---|---|---|---|---|
| **Location** | YLL per 100,000 people,1990 | YLL per 100,000 people, 2019 | Change,% | YLL per 100,000 people,1990 | YLL per 100,000 people, 2019 | Change,% | YLL per 100,000 people,1990 | YLL per 100,000 people, 2019 | Change,% |
| Addis Ababa | 6362.2(5161.9-7847.9) | 1285.6(1065-1561.8) | 80 | 38824.7(28230.1-53092.4) | 6857.1(5545.8-8721.2) | 95 | 13587(9985.4-18978.3) | 6857.1(5545.8-8721.2) | 50 |
| Oromia | 11217.6(8517-14318) | 2433.8(2042.2-2879.5) | 78 | 80610.7(55103.7-110696) | 11119.7(8939.1-13305) | 87 | 18754.2(13297.1-24843) | 11119.7(8939.1-13305) | 41 |
| Amhara | 8690.4(7146.3-10453.8) | 2016.4(1551.4-2541.8) | 77 | 60663.3(46712.5-77511.5) | 9059.7(7063.2-11529.2) | 87 | 15950.4(11723.4-21059.7) | 9059.7(7063.2-11529.2) | 44 |
| SNNPs | 11633(9119.4-14477.9) | 2698.1(2243.5-3250.2) | 77 | 85114.7(61203.2-111502.1) | 11736.6(9426.7-14238.6) | 88 | 18842.6(13166.1-26081) | 11736.6(9426.7-14238.6) | 38 |
| Tigray | 10346.4(8522.9-12340.3) | 1977.1(1593.9-2395) | 81 | 68509.3(52185.7-88712.2) | 10486.8(8439.5-12765.3) | 92 | 19502.8(13914.1-26751.2) | 10486.8(8439.5-12765.3) | 47 |
| Harari | 12826.5(9336.5-16985.4) | 2060.4(1595.6-2623.1) | 84 | 100421.7(63898.7-140467.8) | 9260.4(7338.7-11340.3) | 93 | 11782.6(6360.4-19341.1) | 9260.4(7338.7-11340.3) | 22 |

| | | | | | | | | | |
|---|---|---|---|---|---|---|---|---|---|
| Afar | 10993.5(8520.8-14193.6) | 2824.6(2323-3414) | 74 | 63570.5(42296.6-89769) | 11867.1(9346-14716.9) | 86 | 16925.5(10923.7-26001.7) | 11867.1(9346-14716.9) | 30 |
| Somali | 6286.9(4860.5-7970) | 3236.4(2537.4-4006) | 49 | 40088.1(27543.7-55109) | 10246(7797.8-13150.6) | 57 | 11953.6(7599.2-17885.5) | 10246(7797.8-13150.6) | 15 |
| BG | 14965.1(11197.4-19998.6) | 3571.1(2772.4-4510.7) | 76 | 110847.2(74368-156588.4) | 9541.9(7476.9-12112.3) | 83 | 16397.3(10917-23228.5) | 9541.9(7476.9-12112.3) | 42 |
| Dire Dawa | 11749.7(8605.4-15249.5) | 1832.8(1407.3-2363.1) | 84 | 94905.3(60458.5-131584.5) | 8371(6616.3-10408.2) | 93 | 13207.9(9219.6-18328.2) | 8371(6616.3-10408.2) | 37 |
| Gambella | 13175.8(9291-17411) | 1937.4(1567.6-2344.6) | 85 | 110783.8(70576-154304.1) | 10030.9(7730.2-12664.9) | 96 | 11811.4(7673.5-17012.5) | 10030.9(7730.2-12664.9) | 16 |
| Ethiopia* | 10189.1(8347.5-12201.8) | 2404.5(2059.4-2833.3) | 76 | 72055.4(55718.5-92064) | 10309.9(8759.2-11937.6) | 86 | 17415.9(13362.7-22228.5) | 10309.9(8759.2-11937.6) | 41 |

SNNP: Southern Nations, Nationalities, and Peoples; BG: Benishangul Gumuz; *country's estimate; ***percentage increase between 2019 and 1990*

For peer review only

## Appendix: Authors

### Providing data or critical feedback on data sources

Semagn Mekonnen Abate, Mesafint Molla Adane, Addis Aklilu, Dejene Tsegaye Alem, Mulusew A Asemahagn, Hunegnaw Abebe, Melaku Ashagrie Belete, Tekleberhan Hailemariam, Tezera Moshago Berheto, Belay Boda Abule Bodicha, Daniel Baza Gargamo, Alemayehu Hailu, Awoke Misganaw, Mohsen Naghavi, Negussie Boti Sidamo, Yonatan Solomon, Shambel Wedajo, Melat Weldemariam, Amanuel Yigezu, Fentabil Getnet, and Yazachew Yismaw.

### Developing methods or computational machinery

Semagn Mekonnen Abate, Tezera Moshago Berheto, Alemayehu Hailu, Mohsen Naghavi, Negussie Boti Sidamo, and Amanuel Yigezu.

### Providing critical feedback on methods or results

Semagn Mekonnen Abate, Mesafint Molla Adane, Gizachew Taddesse Akalu, Addis Aklilu, Dejene Tsegaye Alem, Zeleke Gebru, Mulusew Andualem Asemahagn, Daniel Atlaw, Tewachew Awoke, Hunegnaw Abebe, Melaku Ashagrie Belete, Tekleberhan Hailemariam, Tezera Moshago Berheto, Alemeshet Yirga, Setognal Birara Aychiluhm, Belay Boda Abule Bodicha, Chuchu Churko, Feleke Mekonnen Demeke, Abebaw Alemayehu Desta, Lankamo Ena, Tahir Eyayu, Zinabu Fentaw, Daniel Baza Gargamo, Mesfin Damtew Gebrehiwot, Mathewos Alemu Gebremichael, Melaku Getachew, Ababi Zergaw, Alemayehu Hailu, Getahun Molla, Awoke Misganaw, Mohsen Naghavi, Biniyam Sahiledengle, Bereket Beyene, Migbar Sibhat, Negussie Boti Sidamo, Damtew Damtew Solomon, Yonatan Solomon, Birhanu Wagaye, Shambel Wedajo, Melat Weldemariam, Amanuel Yigezu, Fentabil Getnet, and Yazachew Yismaw.

### Drafting the work or revising is critically for important intellectual content

Semagn Mekonnen Abate, Gizachew Taddesse Akalu, Mulusew A Asemahagn, Daniel Atlaw, Niguss Cherie Bekele, Melaku Ashagrie Belete, Tezera Moshago Berheto, Setognal Birara Aychiluhm, Belay Boda Abule Bodicha, Chuchu Churko, Tahir Eyayu, Zinabu Fentaw, Daniel Baza Gargamo, Melaku Getachew, Ababi Zergaw, Firehiwot Abebe Gobena, Muluken Argaw Haile, Alemayehu Hailu, Solomon Tessema Memirie, Awoke Misganaw, Mohsen Naghavi, Biniyam Sahiledengle, Bereket Beyene, Negussie Boti Sidamo, Yonatan Solomon, Dereje Mengistu Tolosa, Birhanu Wagaye, Ally Walker, Amanuel Yigezu, and Fentabil Getnet.

### Managing the estimation or publications process

Semagn Mekonnen Abate, Awoke Misganaw, Mohsen Naghavi, Negussie Boti Sidamo, and Amanuel Yigezu.

For peer review only

BMJ Open

# The burden of lower respiratory infections and associated risk factors across regions in Ethiopia: A subnational analysis of the Global Burden of Diseases 2019 Study

| | |
|---|---|
| Journal: | *BMJ Open* |
| Manuscript ID | bmjopen-2022-068498.R1 |
| Article Type: | Original research |
| Date Submitted by the Author: | 26-Jun-2023 |
| Complete List of Authors: | Yigezu, Amanuel; Ethiopian Public Health Institute<br>Misganaw, Awoke; University of Washington, Department of Health Metrics Sciences; Ethiopian Public Health Association,<br>Getnet, Fentabil; Jigjiga University; Ethiopian Public Health Institute<br>Berheto, Tezera; Ethiopian Public Health Institute<br>Walker, Ally ; University of Washington, Department of Health Metrics Sciences<br>Zergaw, Ababi; Ethiopian Public Health Institute; Addis Ababa University College of Health Sciences, Department of Health Systems and Policy<br>Gobena, Firehiwot ; Ethiopian Health Insurance Service<br>Haile, Muluken ; Ethiopian Health Insurance Service<br>Hailu, Alemayehu; University of Bergen, Department of Global Public Health and Primary Care Medicine<br>Memirie, Solomon Tessema; Addis Ababa University, Addis Center for Ethics and Priority Setting; Harvard University, Harvard T.H. Chan School of Public Health<br>Tolosa, Dereje ; Ethiopian Health Insurance Service<br>Abate, Semagn Mekonnen; Dilla University College of Health Sciences, Department of Anesthesiology<br>Molla Adane, Mesafint; Bahir Dar University College of Medical and Health Sciences<br>Akalu, Gizachew ; St Paul's Hospital Millennium Medical College; Addis Ababa University College of Health Sciences<br>Aklilu, Addis ; Arba Minch University<br>Tsegaye, Dejen; Debre Markos University College of Health Science, Adult health nursing;<br>Gebru, Zeleke; Arba Minch University, public health; Arba minch university<br>Asemahagn, Mulusew; Bahir Dar University College of Medical and Health Sciences, School of Public Health;<br>Atlaw, Daniel; Madda Walabu University, ;<br>Awoke, Tewachew ; Bahir Dar University<br>Abebe, Hunegnaw ; Wollo University, public health<br>Belete, Melaku; Wollo University<br>Hailemariam, Tekleberhan; Mekelle University<br>Yirga, Alemeshet; Bahir Dar University, Department of pharmacy<br>Birara, Setognal; Samara University, Public Health<br>Bodicha, Belay ; Arba Minch University<br>Churko, Chuchu ; Arba Minch University, Public health |

| | |
|---|---|
| | Demeke, Feleke ; Bahir Dar University College of Medical and Health Sciences<br>Desta, Abebaw ; University of Gondar<br>Ena, Lankamo; Arba Minch University,<br>Eyayu, Tahir; Debre Tabor University, Department of Medical Laboratory Sciences<br>Fentaw, Zinabu; Wollo University, Department of Epidemiology and Biostatistics; Wollo University, Epidemiology and Biostatistics<br>Gargamo, Daniel ; Wolaita Sodo University<br>Gebrehiwot, Mesfin ; Wollo University<br>Gebremichael, Mathewos ; Arba Minch University<br>Getachew, Melaku ; Haramaya University College of Health and Medical Sciences<br>Molla, Getahun ; University of Gondar, University of Gondar Hospital<br>Sahiledengle, Biniyam; Madda Walabu University, Public Health<br>Beyene, Bereket; Arba Minch University<br>Sibhat, Migbar; Dilla University College of Health Sciences, Nursing;<br>Sidamo, Negussie ; Arba Minch University, Public Health<br>Solomon, Damtew ; Madda Walabu University<br>Solomon, Yonatan ; Dire Dawa University<br>Wagaye, Birhanu; Wollo University; Ethiopian Public Health Institute<br>Wedajo, Shambel; Wollo University,<br>Weldemariam, Melat; Arba Minch University, Department of Medical Laboratory Sciences<br>Yismaw, Yazachew ; Bahir Dar University<br>Naghavi, Moshen; University of Washington School of Public Health, |
| <b>Primary Subject Heading</b>: | Infectious diseases |
| Secondary Subject Heading: | Epidemiology, Respiratory medicine, Public health, Health policy, Global health |
| Keywords: | Epidemiology < INFECTIOUS DISEASES, Respiratory infections < THORACIC MEDICINE, Health policy < HEALTH SERVICES ADMINISTRATION & MANAGEMENT |
| | |

SCHOLARONE™
Manuscripts

*I, the Submitting Author has the right to grant and does grant on behalf of all authors of the Work (as defined in the below author licence), an exclusive licence and/or a non-exclusive licence for contributions from authors who are: i) UK Crown employees; ii) where BMJ has agreed a CC-BY licence shall apply, and/or iii) in accordance with the terms applicable for US Federal Government officers or employees acting as part of their official duties; on a worldwide, perpetual, irrevocable, royalty-free basis to BMJ Publishing Group Ltd ("BMJ") its licensees and where the relevant Journal is co-owned by BMJ to the co-owners of the Journal, to publish the Work in this journal and any other BMJ products and to exploit all rights, as set out in our licence.*

*The Submitting Author accepts and understands that any supply made under these terms is made by BMJ to the Submitting Author unless you are acting as an employee on behalf of your employer or a postgraduate student of an affiliated institution which is paying any applicable article publishing charge ("APC") for Open Access articles. Where the Submitting Author wishes to make the Work available on an Open Access basis (and intends to pay the relevant APC), the terms of reuse of such Open Access shall be governed by a Creative Commons licence – details of these licences and which Creative Commons licence will apply to this Work are set out in our licence referred to above.*

*Other than as permitted in any relevant BMJ Author's Self Archiving Policies, I confirm this Work has not been accepted for publication elsewhere, is not being considered for publication elsewhere and does not duplicate material already published. I confirm all authors consent to publication of this Work and authorise the granting of this licence.*

For peer review only

# The burden of lower respiratory infections and associated risk factors across regions in Ethiopia: A subnational analysis of the Global Burden of Diseases 2019 Study

Ethiopia Subnational-Level Lower Respiratory Infections Burden collaborators

Amanuel Yigezu*,[1] Awoke Misganaw*,[2,1] Fentabil Getnet,[1,3] Tezera Moshago Berheto,[4] Ally Walker,[5] Ababi Zergaw,[6,1] Firehiwot Abebe Gobena,[7] Muluken Argaw Haile,[8] Alemayehu Hailu,[9] Solomon Tessema Memirie,[10] Dereje Mengistu Tolosa,[11] Semagn Mekonnen Abate,[12] Mesafint Molla Adane,[13] Gizachew Taddesse Akalu,[14,15] Addis Aklilu,[16] Dejene Tsegaye Alem,[17] Zeleke Gebru,[18] Mulusew Andualem Asemahagn,[19] Daniel Atlaw,[20] Tewachew Awoke,[21] Hunegnaw Abebe[22], Melaku Ashagrie Belete,[23] Tekleberhan Hailemariam,[24] Alemeshet Yirga,[25] Setognal Birara Aychiluhm,[26] Belay Boda Abule Bodicha,[27] Chuchu Churko,[18] Feleke Mekonnen Demeke,[21] Abebaw Alemayehu Desta,[28] Lankamo Ena,[29] Tahir Eyayu,[30] Zinabu Fentaw,[31] Daniel Baza Gargamo,[32] Mesfin Damtew Gebrehiwot,[33] Mathewos Alemu Gebremichael,[34] Melaku Getachew,[35] Getahun Molla,[36] Biniyam Sahiledengle,[37] Bereket Beyene,[38] Migbar Sibhat,[39] Negussie Boti Sidamo,[18] Damtew Solomon,[40] Yonatan Solomon,[41] Birhanu Wagaye,[42,43] Shambel Wedajo,[22] Melat Weldemariam,[16] Yazachew Yismaw,[44,45] Mohsen Naghavi.[5,2]

*Joint first authors

## Affiliations

[1]National Data Management Center for Health (NDMC), Ethiopian Public Health Institute, Addis Ababa, Ethiopia; [2]Department of Health Metrics Sciences, School of Medicine, University of Washington, Seattle, WA, USA; [3]Department of Epidemiology, Jigjiga University, Jigjiga, Ethiopia; [4]HIV and TB Research Directorate, Ethiopian Public Health Institute, Addis Ababa, Ethiopia; [5]Institute for Health Metrics and Evaluation, University of Washington, Seattle, WA, USA; [6]Department of Health Systems and Policy, Addis Ababa University, Addis Ababa, Ethiopia; [7] Ethiopian Health Insurance Service, Addis Ababa, Ethiopia; [8]Program Section, Ethiopian Health Insurance Service, Addis Ababa, Ethiopia; [9]Department of Global Public Health and Primary Care, University of Bergen, Bergen, Norway; [10]Addis Center for Ethics and Priority Setting, Addis Ababa University, Addis Ababa, Ethiopia; [11]Ethiopian Health Insurance Service, Ministry of Health, Addis Ababa, Ethiopia; [12]Department of Anesthesiology, Dilla University, Addis Ababa, Ethiopia; [13]College of Medicine and Health Sciences, Bahir Dar University, Bahir Dar, Ethiopia; [14]Department of Microbiology, Immunology and Parasitology, St. Paul's Hospital Millennium Medical College, Addis Ababa, Ethiopia; [15]Department of Microbial, Cellular and Molecular Biology, Addis Ababa University, Addis Ababa, Ethiopia; [16]Department of Medical Laboratory Sciences, Arba Minch University, Arba Minch, Ethiopia; [17]Department of Nursing, Debre Markos University, Debremarkos, Ethiopia; [18]School of Public Health, Arba Minch University, Arba Minch, Ethiopia; [19]School of Public Health, Bahir Dar University, Bahir Dar, Ethiopia; [20]Department of Biomedical Science, Madda Walabu University, Bale Robe, Ethiopia; [21]Department of Medical Laboratory Sciences, Bahir Dar University, Bahir Dar, Ethiopia; [22]Department of Public Health, Wollo University, Dessie,



For peer review only

For peer review only - http://bmjopen.bmj.com/site/about/guidelines.xhtml

Ethiopia; [23]Department of Medical Laboratory Sciences, Wollo University, Dessie, Ethiopia; [24]Department of Medical Physiology, Mekelle University, Mekelle, Ethiopia; [25]School of Health Science, Bahir Dar University, Bahir Dar, Ethiopia; [26]Department of Public Health, Samara University, Samara, Ethiopia; [27]Department of Biomedical Sciences , Arba Minch University, Arba Minch, Ethiopia; [28]Department of Surgical Nursing, University of Gondar, Gondar, Ethiopia; [29]Department of Comprehensive Nursing, Arba Minch University, Arba Minch, Ethiopia; [30]Department of Medical Laboratory Sciences, Debre Tabor University, Debre Tabor, Ethiopia; [31]Department of Epidemiology and Biostatistics, Wollo University, Dessie, Ethiopia; [32]Department of Pediatrics and Neonatal Nursing, Wolaita Sodo University, Wolaita Sodo, Ethiopia; [33]Department of Environmental Health, Wollo University, Dessie, Ethiopia; [34]Department of Epidemiology and Biostatistics, Arba Minch University, Arba Minch, Ethiopia; [35]Department of Emergency and Critical Care Medicine, Haramaya University, Harar, Ethiopia; [36]Department of Epidemiology and Biostatistics, University of Gondar, Gondar, Ethiopia; [37]Department of Public Health, Madda Walabu University, Bale Robe, Ethiopia; [38]Department of Nursing, Arba Minch University, Arba Minch, Ethiopia; [39]Department of Pediatrics and Child Health Nursing, Dilla University, Dilla, Ethiopia; [40]Department of Anatomy, Madda Walabu University, Bale Robe, Ethiopia; [41]Department of Nursing, Dire Dawa University, Dire Dawa, Ethiopia; [42]College of Medicine and Health Sciences, Wollo University, Dessie, Ethiopia; [43]Water, Sanitation and Hygiene Unit, Ethiopian Public Health Institute, Addis Ababa, Ethiopia; [44]Department of Pharmacology, Bahir Dar University, Bahir Dar, Ethiopia; [45]Pharmacy Department, Alkan Health Science, Business and Technology College, Bahir Dar, Ethiopia.

Corresponding Author: Amanuel Yigezu; yigezuamanuel@yahoo.com

Submitting Author: Awoke Misganaw

- Main text word count: Current: 4487

For peer review only



For peer review only - http://bmjopen.bmj.com/site/about/guidelines.xhtml

For peer review only

**Abstract**

**Objective:** This analysis is to present the burden and trends of morbidity and mortality due to lower respiratory infections, their contributing risk factors, and the disparity across administrative regions and cities from 1990 to 2019.

**Design:** This analysis used Global Burden of Disease 2019 framework to estimate morbidity and mortality outcomes of lower respiratory infection and its contributing risk factors. The Global Burden of Disease study uses all available data sources and Cause of Death Ensemble model to estimate deaths from lower respiratory infection and a Meta-Regression Disease Modeling technique to estimate lower respiratory infection non-fatal outcomes with 95% uncertainty intervals.

**Study setting:** The study includes nine region states and two chartered cities of Ethiopia

**Outcome Measures:** We calculated incidence, death, and years of life lost (YLLs) due to LRIs and contributing risk factors using all accessible data sources. We calculated 95% uncertainty intervals (UI) for the point estimates.

**Results:** In 2019, LRIs incidence, death, and YLLs among all age groups were 8,313.7 (95% UI: 7,757.6–8,918), 59.4 (49.8–71.4) and 2,404.5 (2059.4–2833.3) per 100,000 people, respectively. From 1990, the corresponding decline rates were 39%, 61%, and 76%, respectively. Children under the age of five years account for 20% of episodes, 42% of mortalities, and 70% of the YLL of the total burden of LRIs in 2019. The mortality rate was significantly higher in predominantly pastoralist regions–Benishangul-Gumuz 101.8 (84.0–121.7) and Afar 103.7 (86.6–122.6). The Somali region showed the least decline in mortality rates. More than three-fourths of under-five child deaths due to LRIs were attributed to malnutrition. Household air pollution from solid fuel attributed to nearly half of the risk factors for all age mortalities due to LRIs in the country.



For peer review only - http://bmjopen.bmj.com/site/about/guidelines.xhtml

**Conclusion:** In Ethiopia, LRIs have reduced significantly across the regions over the years (except in elders), however are still the third leading cause of mortality, disproportionately affecting children younger than five years old, and predominantly pastoralist regions. Interventions need to consider leading risk factors, targeted age groups and pastoralist and cross-border communities.

***Keywords***: *Lower respiratory infections, regions, chartered cities, Ethiopia*

**Strengths and limitations of this study**

- The analysis has considered political, government and administrative changes of regional states and cities over the years to map available data and populations
- The analysis used all available data identified through an extensive collaboration effort and involving more than 700 leading researchers and policy makers from Ethiopia
- When data were not available for a particular regional states or city, the modelling process used data from other locations borrowing strength from geographic locations and time, and use predictive covariates
- However, limited quality data availability and accessibility for the analysis resulted in wider 95% Uncertainty Intervals which largely affect policy debates, prioritization, and health decisions.

## INTRODUCTION

Lower Respiratory Infections (LRIs) have been a predominant health problem worldwide, causing more than 2.3 million deaths in 2016 alone, amounting to a mortality rate of 32.2 per

For peer review only



For peer review only - http://bmjopen.bmj.com/site/about/guidelines.xhtml

100,000 people (1). LRIs comprise diseases of the lower airways such as pneumonia, bronchitis, and bronchiolitis, among others (2). Nearly all (99%) of LRI deaths occur in low- and middle-income countries and highly affect children under the age of five years (3). In sub-Sahara African countries, the mortality rate is 66.4 per 100,000 people, which is four times the mortality rate in East Asia and twice the global average (2).

Ethiopia ranked in the top three African countries in the number of under-five child deaths from LRIs (4). To prevent child death from LRIs and other diseases in early life, Ethiopia has been implementing the "Integrated Management of Childhood Illness" program since 1997 later scaled up to the national level in 2007 (5). LRIs, and pneumonia in particular, have been among the top three leading causes of childhood mortality in the country (6). Among LRIs' aetiologies, S. pneumonia contributed to more deaths than the other LRIs aetiologies combined (3). In 2011, the country introduced 10-valent pneumococcal conjugate vaccine (PCV 10) into its national immunization program to reduce the burden of Streptococcal pneumonia (7).

Morbidity and mortality from LRIs are attributable to multiple underlying factors. Malnutrition is one of the main underlying risk factors (8, 9). The other main attributable factor are poor living conditions that include household crowding, parental smoking, high use of household solid fuel/biomass consumption, poor ventilation, and lack of hand-washing facilities (10-12). In addition, bottle feeding also contributes to the burden of LRIs in children (13).

The flagship Ethiopian Health Extension Program (HEP) has been the backbone of the country's health system strategies to reduce the burden of LRIs and other diseases through preventive and health promotion activities at the community level. The HEP has also improved broader access to health care, availability of essential antibiotics, and immunization mainly to the rural population since 2004 (14). In 2010, the country also introduced the "integrated community case



For peer review only - http://bmjopen.bmj.com/site/about/guidelines.xhtml

management" (ICCM) approach to treat pneumonia through trained health cadres of health extension workers (HEWs) implementing HEP (15).

Currently, Ethiopia is implementing its Health Sector Transformation Plan-2 (HSTP-2) which is adapted from the Sustainable Development Goals. Some of the aims include increasing the proportion of under-five children with pneumonia who received antibiotics from 48% to 69% and improving full vaccination coverage from 44% to 69% between 2020 and 2025 (16). Commitment to international goals such as The Global Action Plan for the Prevention and Control of Pneumonia and Diarrhoea by 2025 could be reached if enough investment is made in high LRI burden countries like Ethiopia (17).

As Ethiopia is a country of stark contrasts in socioeconomic, epidemiological, and geographical variations, estimating disease burden at the regional level could provide valid and reliable information to inform policy decisions, including efficient resource allocation to match the burden in the subnational states. Hence, this article presents the 2019 Global Burden of Diseases, Injuries, and Risk Factors study (GBD) results on the burden, trends and regional variations of LRIs in Ethiopia from 1990 to 2019.

## METHODS

### Study Setting

Ethiopia is the second-most populous country in Africa next to Nigeria, with an estimated population of 112 million in 2019 (18). More than half of the country's population is under 20, and over 80% of the population resides in rural areas (19). The country is subdivided into ten regional states (Afar, Amhara, Benishangul-Gumuz, Gambella, Harari, Oromia, Somali, Sidama, Southern Nations and Nationalities and Peoples (SNNP), and Tigray) and two chartered cities



For peer review only - http://bmjopen.bmj.com/site/about/guidelines.xhtml

(Addis Ababa and Dire Dawa). During this study, Sidama was a zonal administration under the SNNP region. Oromia, Amhara, and SNNP are the highly populated regions. In this study, we classified the regions into urban (Addis Ababa, Dire Dawa, and Harari), agrarian (Oromia, Amhara, SNNP, Tigray), and pastoralist (Benishangul-Gumuz, Afar, Gambella, and Somali). The socio-economy of the regions such as income per person, educational attainment, and total fertility rate (TFR) varies as measured in socio-demographic index (SDI) (20) (Figure 1).

The healthcare system of the country is a three-tiered system consisting of primary, secondary, and tertiary levels of healthcare delivery units with 21,154 functioning health facilities and 159,545 health workforce in 2019 (16). The primary health care unit (PHCU) consists of health posts (staffed by HEWs), health centres, and primary hospitals. The secondary level of care consists of general hospitals and the tertiary level of care includes national referral hospitals which provide specialized services (16).

**Data Sources and Analysis**

The analysis and findings of LRIs presented in this analysis were produced by the Ethiopia Subnational Burden of Disease Initiative, a collaborative endeavour between the National Data Management Center for Health (NDMC) at the Ethiopian Public Health Institute (EPHI) and the Institute for Health Metrics and Evaluation (IHME), as part of GBD. The details of the methodology were described elsewhere (21). In brief, woreda (district) level geographic boundary mapping of regions and cities was used because woredas were relatively stable government structures (compared to lower or higher level administrative structures) during political or government changes and through the three census years (1984, 1994, and 2007). First, the analysis was estimated by mapping population and demography at the district level by



For peer review only - http://bmjopen.bmj.com/site/about/guidelines.xhtml

time and region. Then, the data sources were mapped by regions before processing the data in the GBD analysis based on GBD protocol. EPHI, in collaboration with IHME, gathered all accessible data sources by location for Ethiopia and all regions and cities that included census, demographic surveillance, household surveys, diseases registry, health service utilization, disease notification, and other data for this analysis. A comprehensive description of data sources, quality, and modelling for GBD 2019 has been reported on the following online portal: (http://ghdx.healthdata.org/gbd-2019/data-input-sources). This study outputs predates the COVID-19 and civil war occurred in the northern and other parts of the country and does not include the impact of COVID-19 or civil war.

## GBD Methods and Tools

The GBD details are reported elsewhere (22). Diseases and injuries within the GBD were organized into levels: Level 1 being the broadest causes of death and disability to Level 4 being the most specific. Within the three Level 1 causes (communicable, maternal, neonatal, and nutritional diseases; non-communicable diseases; and injuries), there were 174 Level 3 causes. The GBD 2019 study has estimated the burden of disease, including LRIs, for Ethiopia's national and subnational states. LRIs comprise diseases of the lower airways such as pneumonia, bronchitis, and bronchiolitis, among others (2). LRI mortality was estimated by age, sex, geography, and year using a modelling platform called the Cause of Death Ensemble model. LRI morbidity, including incidence, was modelled using a meta-regression platform known as DisMod-MR, a Bayesian, hierarchical, mixed-effects meta-regression platform (23). Years of life lost (YLLs) were computed by multiplying cause-specific deaths by the life expectancy at the age of death (24, 25). Population risk assessments over time and among risks were estimated using the comparative risk assessment approach developed for the GBD study (26, 27). The



For peer review only - http://bmjopen.bmj.com/site/about/guidelines.xhtml

GBD risk factors were categorized as follows: Level 1 risk factors are behavioural, environmental, occupational, and metabolic; Level 2 risk factors include 20 clusters of risks; Level 3 consists of 52 clusters of risks; and Level 4 contains 69 specific risk factors. All metrics were estimated separately for Ethiopia's nine regions and two chartered cities, and are presented with their 95% uncertainty intervals (UIs). All estimates produced for GBD report 95% uncertainty intervals (UIs) that account for sampling and non-sampling error associated with data and various assumptions of the modelling process and are derived from the 2·5th and 97·5th percentiles of 1000 draws(22, 28).

**Presentation of results**

We present the burden of LRIs in Ethiopia and its regional states using incidence, deaths, and YLLs categorized by sex, age groups, and year. We used numbers, rates, and percent change for the quantification of the burden. We also estimated the risk factors contributing to LRIs in Ethiopia and the percent change between 1990 and 2019. We reported GBD causes and risk factors using level 3 classifications, with 95% Uncertainty Intervals (UI). Additional tables and figures are attached in the supplementary materials.

**Patient and public involvement**

Patients and the public were not involved in the design of the study.

## RESULTS

### Morbidity Due to LRIs



For peer review only - http://bmjopen.bmj.com/site/about/guidelines.xhtml

In 2019, an estimated 6,628,673.6 (95% UI: 6,108,786.2–7,230,986.3) new cases of LRIs occurred in Ethiopia resulting in an age-standardized incidence rate of 8,313.7 per 100,000 people (7,757.6–8,918). Out of the total LRI episodes, 22% (1,448,680.0 new cases [1,150,089.8–1,799,704.4]) of new cases occurred among children younger than 5 years, yielding an annual incidence rate of 8,685.0 per 100,000 children (6,895.1–10,789.8). In adults older than 70 years, there were 725,273.3 (640,315.4–837,746.3) new cases of LRIs with an annual incidence of 38,394.4 per 100,000 people (33,896.9–44,348.5) (Figure 2; Supplemental Table 1 and Table 2).

Compared to national estimates, a significantly lower age-standardized incidence rate of LRIs per 100,000 people was observed in the chartered cities (6,788.1 [6,285.1–7,339.1] in Addis Ababa, 7,148.8 [6,634.6–7,750.7] in Dire Dawa), and in Harari region (7,190.5 [6,684.1–7,718.6]). The highest rates of age-standardized incidence per 100,000 people were observed in Afar (9,350.2 [8,648.8–10,157.4]), Somali (9,220.0 [8,515.9–10,046.4]), and Benishangul-Gumuz (9,054.6 [8,394.2–9,766.0]) although not significant compared to the national estimate (Figure 2; Supplemental Figure 1 and Table 2).

In children younger than 5 years, the lowest incidence rates were observed in Addis Ababa (4,927.2 [3,812.5–6,231.8]), Harari (6,821.6 [5,373.5–8,607.3]), and Gambella (6,791.1 [5,370.4–8460.1]) per 100,000 people, substantially below the national estimate. The highest incidence of LRIs per 100,000 children were recorded in Benishangul-Gumuz (10,481.8 [8,269.5–13,219.0]), Somali (10,062.7 [7,949.6–12,802.8]), Afar (9,365.5 [7,421.7–11,714.7]), and Oromia (9,039.4 [7,080.5–11,277.7]).

The age-standardized decline rate between 1990 and 2019 was 39% for both sexes, and it was 56% in under-five children and 13% in adults older than 70. The lowest decline in age-



For peer review only - http://bmjopen.bmj.com/site/about/guidelines.xhtml

standardized incidence rate was in Somali (19%); while it was between 38% and 44% for the remaining regions (Supplemental Table 2).

In children younger than 5, the incidence rates increased slightly between 1990 and 1995, except in Addis Ababa and Amhara region. The highest decline rates were found between the years 2005 and 2015 across all regions (Supplemental Figure 2).

**Mortality due to LRIs**

In 2019, LRIs caused 46,300.7 (95% UI: 39,515–54,642) deaths in Ethiopia, giving the age-standardized mortality rate of 86.4 (75.3-97.6) per 100,000 people. Under-five mortality accounted for 42% (19,591.8 [14,018.4–26,899.0]) of all deaths due to LRIs which resulted in a mortality rate of 117.4 (84.0–161.2) per 100,000 children. Of the under-five deaths, 71% (13,919.0 [9,946.0–18,860.0]) occurred in the first year of life. In adults older than 70 years, LRIs caused 14,627.6 (12,393.7–16,892.8) deaths, which was a mortality rate of 774.3 (656–894.2) deaths per 100,000 adults (Table 1; Supplemental Table 3).

In 2019, the number of deaths in all age groups by region were highest in Oromia (18,206 [15,193–21,745]), Amhara (9525 [7,530–11872]), SNNP (9,494 [7,713–11,649]), and Somali (3,907.5 [3,007.4–4,959.4]), followed by Tigray (2,551.4 [2,090.3–3,028.6]), Afar (706.2 [567.7–869.6]), Benishangul-Gumuz (619 [470.7–803.0]), Dire Dawa (154.6 [120.6–193.8]) and Gambella (123.2 [98.2–151.9]). Harari (93.1 [73.8–116.0) had the lowest number of deaths. (Supplemental Table 3).

Between 1990 and 2019, the age-standardized mortality rate declined by 61%, and the decline in under-five children and adults over 70 years was 86% and 30%, respectively (Table 1 and Supplemental Figure 3).



For peer review only - http://bmjopen.bmj.com/site/about/guidelines.xhtml

*Table 1: Lower Respiratory Infections mortality rates and percentage changes between 1990 and 2019 in Ethiopia, both sexes, with different age groups*

| | Age standardized | | Children younger than 5 | | | People older than 70 | | |
|---|---|---|---|---|---|---|---|---|
| Location | Deaths per 100 000 people [95% UI], 1990 | Deaths per 100 000 people [95% UI], 2019 | Deaths per 100 000 children [95% UI], 1990 | Deaths per 100 000 children [95% UI], 2019 | Cha-Nge,% | Deaths per 100 000 people [95% UI], 1990 | Deaths per 100 000 people [95% UI], 2019 | Cha-nge,% |
| Addis Ababa | 163.4[134.6-206.9] | 59.4[49.8-71.4] | 441.5[320.4-602.7] | 23.4[14.1-36.4] | 95 | 909.2[669.5-1264.6] | 503.6[410.5-633.7] | 45 |
| Oromia | 241.3[188.5-292.8] | 89.3[75.9-103.1] | 920.7[629.5-1264.1] | 122.3[84.8-171] | 87 | 1174.1[842.8-1543] | 849.5[687.3-1009] | 28 |
| Amhara | 197.7[163.5-236.5] | 74.3[59.4-91] | 691.3[531.7-883.4] | 94.9[55.6-146.1] | 87 | 1017.9[755.4-1324] | 682.7[537.4-857.9] | 33 |
| SNNPs | 243.7[196.4-296.9] | 98.9[83.8-116.6] | 971.9[697.2-1273.2] | 118.4[79-168.5] | 88 | 1161.3[584.7-822.3] | 845.4[685.6-018.1] | 28 |
| Tigray | 242.7[200.5-292] | 84.6[69.2-100.1] | 780.4[593.5-1009.1] | 67.2[43.5-98.3] | 92 | 1166.7[838.2-1586] | 788.6[637.9-956.8] | 33 |
| Harari | 240.2[182.6-303.1] | 77[63-92.5] | 1146.7[730-1604.1] | 89.9[51.1-138] | 93 | 762.3[412-1223.7] | 694.3[551.9-849] | 9 |
| Afar | 244.8[186.5-316.9] | 103.7[86.6-122.6] | 723.7[481.5-1023.7] | 101.7[65-152.4] | 86 | 1010[658-1541.7] | 917.6[720.6-146.8] | 10 |
| Somali | 147.6[113.8-192.6] | 97.5[79.2-118.6] | 455.9[313-628.5] | 197.3[135-279.8] | 57 | 798.8[519.2-1177.6] | 765.1[588.8-972.4] | 5 |
| BG | 284.1[221.8-358.1] | 101.8[84-121.7] | 1266.4[846.7-790.9] | 215.1[141.4-311] | 84 | 975.3[656.5-1379.3] | 671.9[532.7-848.7] | 32 |
| Dire Dawa | 220.1[171.6-270.9] | 69.9[56.5-84] | 1084.2[689.1-503.2] | 83.7[44.7-135.9] | 93 | 868.7[604.5-1205.5] | 621.3[496.4-766.1] | 29 |
| Gambella | 231.5[176.8-297.4] | 82.4[68.3-97.1] | 1265.5[805.4-764.3] | 57[32.4-89.6] | 96 | 739.6[477.7-1074.9] | 678.3[540.4-835.3] | 9 |
| Ethiopia* | 223[184.7-264.3] | 86.4[75.3-97.6] | 822.2[635-1051.2] | 117.4[84-161.2] | 86 | 1092.3[844-1391.5] | 774.3[656-894.2] | 30 |

N.B: all changes are in decreasing percent. SNNPs: Southern Nations, Nationalities, and Peoples; BG: Benishangul-Gumuz; *national estimate

For peer review only



For peer review only - http://bmjopen.bmj.com/site/about/guidelines.xhtml

In Ethiopia, the age-standardized mortality rate was higher among males (100.6 [84.1-121.4]) than females (71.8 [60.2–82.9]) (Supplemental Table 4).

In 1990, the age-standardized mortality rate per 100,000 people was the highest in Benishangul-Gumuz (284.1 [221.8–358.1]), Afar (244.8 [186.5–316.9]) and SNNP (243.7 [196.4–296.9]), although it is not significantly different from the national estimate. On the other hand, the lowest age standardized mortality rate was exhibited in Somali (147.6 [113.8–192.6]). Although the value was not significantly different from the national value in 1990, Addis Ababa showed the second lowest age-standardized mortality rate (163.4 [134.6–206.9]) (Table 1).

In 2019, the age-standardized mortality rate per 100,000 people was significantly lower in Addis Ababa (59.4 [49.8–71.4]) when compared to other regions, although not significantly lower than Gambella and other urban areas (Harari and Dire Dawa). Dire Dawa showed a significantly lower age-standardized mortality rate when compared to Benishangul-Gumuz, Afar, and SNNP. Compared to the national estimate, the regions of Afar (103.7 [86.6–122.6]) and Benishangul-Gumuz (101.8 [84.0–121.7]) recorded the highest age-standardized mortality rates per 100,000 people, although the difference is not statistically significant (Table 1). Compared to the 1990s, there was a 58% to 68% decrease in the age-standardized mortality rates across all regions. However, the Somali region recorded a 34% reduction in mortality rates (Supplemental Figure 3).

Among children below the age of five, the mortality rate per 100,000 people was significantly lower in Addis Ababa (23.4 ([14.1–36.4]) than other regions, although it was not significantly less than Gambella in 2019. The mortality rate was the highest in Benishangul-Gumuz (215.1 [141.4–311.0]), Somali (197.3 [135.2–279.8]), and Oromia (122.3 [84.8–171.0]) despite being not significantly higher than the national estimate (Table 1). Dire Dawa showed a significantly



For peer review only - http://bmjopen.bmj.com/site/about/guidelines.xhtml

lower mortality rate than Benishangul-Gumuz and Somali. Harari had a significantly lower mortality rate than Benishangul-Gumuz. For all regions, the mortality rate declined for children younger than 5-years between 1990 and 2019 by between 84% (in Benishangul-Gumuz) and 96% (in Gambella), except in Somali, which showed a 57% decline (Table 1). The mortality rate increased slightly between 1990 and 1995 and between 2010 and 2015 in Somali region (Supplemental Figure 4).

The mortality rate in adults older than 70 was the lowest in Addis Ababa (503.6 [410.5–633.7]). Other regions and cities have not shown a statistically significant difference from the national estimate. The decline in mortality rates between 1990 and 2019 is below 50% across all regions (Table 1). The mortality rate increased in Afar and Somali between 2005 and 2019 and in Gambella between 1990 and 2005 (Supplemental Figure 5).

**Premature Mortality due to LRIs**

In 2019, premature death due to LRIs was 2,445,093.7 (95% UI: 1,934,420.8–3,119,838.6) YLLs, yielding an age-standardized rate of 2404.5 per 100,000 people (2059.4–2833.3). Compared to 1990, the age-standardized YLL rate declined by 76% in 2019. In parallel, 70% of all premature mortality occurred in children younger than five, which accounted for 1,721,122.3 (1,231,032.1–2,362,958.7) YLLs. The YLL rate of 72,055.4 (55,718.5–92,064) per 100,000 under-five children in 2019 declined by 86% compared to the YLL rate in 1990. Adults over 70 years contributed 8% of all YLLs due to LRIs (194,756.2 [165,462.0–225,502.1]), yielding a rate of 10,309.9 (8759.2–11,937.6) YLLs per 100,000 people (Table 2; Supplemental Table 5 and Table 6).



For peer review only - http://bmjopen.bmj.com/site/about/guidelines.xhtml

Compared to 1990, the number of YLLs has decreased by 70% in all age groups and by 76% in children younger than five in 2019. However, the number of YLLs has increased by 45% in adults older than 70 (Supplemental Table 5).

The age-standardized YLL rate was significantly lower in Addis Ababa (1,285.6 [1,065–1,561.8]) in 2019 compared to the national average. The highest age standardized YLL rate were in Benishangul-Gumuz (3,571.1 [2,772.4–4,510.7]), Somali (3,236.4 [2,537.4–4,006]), and Afar (2,824.6 [2,323–3,414]), although not statistically significant compared to the national estimate (Table 2).

In children younger than five years, Addis Ababa (2,069 [1,253–3,217.5]) had a significantly lower YLL rate than the national estimate. The YLL rate was observed to be the lowest in Gambella (5,031.8 [2,867.2–7,885.7]), although not significantly lower than the national estimate (Table 2).

For peer review only

*Table 2: Lower Respiratory Infections YLL rates and percentage changes between 1990 and 2019 in Ethiopia, both sexes, with different age groups*

| | **Age-standardized** | | **Children younger than 5 years** | | | People older than 70 | | |
|---|---|---|---|---|---|---|---|---|
| **Location** | YLL per 100,000 people,1990 | YLL per 100,000 people, 2019 | YLL per 100,000 people,1990 | YLL per 100,000 people, 2019 | Change, % | YLL per 100,000 people,1990 | YLL per 100,000 people, 2019 | Change, % |
| Addis Ababa | 6362.2(5161.9-7847.9) | 1285.6(1065-1561.8) | 38824.7(28230.1-53092.4) | 2069(1253-3217.5) | 95 | 13587(9985.4-18978.3) | 6857.1(5545.8-8721.2) | 50 |
| Oromia | 11217.6(8517-14318) | 2433.8(2042.2-2879.5) | 80610.7(55103.7-110696) | 10742.8(7456.7-15039.6) | 87 | 18754.2(13297.1-24843) | 11119.7(8939.1-13305) | 41 |
| Amhara | 8690.4(7146.3-10453.8) | 2016.4(1551.4-2541.8) | 60663.3(46712.5-77511.5) | 8355(4898.9-12845) | 87 | 15950.4(11723.4-21059.7) | 9059.7(7063.2-11529.2) | 44 |
| SNNPs | 11633(9119.4-14477.9) | 2698.1(2243.5-3250.2) | 85114.7(61203.2-111502.1) | 10409.3(6968.1-14819.3) | 88 | 18842.6(13166.1-26081) | 11736.6(9426.7-14238.6) | 38 |
| Tigray | 10346.4(8522.9-12340.3) | 1977.1(1593.9-2395) | 68509.3(52185.7-88712.2) | 5927.9(3839.4-8676.8) | 92 | 19502.8(13914.1-26751.2) | 10486.8(8439.5-12765.3) | 47 |
| Harari | 12826.5(9336.5-16985.4) | 2060.4(1595.6-2623.1) | 100421.7(63898.7-140467.8) | 7922.5(4518.7-12117.2) | 93 | 11782.6(6360.4-19341.1) | 9260.4(7338.7-11340.3) | 22 |
| Afar | 10993.5(8520.8-14193.6) | 2824.6(2323-3414) | 63570.5(42296.6-89769) | 8943.7(5734.6-13390.2) | 86 | 16925.5(10923.7-26001.7) | 11867.1(9346-14716.9) | 30 |
| Somali | 6286.9(4860.5-7970) | 3236.4(2537.4-4006) | 40088.1(27543.7-55109) | 17314.3(11890.7-24530) | 57 | 11953.6(7599.2-17885.5) | 10246(7797.8-13150.6) | 15 |
| BG | 14965.1(11197.4-19998.6) | 3571.1(2772.4-4510.7) | 110847.2(74368-156588.4) | 18868.3(12442.9-27281.8) | 83 | 16397.3(10917-23228.5) | 9541.9(7476.9-12112.3) | 42 |
| Dire Dawa | 11749.7(8605.4-15249.5) | 1832.8(1407.3-2363.1) | 94905.3(60458.5-131584.5) | 7371.3(3953-11962.1) | 93 | 13207.9(9219.6-18328.2) | 8371(6616.3-10408.2) | 37 |
| Gambella | 13175.8(9291-17411) | 1937.4(1567.6-2344.6) | 110783.8(70576-154304.1) | 5031.8(2867.2-7885.7) | 96 | 11811.4(7673.5-17012.5) | 10030.9(7730.2-12664.9) | 16 |
| Ethiopia* | 10189.1(8347.5-12201.8) | 2404.5(2059.4-2833.3) | 72055.4(55718.5-92064) | 10318.6(7380.4-14166.6) | 86 | 17415.9(13362.7-22228.5) | 10309.9(8759.2-11937.6) | 41 |

SNNPs: Southern Nations, Nationalities, and Peoples; BG: Benishangul-Gumuz; *national estimate



For peer review only - http://bmjopen.bmj.com/site/about/guidelines.xhtml

The age-standardized premature mortality rate between 1990 and 2019 showed a continuous decline in all regions, except Somali. Benishangul-Gumuz showed the highest burden between 1990 and 2019 (Figure 3).

The change in premature mortality in children younger than five was significant throughout the years. However, the reduction in premature mortality among adults older than 70 was not as significant (Supplemental Figure 6 and Figure 7).

**Risk Factors**

Across all population groups, about half (48%) of the mortalities (measured in rates) due to LRIs in Ethiopia were attributed to household air pollution from solid fuel. In addition, lack of access to a hand-washing facility (23%), childhood wasting (23%), low birth weight (9%), short gestation period (7%), and ambient particulate matter pollution (6%) were also risk factors for mortality due to LRIs. The contribution of the risk factors to death due to LRIs in all regions was similar to the national estimate, except in Addis Ababa, where lack of access to hand-washing facilities, ambient particulate matter pollution, and low temperature were the main contributing factors. Ambient particulate matter pollution was also relatively higher in Dire Dawa, Harari, and Tigray regions (Supplemental Figure 8).

In children younger than five, more than three-fourths of deaths due to LRIs were attributed to childhood wasting (54%) stunting (12%), and child underweight (10%) in Ethiopia. In addition, 50% of the LRIs mortalities were attributed to household air pollution from solid fuel. Lack of access to hand-washing facilities (23%), low birth weight (23%), short gestation period (17%), high (4%) or low temperature (4%), absence of exclusive breastfeeding (4%) were also the risk factors with evident contribution. The distribution of risk factors varied among the regions. Child



For peer review only - http://bmjopen.bmj.com/site/about/guidelines.xhtml

wasting, low birth weight, and pre-term birth were the contributing factors of mortality in Addis Ababa. In Harari and Dire Dawa, child wasting, lack of access to hand-washing facilities, pre-term birth, and household air pollution from solid fuels contributed more to the death from LRIs than the other risk factors in 2019. All the risk factors except for ambient particulate air matter and low temperature were the highest in Somali and Benishangul-Gumuz (Supplemental Figure 9). When we examine the trend from 1990 to 2019, there is no significant reduction in the risk factors (Supplemental Figure 10).

## Discussion

The findings from this study indicate that although the burden of LRIs, measured in incidence or mortality rates, have shown a significant decline, they are still the third leading cause of death after neonatal disorders and diarrheal diseases in 2019 in Ethiopia. Cities and predominantly urban areas had lower mortality rates than predominantly pastoralist regions of the country. The rate of decline in mortality between 1990 and 2019 varied slightly across regions and chartered cities. The mortality rate decreased by more than three-fourths among children under the age of five and only by one-third among adults older than 70 between 1990 and 2019. Half of LRIs mortalities are attributed to household air pollution from solid fuels in all age groups. About three-fourths of LRIs in children were attributed to malnutrition.

The mortality rate of LRIs among children below the age of five has declined by 86% between 1990 and 2019. This decline could be attributed to improvements in living conditions, access to healthcare, and immunization. The national health delivery infrastructure has grown from 2,600 health facilities in 1997 to 21,154 facilities, including 314 hospitals, 3,678 health centers, and 17,162 health posts and private health facilities in 2019. As a result, the health workforce has

For peer review only

increased from 46,000 in 2007 to 159,545 in 2019 (16). The introduction of HEP since 2004 to provide preventive, health promotion, and curative treatment for pneumonia, malaria and diarrhoea had improved health outcomes for children (14). Moreover, Ethiopia has been implementing holistic child health improvement programs like IMCI since 1997 (15) and ICCM (5). Vaccination against Streptococcus pneumonia, which is responsible for about half of the LRIs mortality in African countries, with the PCV 10 vaccine since 2011 helped reduce the burden of LRIs (4). In addition, improved socioeconomic conditions have supported the reduction of the burden of LRIs in the country (29).

Despite the achievements reached in improving child health outcomes in Ethiopia, children are still disproportionately affected by LRIs. Out of the total burden of LRIs, 42% of all the deaths were among children younger than five. In that regard, this study has shown that one out of ten child deaths are due to LRIs. However, another study estimated that pneumonia alone shared about 17% of all deaths in children younger than five years (30). Among children under five, children younger than one carry the highest burden of LRIs. Furthermore, the mortality rate among children younger than one (397.7 per 100,000 people) was more than nine times higher than that of children between one and four years old (43 per 100,000 people). Although most studies on the high burden of LRIs corroborate our findings, the findings from Global Health Observatory (GHO) estimated a much higher mortality rate than the estimates of this study (i.e. 481.9 in children less than one year and 51.1 per 1000,000 population in children between one and four years) (8). Although we could not explain why this variation occurs, the estimates from GBD are also less than the estimate from findings of the Child Health Epidemiology Reference Group (CHERG), which is primarily due to the difference in the types of data used (31).



For peer review only - http://bmjopen.bmj.com/site/about/guidelines.xhtml

Urban areas of the country, mainly Addis Ababa and Dire Dawa, had significantly lower mortality rates than pastoralist areas such as Benishangul-Gumuz and Somali. These regional variations could be attributed to gaps in availability and access to healthcare and socioeconomic status differences among the subnational states. A previously conducted study indicated that disease burden is high among people in the poorest wealth quintile and people located mainly in Afar, Somali, Oromia, SNNP, and Benishangul-Gumuz and also have the lowest level of health service utilization (32). The rural area of the country has less healthcare coverage and utilization than the urban areas. The coverage of all basic vaccination is 43% in the country. Across the regions, the coverage of all basic vaccination is lowest in Afar (20%) and highest in Addis Ababa (83%). Coverage of pentavalent vaccines in children is 72% among the urban population while it is 56% among the rural population (15, 30). There is a variation in the performance of immunization across regions. Addis Ababa, Ethiopia's capital has PCV3 coverage of 93% among 1-year-olds in 2019, whereas this coverage is only 23% in Afar and Somali. The wasting rate in Somali was 21% in children under five, while it was just 2% in Addis Ababa in 2019 (33)

In addition to children, LRIs affects people older than 70 years. The number of people dying from LRIs increased over the years in people older than 70 years, partly due to the increase in the aging population. However, the mortality rate declined by 30% between 1990 and 2019 (3, 34). The incidence rate did not show a significant improvement across the study years, showing only 15% reductions between 1990 and 2019. Among the regions, Somali and Afar have recorded an increased incidence rate between 1990 and 2019. This could partly be explained by poor accessibility and availability of health facilities in the regions (33).

Wasting, stunting, and underweight were major risk factors contributing to the death of children younger than five years due to LRIs (35-37). More than 37% of children under five are stunted,



For peer review only - http://bmjopen.bmj.com/site/about/guidelines.xhtml

with a higher percentage in rural areas (41%) than in urban areas (26%). Similarly, the prevalence of child underweight and wasting is 21% in the rural and 7% in the urban parts of Ethiopia (38). This indicates that more investment is needed to reduce the burden on malnutrition among children and in the rural parts of the country to attain better health outcomes, protecting against LRIs. Although the prevalence of stunting, wasting, and underweight has decreased markedly over time, they are still major risk factors for death caused by LRIs. In the Millennium Development Goals (MDG) era, between 1990 and 2015, about half of the deaths averted due to LRIs were attributed to improvement in the nutritional status of children (reduction in wasting and stunting) (15).

Ambient particulate matter and household air pollution from solid fuel use were the two essential components of air pollution. Household air pollution from solid fuel use the was the second leading risk factor for LRIs, and ambient particulate matter is the eight leading risk factor among the top ten risk factors for LRIs (39-41). Our analysis also showed that there is a poor progress in the reductions of risk factors across the year, which shows there is a weak attempt in reducing the risk factors to prevent the population from LRIs. This indicates that improved use of electricity and natural gas for cooking and also appropriate investment in interventions that helps to reduce these risk factors will contribute to the reduction in the burden of the LRIs. (3).

To reduce the burden of LRIs, both national and global efforts are underway. The Global Action Plan for the Prevention and Control of Pneumonia and Diarrhoea (GAPPD), established by the World Health Organization, set goals in 2013 to reduce child LRI mortality rates to below 3 in 1,000 live births and to reduce severe LRI incidence by 75% of the 2010 baseline by 2025. To achieve these goals, reaching 90% of children with full-dose vaccine coverage, 90% access to pneumonia treatment, 50% coverage of exclusive breastfeeding in the first six months, and



For peer review only - http://bmjopen.bmj.com/site/about/guidelines.xhtml

exclusive breastfeeding promotion were set as prerequisites (17). However, a 6% average annual mortality reduction was recorded between 2000 and 2018 in Ethiopia. With this, the country can only reach the 2025 GAPPD target in 2035, ten years behind the target, according to the Maternal and Child Epidemiology Estimation Group (MICE) estimation (30).

Ethiopia's major health sector strategic plan, the Health Sector Transformation Plan-2 (HSTP-2) for the year 2021-2024, aims to reduce the infant mortality rate to 35, the neonatal mortality rate to 21 and the children under-5 mortality rate to 43 per 1,000 live births. Similarly, the 2030 Sustainable Development Goals (SDG) has set targets to reduce childhood mortality significantly. These targets can be achieved if the country implements high-impact priority curative and preventive interventions against LRIs (42). To achieve this, concerted action to improve policies, increase investment, foster innovations, and scale-up evidence-based interventions has paramount importance. Parallel to this, an estimated $274 billion for health is required to achieve the health-related SDGs by 2030 in 67 low and middle-income countries, including Ethiopia. One of the strategies to mobilize the needed resources is to increase government expenditure by 15% and share the population's costs through taxes or insurances (43).

This study is not without limitations; limitations in the GBD methods also apply to this study and limitations on Ethiopia subnational burden of disease that includes scarcity of quality data is published elsewhere (28).. When data on causes of death, morbidity or risk factors were not available for a particular regional state such as Afar or Somali, GBD modelled estimates use data from other locations and predictive covariates. Data sources such as household surveys have both sampling and non-sampling errors that account and led a wider 95% uncertainty intervals which might compromise the accuracy of the findings and reduce the use of these findings for policy

For peer review only

decision-making (1). Causes of mortality data sources used were mainly from verbal autopsy and sibling history having recall bias, broader category of causes of death report or poor generalizability to regional states (44). We used the best available data identified through an extensive collaboration effort and involving more than 700 leading researchers and policy makers from Ethiopia. The generation of estimates and their interpretation have benefited from intensive subnational review workshops and consultative meetings with domain experts.

## Conclusion

Despite the substantial reduction in morbidity and mortality at national and regional states, LRIs still remain one of the leading causes of the burden of disease in Ethiopia. Children and elders are still disproportionately affected by LRIs. The burden of illness and death due to LRIs varies across regional states in Ethiopia, with lower rates in cities and predominantly urban areas while predominantly pastoralist areas of the country have higher rates. Efforts should be made to tackle the major risk factors contributing to death by LRIs. Improving child nutrition, access to immunization and curative health services, as well as universal electrification to reduce indoor air pollution will be very useful strategies to reduce deaths due to LRIs in Ethiopia. Furthermore, improvement in socioeconomic factors will also help to reduce LRI burden at national and regional levels. To reach the targets set at the national and international level, mobilizing resources to health and improving the provision of health services to the community according to the needs of the regions is of paramount importance.

## List of abbreviations

EPHI: Ethiopian Public Health Institute; GAPPD: Global Action Plan for the Prevention and Control of Pneumonia and Diarrhoea GBD: Global Burden of Disease; GHO: Global Health

For peer review only



For peer review only - http://bmjopen.bmj.com/site/about/guidelines.xhtml

494 Observatory; HSTP: Health Sector Transformation Plan; ICCM: integrated community case management; IHME: Institute of Health and Metric Evaluation; LRIs: Lower Respiratory Infections; NDMC: National Data Management Center for health; PCV: Pneumococcal Conjugate Vaccine; SDG: Sustainable Development Goals; SNNPs: Southern Nations, Nationalities and Peoples; UI: Uncertainty Interval; WHO: World Health Organization; YLL: Years of Life Lost.

500 **Ethics approval and consent to participate**

501 This manuscript was produced as part of the GBD Collaborator Network and in accordance with the GBD Protocol.

503 **Consent for publication**

504 Not applicable.

505 **Availability of data and material**

506 All relevant data are submitted with this manuscript.

507 **Competing interests**

508 The authors declare that they have no competing interests.

509 **Funding**

510 Bill and Melinda Gates Foundation has funded Ethiopia subnational burden of disease EPHI-IHME collaborative initiative. The funder of this study had no role in study design, data collection, data analysis, data interpretation, or the writing of the report.

513 **Authors' contributions**

514 Authors contribution



For peer review only - http://bmjopen.bmj.com/site/about/guidelines.xhtml

Providing data or critical feedback on data sources

Semagn Mekonnen Abate, Mesafint Molla Adane, Addis Aklilu, Dejene Tsegaye Alem, Mulusew A Asemahagn, Hunegnaw Abebe, Melaku Ashagrie Belete, Tekleberhan Hailemariam, Tezera Moshago Berheto, Belay Boda Abule Bodicha, Daniel Baza Gargamo, Alemayehu Hailu, Awoke Misganaw, Mohsen Naghavi, Negussie Boti Sidamo, Yonatan Solomon, Shambel Wedajo, Melat Weldemariam, Amanuel Yigezu, Fentabil Getnet, and Yazachew Yismaw.

Developing methods or computational machinery

Semagn Mekonnen Abate, Tezera Moshago Berheto, Alemayehu Hailu, Mohsen Naghavi, Negussie Boti Sidamo, and Amanuel Yigezu.

Providing critical feedback on methods or results

Semagn Mekonnen Abate, Mesafint Molla Adane, Gizachew Taddesse Akalu, Addis Aklilu, Dejene Tsegaye Alem, Zeleke Gebru, Mulusew Andualem Asemahagn, Daniel Atlaw, Tewachew Awoke, Hunegnaw Abebe, Melaku Ashagrie Belete, Tekleberhan Hailemariam, Tezera Moshago Berheto, Alemeshet Yirga, Setognal Birara Aychiluhm, Belay Boda Abule Bodicha, Chuchu Churko, Feleke Mekonnen Demeke, Abebaw Alemayehu Desta, Lankamo Ena, Tahir Eyayu, Zinabu Fentaw, Daniel Baza Gargamo, Mesfin Damtew Gebrehiwot, Mathewos Alemu Gebremichael, Melaku Getachew, Ababi Zergaw, Alemayehu Hailu, Getahun Molla, Awoke Misganaw, Mohsen Naghavi, Biniyam Sahiledengle, Bereket Beyene, Migbar Sibhat, Negussie Boti Sidamo, Damtew Damtew Solomon, Yonatan Solomon, Birhanu Wagaye, Shambel Wedajo, Melat Weldemariam, Amanuel Yigezu, Fentabil Getnet, and Yazachew Yismaw.

Drafting the work or revising is critically for important intellectual content

For peer review only



For peer review only - http://bmjopen.bmj.com/site/about/guidelines.xhtml

Semagn Mekonnen Abate, Gizachew Taddesse Akalu, Mulusew A Asemahagn, Daniel Atlaw, Niguss Cherie Bekele, Melaku Ashagrie Belete, Tezera Moshago Berheto, Setognal Birara Aychiluhm, Belay Boda Abule Bodicha, Chuchu Churko, Tahir Eyayu, Zinabu Fentaw, Daniel Baza Gargamo, Melaku Getachew, Ababi Zergaw, Firehiwot Abebe Gobena, Muluken Argaw Haile, Alemayehu Hailu, Solomon Tessema Memirie, Awoke Misganaw, Mohsen Naghavi, Biniyam Sahiledengle, Bereket Beyene, Negussie Boti Sidamo, Yonatan Solomon, Dereje Mengistu Tolosa, Birhanu Wagaye, Ally Walker, Amanuel Yigezu, and Fentabil Getnet.

Managing the estimation or publications process

Semagn Mekonnen Abate, Awoke Misganaw, Mohsen Naghavi, Negussie Boti Sidamo, and Amanuel Yigezu.

**Declarations**

M A Gebremichael reports support for the present manuscript from the Bill and Melinda Gates Foundation.

**Acknowledgements**

The authors have no acknowledgements to declare

For peer review only

# References

1. Global, regional, and national age-sex specific mortality for 264 causes of death, 1980-2016: a systematic analysis for the Global Burden of Disease Study 2016. Lancet (London, England). 2017 Sep 16;390(10100):1151-210. PubMed PMID: 28919116. Pubmed Central PMCID: PMC5605883. Epub 2017/09/19. eng.

2. Troeger C, Blacker B, Khalil IA, Rao PC, Cao J, Zimsen SRM, et al. Estimates of the global, regional, and national morbidity, mortality, and aetiologies of lower respiratory infections

in 195 countries, 1990–2016: a systematic analysis for the Global Burden of Disease Study 2016. The Lancet Infectious Diseases. 2018;18(11):1191-210.

3. Moradi-Lakeh M, El Bcheraoui C, Charara R, Khalil I, Afshin A, Kassebaum NJ, et al. Burden of lower respiratory infections in the Eastern Mediterranean Region between 1990 and 2015: findings from the Global Burden of Disease 2015 study. International Journal of Public Health. 2018 2018/05/01;63(1):97-108.

4. Reiner RC, Welgan CA, Casey DC, Troeger CE, Baumann MM, Nguyen QP, et al. Identifying residual hotspots and mapping lower respiratory infection morbidity and mortality in African children from 2000 to 2017. Nature Microbiology. 2019 2019/12/01;4(12):2310-8.

5. Miller NP, Amouzou A, Tafesse M, Hazel E, Legesse H, Degefie T, et al. Integrated community case management of childhood illness in Ethiopia: implementation strength and quality of care. Am J Trop Med Hyg. 2014;91(2):424-34. PubMed PMID: 24799369. Epub 05/05. eng.

6. Alamneh YM, Adane F. Magnitude and Predictors of Pneumonia among Under-Five Children in Ethiopia: A Systematic Review and Meta-Analysis. Journal of Environmental and Public Health. 2020 2020/05/30;2020:1606783.

7. Wondimu A, Cao Q, Wilschut JC, Postma MJ. Factors associated with the uptake of newly introduced childhood vaccinations in Ethiopia: the cases of rotavirus and pneumococcal conjugate vaccines. BMC Public Health. 2019 2019/12/10;19(1):1656.

8. Gonakoti S, Osifo IF. Protein-Energy Malnutrition Increases Mortality in Patients Hospitalized With Bacterial Pneumonia: A Retrospective Nationwide Database Analysis. Cureus. 2021 Jan 12;13(1):e12645. PubMed PMID: 33585131. Pubmed Central PMCID: PMC7876587. Epub 2021/02/16. eng.

For peer review only



For peer review only - http://bmjopen.bmj.com/site/about/guidelines.xhtml

9. Yeo HJ, Byun KS, Han J, Kim JH, Lee SE, Yoon SH, et al. Prognostic significance of malnutrition for long-term mortality in community-acquired pneumonia: a propensity score matched analysis. The Korean journal of internal medicine. 2019 Jul;34(4):841-9. PubMed PMID: 30685962. Pubmed Central PMCID: PMC6610202. Epub 2019/01/29. eng.

10. Adane MM, Alene GD, Mereta ST, Wanyonyi KL. Prevalence and risk factors of acute lower respiratory infection among children living in biomass fuel using households: a community-based cross-sectional study in Northwest Ethiopia. BMC Public Health. 2020 2020/03/19;20(1):363.

11. Adane MM, Alene GD, Mereta ST, Wanyonyi KL. Effect of improved cookstove intervention on childhood acute lower respiratory infection in Northwest Ethiopia: a cluster-randomized controlled trial. BMC Pediatrics. 2021 2021/01/04;21(1):4.

12. Keleb A, Sisay T, Alemu K, Ademas A, Lingerew M, Kloos H, et al. Pneumonia remains a leading public health problem among under-five children in peri-urban areas of north-eastern Ethiopia. PLOS ONE. 2020;15(9):e0235818.

13. Ahmed KY, Page A, Arora A, Ogbo FA, Global M, Child Health Research c. Associations between infant and young child feeding practices and acute respiratory infection and diarrhoea in Ethiopia: A propensity score matching approach. PLOS ONE. 2020;15(4):e0230978.

14. Assefa Y, Gelaw YA, Hill PS, Taye BW, Van Damme W. Community health extension program of Ethiopia, 2003–2018: successes and challenges toward universal coverage for primary healthcare services. Globalization and Health. 2019 2019/03/26;15(1):24.

15. Ruducha J, Mann C, Singh NS, Gemebo TD, Tessema NS, Baschieri A, et al. How Ethiopia achieved Millennium Development Goal 4 through multisectoral interventions: a

For peer review only



For peer review only - http://bmjopen.bmj.com/site/about/guidelines.xhtml

Countdown to 2015 case study. Lancet Glob Health. 2017;5(11):e1142-e51. PubMed PMID: 29025635. eng.

16. Federal Ministry of Health. Health Sector Transformation Plan 2020/21-2024/25 (2013 EFY - 2017 EFY), Addis Ababa Ethiopia. 2020.

17. Unicef, World Health O. Ending preventable child deaths from pneumonia and diarrhoea by 2025 : the integrated global action plan for pneumonia and diarrhoea (GAPPD) 2013. Available from: https://apps.who.int/iris/handle/10665/79200.

18. World Bank. World Bank data catalog. [Washington, D.C.]: World Bank.

19. central statstical agency. Ethiopian statistical service. 2020. Available from: http://www.statsethiopia.gov.et/.

20. Burden of Disease Unit (BoD) NDMCNfh, Ethiopia Public Health Institute. Ethiopia Health Atlas, 2021 2021. Available from: https://ndmc.ephi.gov.et/national-health-atlas/.

21. Misganaw ANMWAMAHGAZBTMWEAKJHEGTWTKDDA. Progress in health among regions of Ethiopia, 1990-2019: a subnational country analysis for the Global Burden of Disease Study 2019. Lancet Lancet (London, England). 2022;399(10332):1322-35.

22. Murray CJL, Aravkin AY, Zheng P, Abbafati C, Abbas KM, Abbasi-Kangevari M, et al. Global burden of 87 risk factors in 204 countries and territories, 1990–2019: a systematic analysis for the Global Burden of Disease Study 2019. The Lancet. 2020;396(10258):1223-49.

23. Collaborators GL. Estimates of the global, regional, and national morbidity, mortality, and aetiologies of lower respiratory tract infections in 195 countries: a systematic analysis for the Global Burden of Disease Study 2015. Lancet Infect Dis. 2017;17(11):1133-61. PubMed PMID: 28843578. Epub 08/23. eng.



For peer review only - http://bmjopen.bmj.com/site/about/guidelines.xhtml

24. DALYs GBD, Collaborators H. Global, regional, and national disability-adjusted life-years (DALYs) for 315 diseases and injuries and healthy life expectancy (HALE), 1990-2015: a systematic analysis for the Global Burden of Disease Study 2015. Lancet (London, England). 2016;388(10053):1603-58. PubMed PMID: 27733283. eng.

25. Dicker D, Nguyen G, Abate D, Abate KH, Abay SM, Abbafati C, et al. Global, regional, and national age-sex-specific mortality and life expectancy, 1950–2017: a systematic analysis for the Global Burden of Disease Study 2017. The Lancet. 2018;392(10159):1684-735.

26. Lim SS, Vos T, Flaxman AD, Danaei G, Shibuya K, Adair-Rohani H, et al. A comparative risk assessment of burden of disease and injury attributable to 67 risk factors and risk factor clusters in 21 regions, 1990-2010: a systematic analysis for the Global Burden of Disease Study 2010. Lancet (London, England). 2012;380(9859):2224-60. PubMed PMID: 23245609. eng.

27. Collaborators GBDRF, Forouzanfar MH, Alexander L, Anderson HR, Bachman VF, Biryukov S, et al. Global, regional, and national comparative risk assessment of 79 behavioural, environmental and occupational, and metabolic risks or clusters of risks in 188 countries, 1990-2013: a systematic analysis for the Global Burden of Disease Study 2013. Lancet (London, England). 2015;386(10010):2287-323. PubMed PMID: 26364544. Epub 09/11. eng.

28. Misganaw A, Naghavi M, Walker A, Mirkuzie AH, Giref AZ, Berheto TM, et al. Progress in health among regions of Ethiopia, 1990–2019: a subnational country analysis for the Global Burden of Disease Study 2019. The Lancet. 2022;399(10332):1322-35.

29. Shibre G, Zegeye B, Idriss-Wheeler D, Yaya S. Trends of inequalities in care seeking behavior for under-five children with suspected pneumonia in Ethiopia: evidence from Ethiopia demographic and health surveys (2005–2016). BMC Public Health. 2021 2021/02/01;21(1):258.



For peer review only - http://bmjopen.bmj.com/site/about/guidelines.xhtml

30. Fighting for Breath in Ethiopia: a call to action to stop children dying from pneumonia [Internet]. 2020. Available from: stoppneumonia.org/wp-content/uploads/2019/11/Ethiopia-2019-Web_updated.pdf.

31. Kovacs SD, Mullholland K, Bosch J, Campbell H, Forouzanfar MH, Khalil I, et al. Deconstructing the differences: a comparison of GBD 2010 and CHERG's approach to estimating the mortality burden of diarrhea, pneumonia, and their etiologies. BMC Infectious Diseases. 2015 2015/01/16;15(1):16.

32. Federal Ministry of Health Ethiopia. Evidence Synthesis based on DHS key maternal and child health and nutrition indicators. 2019.

33. Federal Democratic Republic of Ethiopia-Ministry of Health. Mini Demographic and Health Survey 2019. 2019.

34. Corrêa RA, José BPS, Malta DC, Passos VMA, França EB, Teixeira RA, et al. Burden of disease by lower respiratory tract infections in Brazil, 1990 to 2015: estimates of the Global Burden of Disease 2015 study. Revista brasileira de epidemiologia = Brazilian journal of epidemiology. 2017 May;20Suppl 01(Suppl 01):171-81. PubMed PMID: 28658381. Epub 2017/06/29. Carga de doença por infecções do trato respiratório inferior no Brasil, 1990 a 2015: estimativas do estudo Global Burden of Disease 2015. por

eng.

35. Salam RA, Das JK, Bhutta ZA. Current issues and priorities in childhood nutrition, growth, and infections. The Journal of nutrition. 2015 May;145(5):1116S-22S. PubMed PMID: 25833888. Pubmed Central PMCID: PMC4410495. Epub 2015/04/03. eng.

36. Akombi BJ, Agho KE, Hall JJ, Wali N, Renzaho AMN, Merom D. Stunting, Wasting and Underweight in Sub-Saharan Africa: A Systematic Review. International journal of



For peer review only - http://bmjopen.bmj.com/site/about/guidelines.xhtml

environmental research and public health. 2017 Aug 1;14(8). PubMed PMID: 28788108. Pubmed Central PMCID: PMC5580567. Epub 2017/08/10. eng.

37. Ibrahim MK, Zambruni M, Melby CL, Melby PC. Impact of Childhood Malnutrition on Host Defense and Infection. Clinical microbiology reviews. 2017 Oct;30(4):919-71. PubMed PMID: 28768707. Pubmed Central PMCID: PMC5608884. Epub 2017/08/05. eng.

38. Fantay Gebru K, Mekonnen Haileselassie W, Haftom Temesgen A, Oumer Seid A, Afework Mulugeta B. Determinants of stunting among under-five children in Ethiopia: a multilevel mixed-effects analysis of 2016 Ethiopian demographic and health survey data. BMC Pediatrics. 2019 2019/06/01;19(1):176.

39. Dherani M, Pope D, Mascarenhas M, Smith KR, Weber M, Bruce N. Indoor air pollution from unprocessed solid fuel use and pneumonia risk in children aged under five years: a systematic review and meta-analysis. Bulletin of the World Health Organization. 2008 May;86(5):390-8C. PubMed PMID: 18545742. Pubmed Central PMCID: PMC2647443. Epub 2008/06/12. eng.

40. Kurmi OP, Lam KB, Ayres JG. Indoor air pollution and the lung in low- and medium-income countries. The European respiratory journal. 2012 Jul;40(1):239-54. PubMed PMID: 22362845. Epub 2012/03/01. eng.

41. Burnett RT, Pope CA, 3rd, Ezzati M, Olives C, Lim SS, Mehta S, et al. An integrated risk function for estimating the global burden of disease attributable to ambient fine particulate matter exposure. Environmental health perspectives. 2014 Apr;122(4):397-403. PubMed PMID: 24518036. Epub 2014/02/13. eng.

42. Federal Ministry of Health Addis Ababa E. Health Sector Transformation Plan 2015/16 - 2019/20 (2008-2012 EFY). . August 2015. .

For peer review only



For peer review only - http://bmjopen.bmj.com/site/about/guidelines.xhtml

43. Stenberg K, Hanssen O, Edejer TT-T, Bertram M, Brindley C, Meshreky A, et al. Financing transformative health systems towards achievement of the health Sustainable Development Goals: a model for projected resource needs in 67 low-income and middle-income countries. The Lancet Global Health. 2017;5(9):e875-e87.

44. Vos T, Lim SS, Abbafati C, Abbas KM, Abbasi M, Abbasifard M, et al. Global burden of 369 diseases and injuries in 204 countries and territories, 1990–2019: a systematic analysis for the Global Burden of Disease Study 2019. The Lancet. 2020;396(10258):1204-22.

For peer review only



For peer review only - http://bmjopen.bmj.com/site/about/guidelines.xhtml

707 *Figure 1: Socio-demographic index, Ethiopia, 2019*

708 *Figure 2: Incidence of lower respiratory infections per 100,000 people in Ethiopia and its*
709 *regions in 2019.*

710 *Figure 3: Trend in LRIs age standardized years of life lost rates per 100,000 people in Ethiopia,*
711 *1990-2019*

712

For peer review only



For peer review only - http://bmjopen.bmj.com/site/about/guidelines.xhtml

**Figure 1: Socio-demographic index, Ethiopia, 2019**

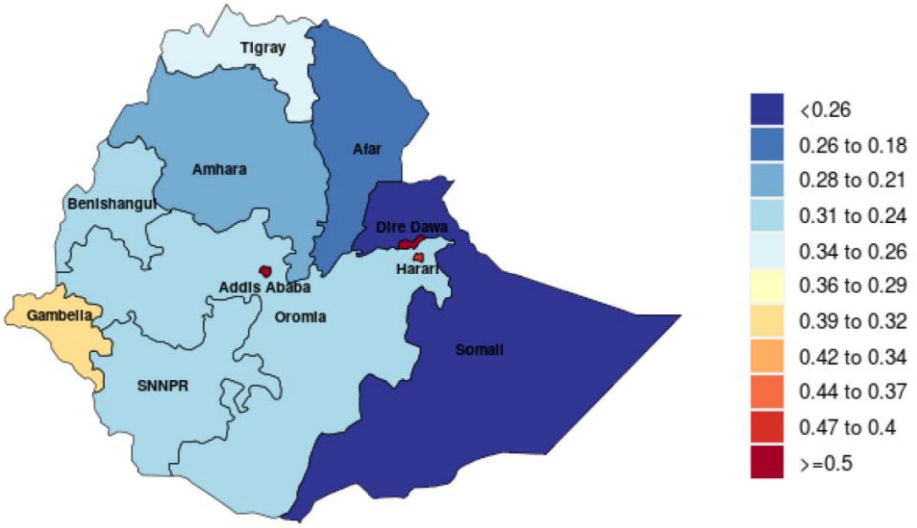


For peer review only - http://bmjopen.bmj.com/site/about/guidelines.xhtml

*Figure 2: Incidence of lower respiratory infections per 100,000 people in Ethiopia and its regions in 2019.*

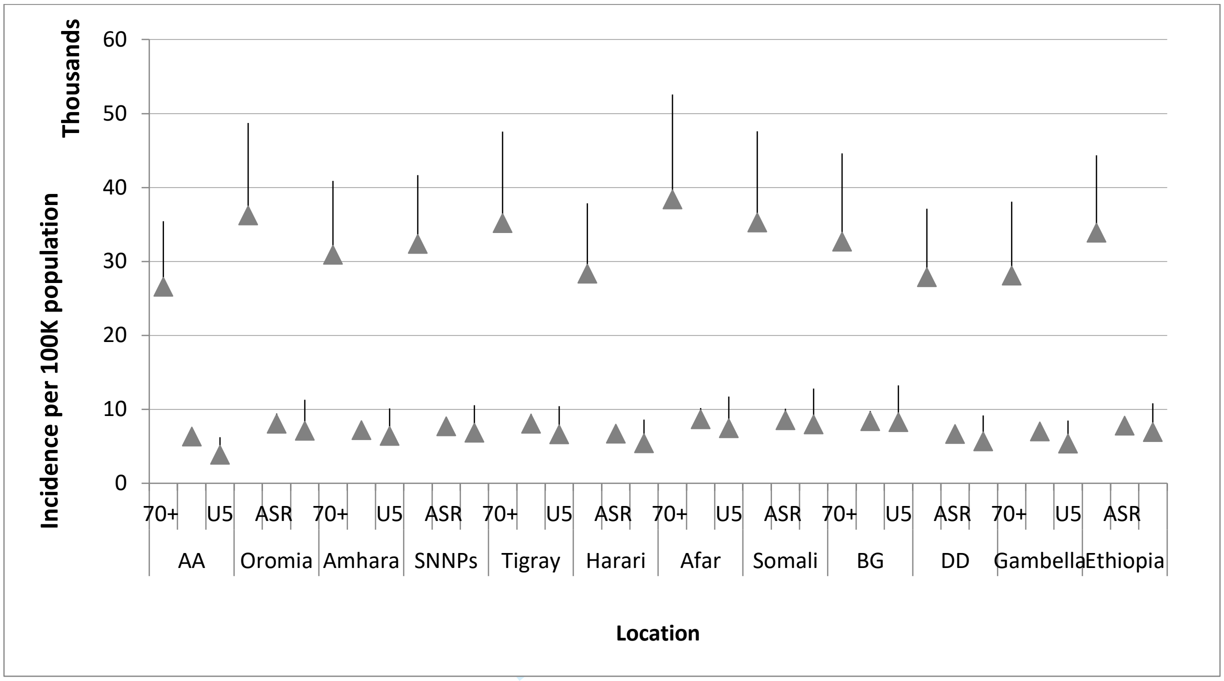


*Key: U5: children younger than 5 years; 70+: adults older than 70 years; ASR: Age-standardized rates; AA: Addis Ababa; DD: Dire Dawa; SNNPs: Southern Nations and Nationalities and Peoples; BG: Benishangul Gumuz*

For peer review only

Figure 3: Trend in LRIs age standardized years of life lost rates per 100,000 people in Ethiopia, 1990-2019

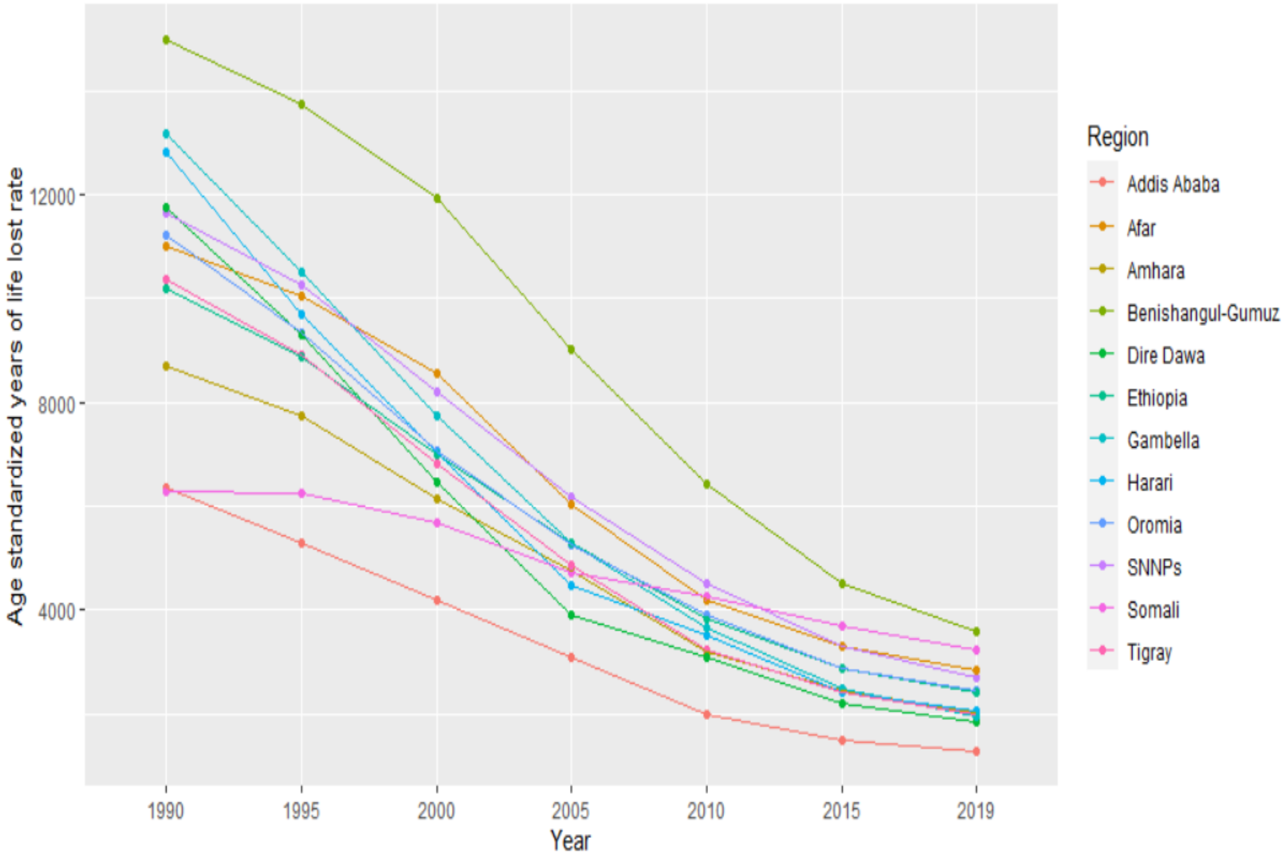

**Supplementary material to "The burden of lower respiratory infections and associated risk factors across regions in Ethiopia: A subnational analysis of the Global Burden of Diseases 2019 Study"**

This supplementary material provides supplemental figures and tables more detailed results for ""The burden of lower respiratory infections and associated risk factors across regions in Ethiopia: A subnational analysis of the Global Burden of Diseases 2019 Study"

## Contents

**Lists of Tables and Figures** .......... 2

Supplemental Figure 1: Trend in age-standardized incidence rate of LRIs per 100,000 people in Ethiopia, 1990-2019 .......... 2

Supplemental Figure 2: Children under 5 years of age incidence rate per 100,000 populations due to LRI in Ethiopia, 1990- 2019 .......... 3

Supplemental Figure 3: Trend in LRIs age-standardized mortality rates per 100,000 people in Ethiopia, 1990-2019 .......... 4

Supplemental Figure 4: Children younger than 5 years mortality rate per 100,000 populations for lower respiratory infections in Ethiopia, 1990-2019 .......... 4

Supplemental Figure 5: Adults older than 70 years mortality rate per 100,000 populations for lower respiratory infections in Ethiopia, 1990-2019 .......... 4

Supplemental Figure 6: Children younger than 5 years YLL per 100,000 populations for lower respiratory infections in Ethiopia, 1990-2019 .......... 5

Supplemental Figure 8: Attribution of the risk factors to LRIs death rate per 100,000 population in all age groups between 1990 and 2019 for Ethiopia and its regions, both sexes, number of death, 2019. SNNPs: Southern Nations, Nationalities, and Peoples. .......... 6

Supplemental Figure 10: Trends in the risk factors between 1990 and 2019 for all ages for Ethiopia .......... 8

Tables .......... 9

Supplemental Table 1: Number and percentage changes of episode attributable to LRIs in 1990 and 2019 for Ethiopia, its regions, both sexes .......... 9

Supplemental Table 2: Rate and percentage changes of episodes attributable to LRIs in 1990 and 2019 for Ethiopia and its regions, both sexes .......... 10

Supplemental Table 3: Number and percentage changes of death attributable to LRIs in 1990 and 2019 for Ethiopia and its regions, both sexes. .......... 11

Supplemental Table 4: Age standardized mortality rate, Ethiopia and its regions, by sex, 2019 .......... 12

Supplemental Table 5: Number and percentage changes of YLL attributable to LRIs in 1990 and 2019 for Ethiopia and its regions, both sexes .......... 12

Supplemental Table 6: Rate and percentage changes of YLL attributable to LRIs in 1990 and 2019 for Ethiopia and its regions, both sexes. .......... 13

For peer review only

Authors contributions ........................................................................................................................ 15

## Lists of Tables and Figures

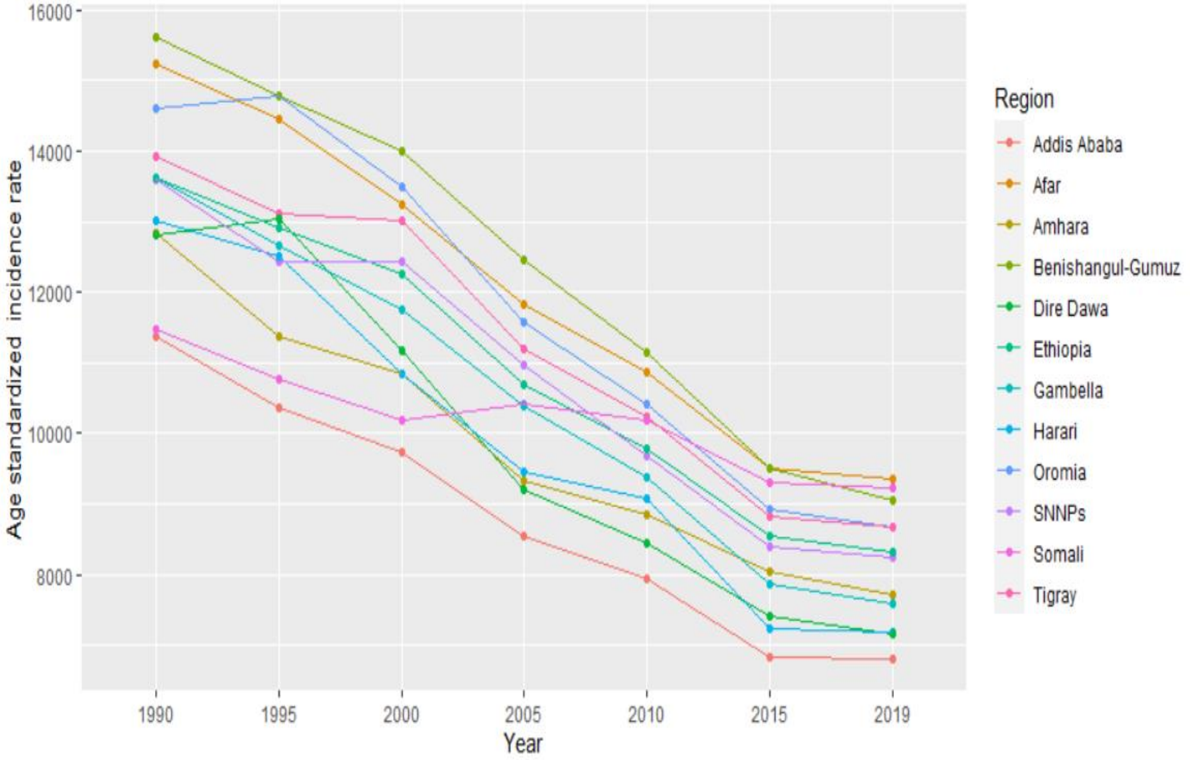


Supplemental Figure 1: Trend in age-standardized incidence rate of LRIs per 100,000 people in Ethiopia, 1990-2019

w only

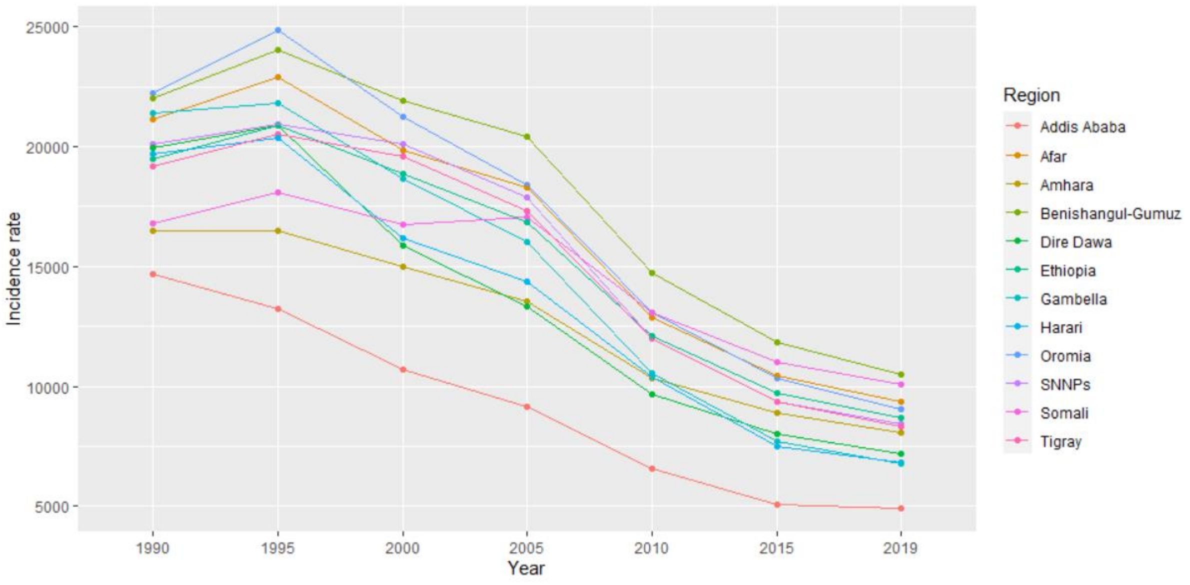


Supplemental Figure 2: Children under 5 years of age incidence rate per 100,000 populations due to LRI in Ethiopia, 1990- 2019

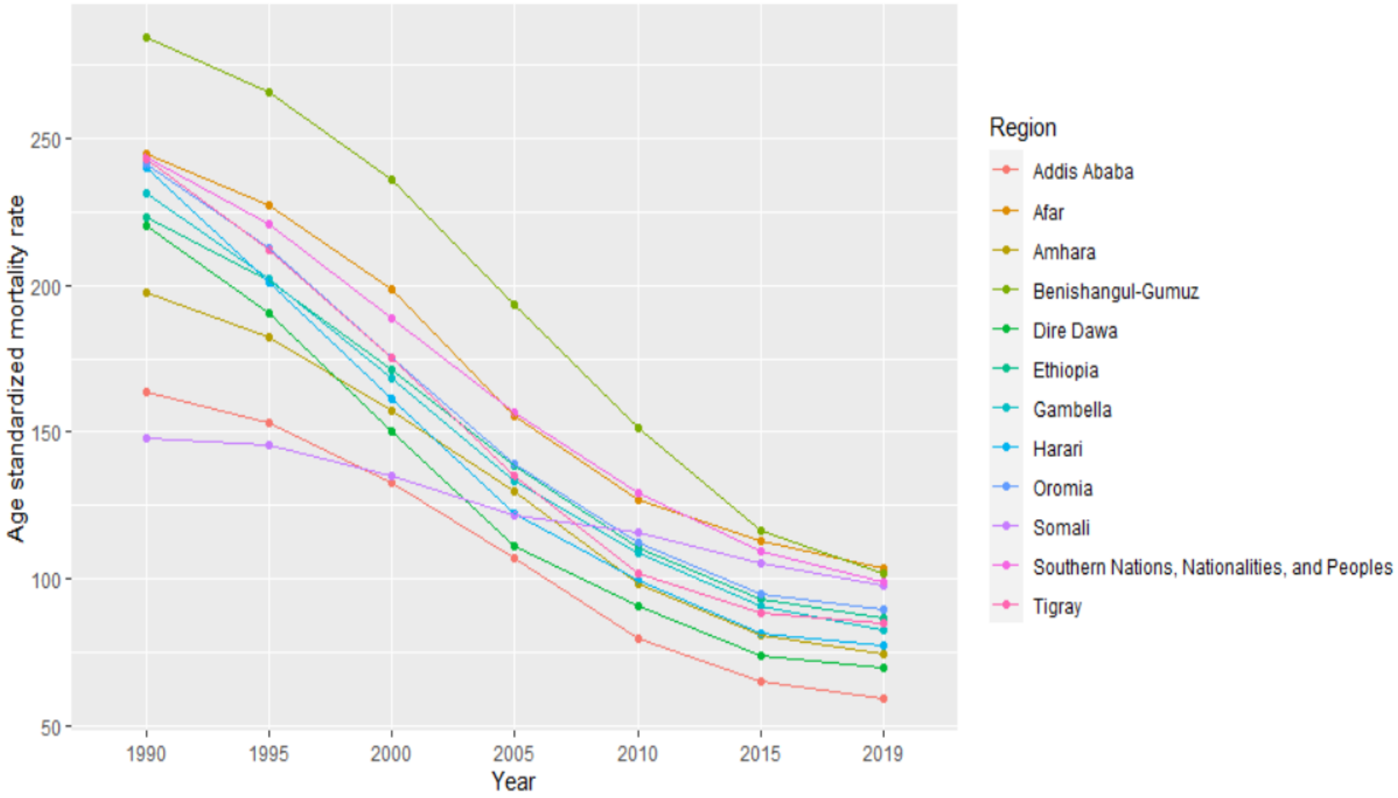

Supplemental Figure 3: Trend in LRIs age-standardized mortality rates per 100,000 people in Ethiopia, 1990-2019

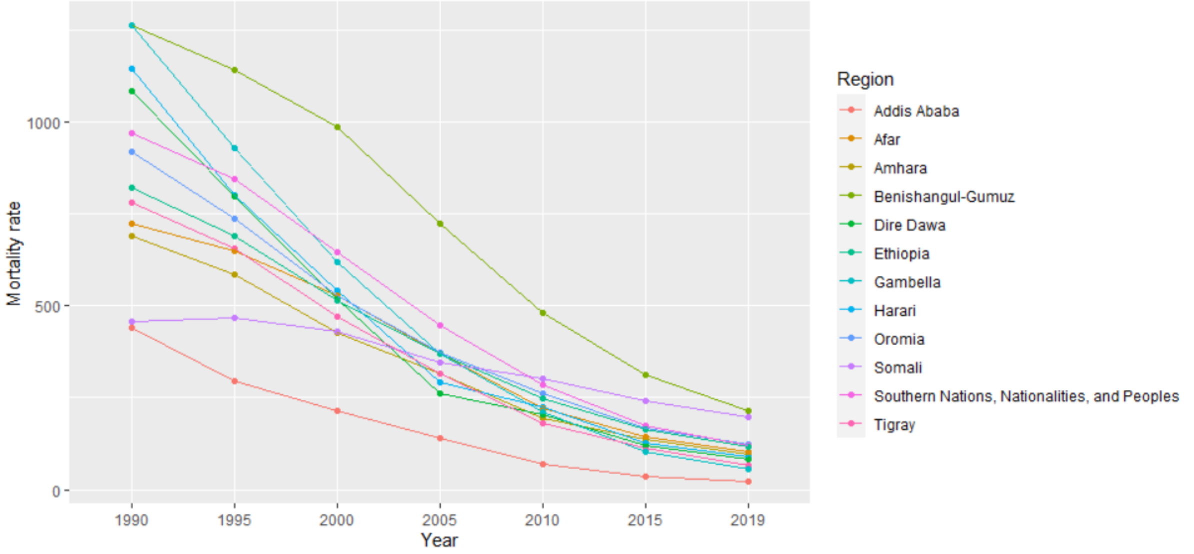


Supplemental Figure 4: Children younger than 5 years mortality rate per 100,000 populations for lower respiratory infections in Ethiopia, 1990-2019

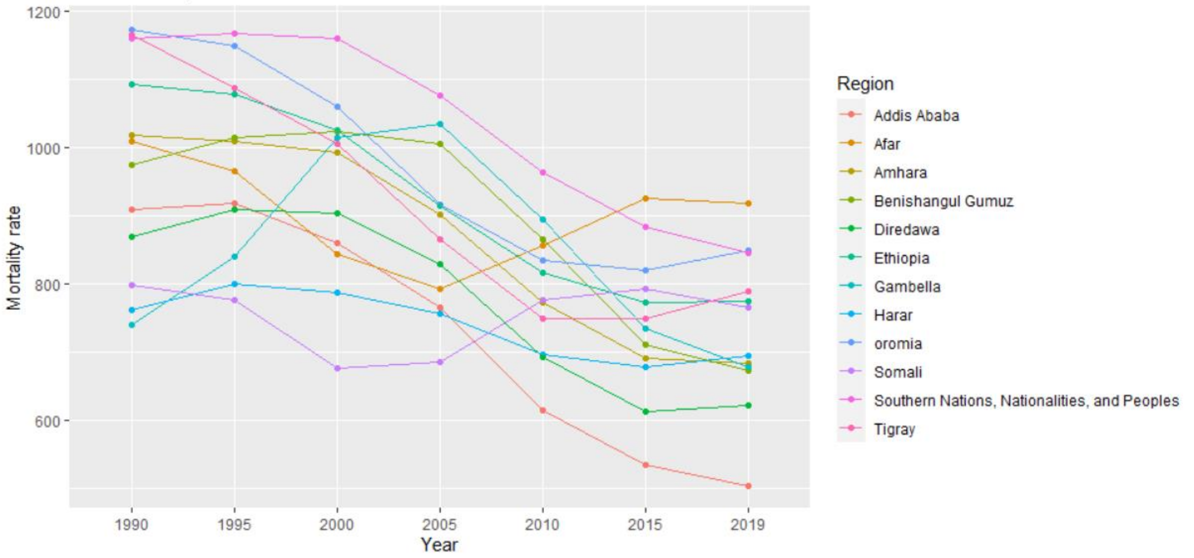


Supplemental Figure 5: Adults older than 70 years mortality rate per 100,000 populations for lower respiratory infections in Ethiopia, 1990-2019

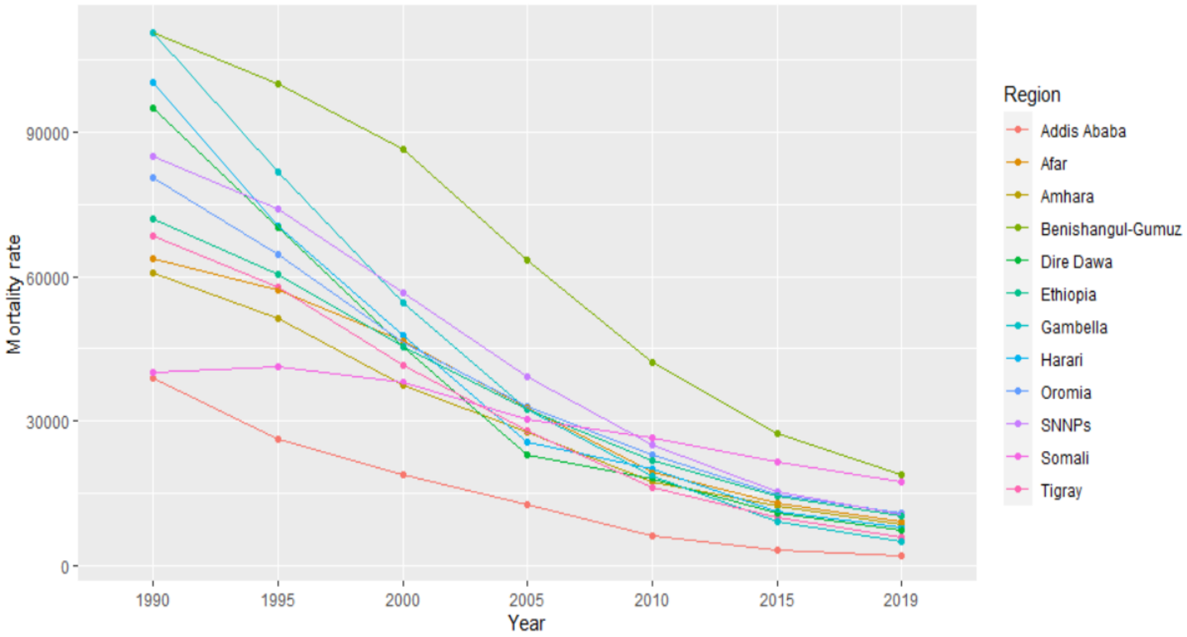


Supplemental Figure 6: Children younger than 5 years YLL per 100,000 populations for lower respiratory infections in Ethiopia, 1990-2019

For peer review only - http://bmjopen.bmj.com/site/about/guidelines.xhtml

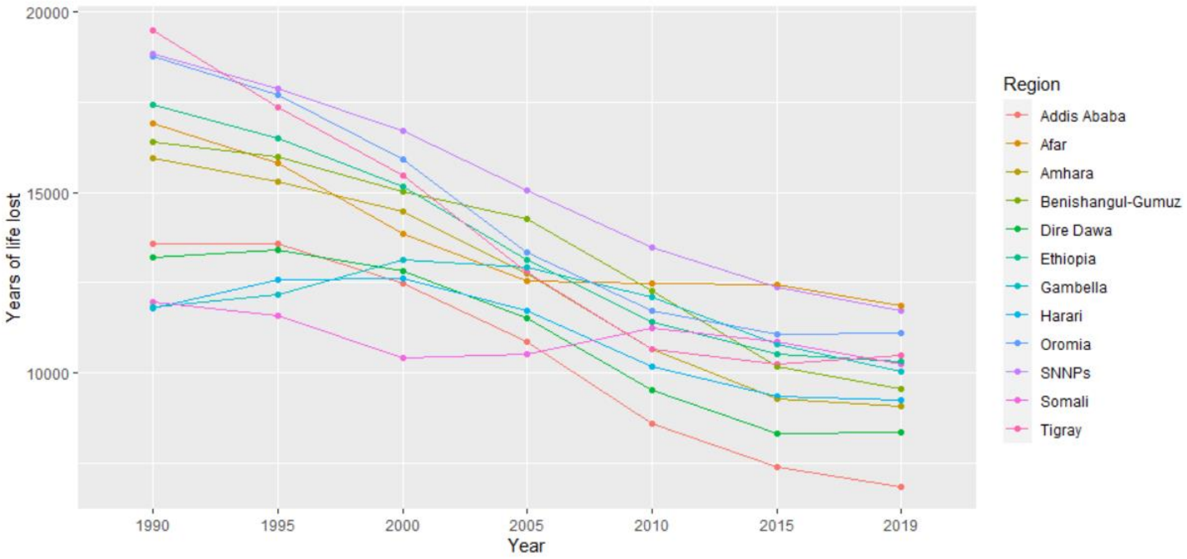


Supplemental Figure 7: Adults older than 70 years YLL per 100,000 populations for lower respiratory infections in Ethiopia, 1990-2019

| Location | Alcohol use | Ambient particulate matter pollution | Child stunting | Child underweight | child wasting | High temperature | Household air pollution from solid fuel- | Low birth weight | Low temperature | No access to handwashing facility | Non-exclusive breastfeeding | Secondhand smoke | Short gestation | Smoking |
|---|---|---|---|---|---|---|---|---|---|---|---|---|---|---|
| Addis Ababa | 1.4 | 4 | 0.1 | 0.1 | 0.7 | 0 | 1.7 | 0.7 | 3.7 | 4.8 | 0.1 | 0.4 | 0.7 | 1 |
| Oromia | 0.6 | 2.5 | 2.5 | 1.9 | 11.2 | 0.9 | 21.6 | 4.3 | 2.2 | 10.3 | 0.9 | 0.6 | 3.5 | 1.3 |
| Amhara | 0.4 | 2.5 | 1.8 | 1.4 | 6.7 | 0.9 | 19.8 | 3.3 | 2.4 | 9.5 | 0.4 | 0.6 | 2.6 | 0.4 |
| SNNPs | 0.8 | 2.4 | 2.7 | 2.2 | 10.2 | 1.8 | 19.9 | 3.5 | 1.4 | 9.6 | 0.8 | 0.5 | 2.9 | 0.9 |
| Tigray | 0.7 | 3.3 | 0.8 | 0.8 | 4 | 1.1 | 17.7 | 2.8 | 1.2 | 9.4 | 0.3 | 0.5 | 2.4 | 0.4 |
| Harari | 1 | 3.8 | 0.9 | 0.7 | 5.3 | 0 | 9.6 | 2.4 | 1.1 | 7.6 | 0.6 | 0.6 | 2.1 | 3.2 |
| Afar | 0.4 | 2.1 | 1.8 | 1.9 | 8.5 | 6.3 | 21 | 4.5 | 0.1 | 9.4 | 0.9 | 0.6 | 3.6 | 1.4 |
| Somali | 0.3 | 1.6 | 3.3 | 3.8 | 22.2 | 4.8 | 31.6 | 7.2 | 0.4 | 13.3 | 2.5 | 0.9 | 5.7 | 1.8 |
| Benishangul-Gumuz | 0.4 | 3.4 | 4.5 | 3.5 | 20 | 4.7 | 27.7 | 4.9 | 0.6 | 13.3 | 1.3 | 0.7 | 4 | 0.8 |
| Dire Dawa | 0.9 | 3.6 | 0.7 | 0.7 | 5.1 | 0.4 | 8.3 | 2.4 | 0.5 | 6.9 | 0.4 | 0.5 | 2 | 2.1 |
| Gambella | 0.8 | 2.2 | 0.4 | 0.4 | 3.2 | 2.5 | 9.3 | 2.2 | 0.1 | 5.4 | 0.4 | 0.3 | 1.9 | 2.1 |
| Ethiopia | 0.6 | 2.5 | 2.2 | 1.8 | 9.9 | 1.4 | 20.5 | 3.9 | 1.9 | 9.9 | 0.8 | 0.6 | 3.2 | 1 |

Supplemental Figure 8: Attribution of the risk factors to LRIs death rate per 100,000 population in all age groups between 1990 and 2019 for Ethiopia and its regions, both sexes, number of death, 2019. SNNPs: Southern Nations, Nationalities, and Peoples.

| | Ambient particulate matter pollution | child stunting | Child under weight | child wasting | high temperature | Household air pollution from solid fuels | Low birth weight | Low temperature | No access to handwashing facility | Non-exclusive breastfeeding | Secondhand smoke | Short gestation |
|---|---|---|---|---|---|---|---|---|---|---|---|---|
| Addis Ababa | 3.63 | 0.82 | 0.64 | 8.35 | 0.00 | 1.75 | 8.86 | 3.29 | 4.27 | 1.32 | 0.31 | 7.98 |
| Oromia | 6.41 | 14.75 | 11.45 | 67.14 | 2.34 | 60.08 | 25.95 | 6.05 | 28.32 | 5.25 | 1.66 | 21.03 |
| Amhara | 5.40 | 13.01 | 9.84 | 48.10 | 2.02 | 46.86 | 23.47 | 5.46 | 21.99 | 2.81 | 1.36 | 18.88 |
| SNNPs | 6.36 | 16.67 | 13.77 | 63.88 | 5.03 | 57.68 | 21.95 | 4.12 | 27.49 | 5.28 | 1.38 | 18.31 |
| Tigray | 5.03 | 6.15 | 5.75 | 29.88 | 1.87 | 29.89 | 20.59 | 2.00 | 15.32 | 1.91 | 0.79 | 17.45 |
| Harari | 9.12 | 7.21 | 5.80 | 43.66 | 0.01 | 25.51 | 19.63 | 2.74 | 19.30 | 5.00 | 1.62 | 17.18 |
| Afar | 4.88 | 11.42 | 11.57 | 52.76 | 15.77 | 53.12 | 28.03 | 0.26 | 23.50 | 5.49 | 1.33 | 22.31 |
| Somali | 5.40 | 18.08 | 20.92 | 121.83 | 16.76 | 110.21 | 39.76 | 1.46 | 46.07 | 13.99 | 3.18 | 31.30 |
| Benishangul-Gumuz | 12.63 | 28.22 | 22.25 | 125.72 | 17.81 | 105.51 | 30.75 | 2.22 | 50.23 | 8.05 | 2.61 | 24.89 |
| Dire Dawa | 9.38 | 5.50 | 5.64 | 42.47 | 1.11 | 23.88 | 19.72 | 1.42 | 18.88 | 3.67 | 1.50 | 16.71 |
| Gambella | 4.75 | 3.13 | 3.08 | 25.92 | 6.16 | 23.51 | 18.20 | 0.18 | 13.02 | 2.86 | 0.60 | 15.31 |
| Ethiopia | 6.06 | 14.39 | 11.93 | 64.00 | 4.28 | 58.22 | 25.08 | 4.73 | 27.20 | 5.22 | 1.58 | 20.40 |

Supplemental Figure 9: Attribution of the risk factors to LRIs death rate per 100,000 population in children younger than 5 years between 1990 and 2019 for Ethiopia and its regions, both sexes, number of death, 2019. SNNPs: Southern Nations, Nationalities, and Peoples.

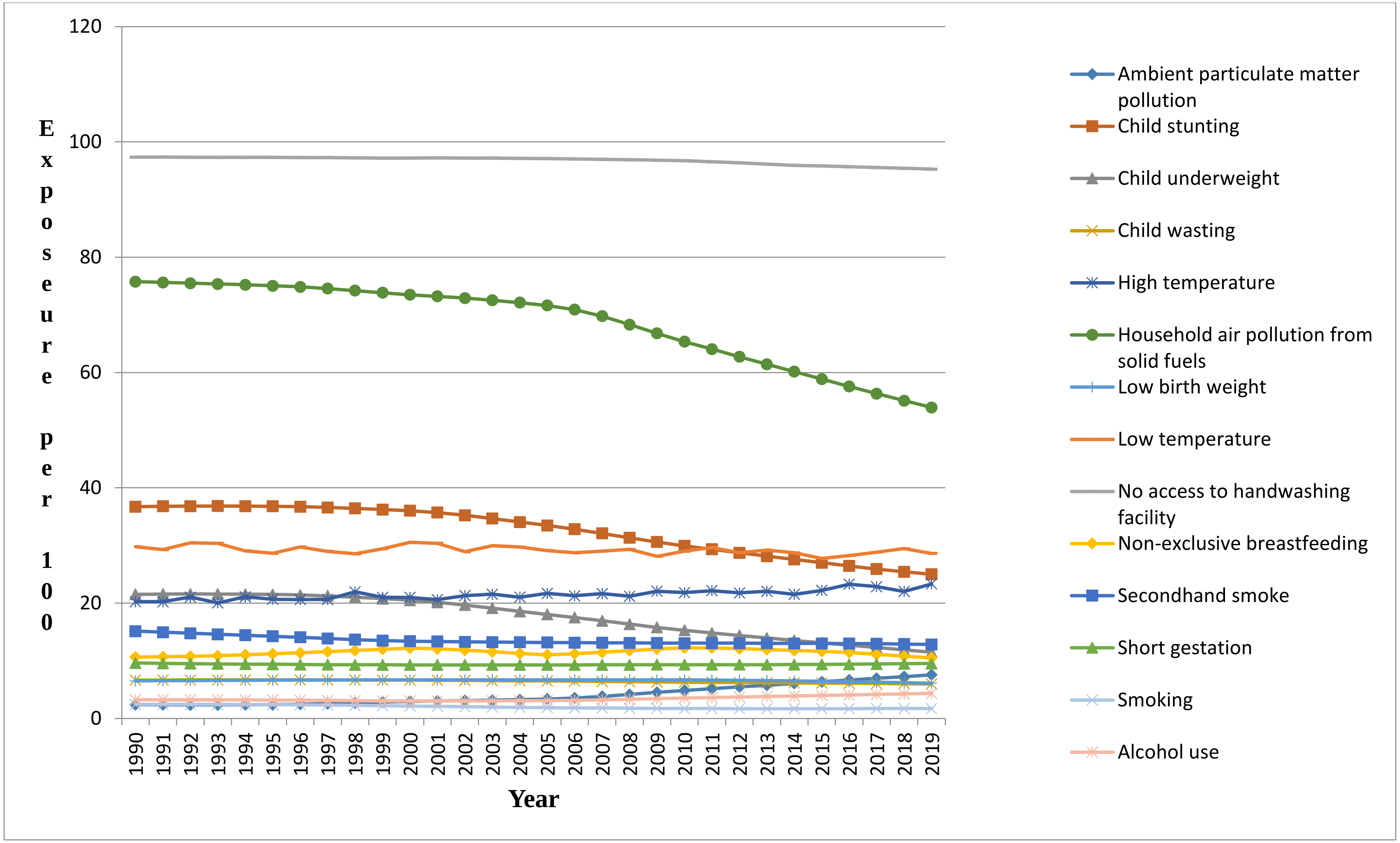


Supplemental Figure 10: Trends in the risk factors between 1990 and 2019 for all ages for Ethiopia

# Tables

## Supplemental Table 1: Number and percentage changes of episode attributable to LRIs in 1990 and 2019 for Ethiopia, its regions, both sexes

| | All ages | | | Children younger than 5 years | | | People above 70 | | |
|---|---|---|---|---|---|---|---|---|---|
| Location | Episode, (95%UI),1990 | Episode, (95%UI),2019 | Change, % | Episode, (95%UI),1990 | Episode, (95%UI),2019 | Change, % | Episode, (95%UI),1990 | Episode, (95%UI),2019 | Change,% |
| Addis Ababa | 194122.9(175403.8-213856.2) | 176027.6(162152.3-190557.8) | 10 | 44940.6(35673.3-56859.6) | 14522.3(11237-18367.5) | 68 | 8099.5(6891.6-9463.2) | 24034.2(20749.5-27702.3) | 196** |
| Oromia | 2164243.7(1957719.5-2397957.9) | 2597863.9(2372089.2-2859925.6) | 20 | 739239.6(578477-935022.8) | 613676.6(480687.2-765626.5) | 17 | 110627.2(95303.7-129579.9) | 292179.4(254670.4-342438.5) | 164** |
| Amhara | 1508431.4(1368059.8-1671550.1) | 1400682.4(1285323-1530939.8) | 8 | 423999.3(334700.8-538426.8) | 261397.4(206754.7-327889.7) | 39 | 116332.8(98774.4-137194.5) | 198977.5(173809.4-229925.5) | 71** |
| SNNPs | 1235554.9(1108317.9-1368520.3) | 1330491.5(1217176.7-1452694.1) | 7 | 421825.6(335087.1-526155.2) | 307771.3(248326.4-385053.7) | 28 | 68084.5(57450-79640.8) | 99723(88168.3-113438) | 46** |
| Tigray | 360795.1(328276.3-400714.9) | 409829.8(376290.4-445030.2) | 13 | 109047.9(86435.2-137691.1) | 69958.2(55210.7-87468.6) | 36 | 20653.3(17469.9-24256.6) | 62677.2(53933.1-72927.6) | 203** |
| Harari | 15145.9(13618.9-16851.9) | 13765.3(12657-15054.7) | 10 | 4693.6(3691.4-5990.1) | 2188.4(1723.8-2761.3) | 54 | 307.2(259.6-357) | 1614.8(1396-1863.6) | 425** |
| Afar | 113506.8(102317-126084.3) | 113210.3(102987.9-124578) | 1 | 32991.6(25672.4-42147.6) | 26177.2(20744.1-32743.3) | 21 | 2640.2(2212.5-3150.9) | 7222.5(6246.8-8559.1) | 173** |
| Somali | 300306.3(268988-336331.8) | 463220.4(420429.8-516009.4) | 54 | 103526.3(81391.3-131352.8) | 126089.6(99611.9-160423.3) | 21** | 6182.3(5316-7370.4) | 30203.2(25942.8-35038.5) | 388** |
| BG | 68501.6(61804.9-75736.4) | 71675.7(64972.1-79105.8) | 4 | 20970.5(16626.7-26454.5) | 18175.6(14339.5-22922.1) | 14 | 3288.9(2784.8-3911.4) | 4311.3(3743.8-5113.9) | 31** |
| Dire Dawa | 30607.1(27359.8-34252.2) | 26221.1(24010.1-28647.8) | 15 | 9547.4(7523-12185) | 4387.7(3439.6-5583.9) | 55 | 1274.8(1090.1-1483.5) | 2840.1(2469-3289.5) | 122** |
| Gambella | 19687.9(17723.9-21945.7) | 25685.2(23516.6-28093.2) | 30** | 6747.7(5338.7-8613.7) | 4335.4(3428.4-5400.9) | 36 | 1568.1(1316.6-1825.8) | 1489.6(1281.4-1737.3) | 6 |
| Ethiopia* | 6010904.2(5467801.6-6648644.3) | 6628673.6(6108786.2-7230986.3) | 10** | 1917530.8(1518850.1-2400978.5) | 1448680.4(1150089.8-1799704.4) | 25 | 339059.4(293491.9-387514.2) | 725273.3(640315.4-837746.3) | **113** |

SNNP: Southern Nations, Nationalities, and Peoples; BG: Benishangul Gumuz; *country's estimate; ***percentage increase between 1990 and 2019*

For peer review only

Supplemental Table 2: Rate and percentage changes of episodes attributable to LRIs in 1990 and 2019 for Ethiopia and its regions, both sexes

| | **Age standardized** | | | **Children younger than 5 years** | | | **People above70** | | |
|---|---|---|---|---|---|---|---|---|---|
| **Location** | Episode per 100,000 people (95%UI),1990 | Episode per 100,000 people (95%UI),2019 | Change,% | Episode per 100,000 people (95%UI),1990 | Episode per 100,000 people (95%UI),2019 | Change,% | Episode per 100,000 people (95%UI),1990 | Episode per 100,000 people (95%UI),2019 | Change,% |
| Addis Ababa | 11373(10484.2-12197.4) | 6788.1(6285.1-7339.1) | 40 | 14666.9(11642.4-18556.9) | 4927.2(3812.5-6231.8) | 67 | 35138(29897.7-41054.2) | 30748.4(26546-35441.2) | 13 |
| Oromia | 14613.1(13525.9-15725.8) | 8659.9(8040.1-9411.6) | 41 | 22232(17397.2-28120) | 9039.4(7080.5-11277.7) | 60 | 46498.3(40057.6-54464.4) | 41564.6(36228.7-48714.4) | 11 |
| Amhara | 12826.4(11830-13882.8) | 7716.9(7162.3-8321.3) | 40 | 16487.1(13014.7-20936.6) | 8061.7(6376.4-10112.3) | 52 | 43580(37002.4-51395.2) | 35377.7(30902.9-40880.2) | 19 |
| SNNPs | 13591.9(12538.6-14646.8) | 8235.5(7670.5-8846.4) | 39 | 20106.4(15972-25079.3) | 8427.1(6799.4-10543.2) | 59 | 44628.1(37657.5-52203.1) | 36616.1(32373.5-41652) | 18 |
| Tigray | 13927.2(12858.2-15100.7) | 8663.8(8034-9382.3) | 38 | 19185.4(15207-24224.8) | 8342.1(6583.5-10430.1) | 57 | 43748.4(37005.1-51381) | 40859(35158.8-47541.2) | 7 |
| Harari | 13014.6(11941-14066.7) | 7190.5(6684.1-7718.6) | 45 | 19672.6(15471.9-25106.9) | 6821.6(5373.5-8607.3) | 66 | 33916.7(28665.1-39416.7) | 32797.4(28354-37850.7) | 4 |
| Afar | 15245.7(14050.8-16525.6) | 9350.1(8648.8-10157.4) | 39 | 21151.6(16459.1-27021.7) | 9365.5(7421.7-11714.7) | 56 | 39849(33393.8-47557.2) | 44368.7(38375.2-52580.1) | 11** |
| Somali | 11482.7(10638.1-12368.7) | 9220(8515.8-10046.3) | 20 | 16792.9(13202.4-21306.6) | 10062.7(7949.6-12802.8) | 41 | 32748.3(28159.7-39042.1) | 41031.2(35243.4-47600) | 25** |
| BG | 15628.1(14481-16809.3) | 9054.6(8394.2-9766) | 42 | 22041.1(17475.5-27805) | 10481.8(8269.5-13219) | 53 | 42911.6(36334.5-51032.5) | 37592.7(32643.9-44591.2) | 13 |
| Dire Dawa | 12806.6(11807.9-13871.4) | 7148.8(6634.6-7750.7) | 44 | 19968.2(15734.3-25484.7) | 7191.5(5637.6-9152.1) | 64 | 38333(32779.4-44607) | 32033.6(27847.4-37101.4) | 17 |
| Gambella | 13623.1(12637.4-14572.4) | 7575.8(7002.3-8128.2) | 44 | 21419.8(16947.2-27343.2) | 6791.1(5370.4-8460.1) | 69 | 40197.8(33751.8-46803.7) | 32657.1(28093.9-38087.2) | 19 |
| Ethiopia* | 13619.7(12640.8-14588.6) | 8313.7(7757.6-8918) | 39 | 19486.4(15434.9-24399.3) | 8685.3(6895.1-10789.8) | 56 | 44092.2(38166.5-50393.4) | 38394.4(33896.9-44348.5) | 13 |

SNNP: Southern Nations, Nationalities, and Peoples; BG: Benishangul Gumuz; *country's estimate*; **percentage increase between 2019 and 1990*

For peer review only

Supplemental Table 3: Number and percentage changes of death attributable to LRIs in 1990 and 2019 for Ethiopia and its regions, both sexes.

| | All age | | | Children younger than 5 | | | People above 70 years | | |
|---|---|---|---|---|---|---|---|---|---|
| **Location** | death, (95%UI),1990 | death, (95%UI),2019 | Change,% | death, (95%UI), 1990 | Death, (95%UI),2019 | Change, % | Death , (95%UI),1990 | Death, (95%UI),2019 | Change,% |
| Addis Ababa | 2159.3(1731.9-2690.7) | 918.8(767.4-1116.9) | 58 | 1353(981.9-1846.7) | 69.1(41.7-107.4) | 95 | 209.5(154.3-291.5) | 393.6(320.9-495.3) | 87** |
| Oromia | 40004.4(29432.4-51942.2) | 18206.1(15193.3-21745.4) | 55 | 30614.3(20932.5-42033.5) | 8306.2(5763.5-11611.8) | 73 | 2793.5(2005.1-3673) | 5971.6(4831.7-7093.4) | 113** |
| Amhara | 25449.3(20869.5-30724.8) | 9525.5(7530.5-11872.2) | 63 | 17780.1(13674.3-22720.1) | 3079.5(1805.3-4737.3) | 83 | 2717.2(2016.4-3535.8) | 3839.9(3022.6-4825.6) | 41** |
| SNNPs | 26044.7(20108-33017) | 9494.7(7713.5-11649) | -64 | 20390.6(14627.6-26712.9) | 4326.6(2891.3-6157.2) | 79 | 1771.7(1254.5-2417.6) | 2302.6(1867.3-2772.8) | 29** |
| Tigray | 6463.8(5272.1-7762.9) | 2551.4(2090.3-3028.6) | 61 | 4436(3373.5-5736) | 564.2(364.9-825) | 88 | 550.8(395.7-749.1) | 1209.7(978.5-1467.8) | 119** |
| Harari | 336(233.3-448.8) | 93.1(73.8-116) | 73 | 273.5(174.1-382.7) | 28.8(16.4-44.3) | 90 | 6.9(3.7-11) | 34.1(27.1-41.8) | 395** |
| Afar | 1717.5(1300.7-2243.4) | 706.2(567.7-869.6) | -59 | 1128.9(751.1-1596.7) | 284.2(182.5-426) | 75 | 66.9(43.5-102.1) | 149.3(117.3-186.6) | 123** |
| Somali | 3603.8(2620-4713.6) | 3907.5(3007.4-4959.4) | #N/A | 2811.1(1929.6-3874.8) | 2472.2(1695.2-3506.8) | 13 | 150.8(98-222.3) | 563.1(433.4-715.8) | 273** |
| BG | 1549.2(1131.1-2097) | 619(470.7-803) | 61 | 1204.8(805.6-1703.9) | 373(245.3-539.3) | 70 | 74.7(50.3-105.7) | 77(61.1-97.3) | 3** |
| Dire Dawa | 632.3(445.5-830.9) | 154.6(120.6-193.8) | 76 | 518.4(329.5-718.7) | 51(27.3-82.9) | 91 | 28.8(20.1-40) | 55(44-67.9) | 90** |
| Gambella | 475.9(328.5-635.7) | 123.2(98.2-151.9) | 75 | 398.6(253.7-555.7) | 36.4(20.7-57.2) | 91 | 28.8(18.6-41.9) | 30.9(24.6-38.1) | 7** |
| Ethiopia* | 108436.6(87669-132758.3) | 46300.7(39515.8-54642.2) | 58 | 80909.9(62491.6-103448.8) | 19591.8(14018.4-26899) | 76 | 8400.1(6490.3-10700.9) | 14627.6(12393.7-16892.8) | 74** |

SNNP: Southern Nations, Nationalities, and Peoples; BG: Benishangul Gumuz; *country's estimate; ***percentage increase between 2019 and 1990*

Supplemental Table 4: Age standardized mortality rate, Ethiopia and its regions, by sex, 2019.

| Age standardized mortality rate per 100,000 people, 2019 | | |
|---|---|---|
| **Location** | Female | Male |
| Addis Ababa | 47(35.5-62.5) | 73.6(55-98.3) |
| Oromia | 77.3(62-92.4) | 100.3(79.9-123.5) |
| Amhara | 57.9(44.1-73.7) | 92.5(70.5-122.1) |
| SNNPs | 79.2(63.5-95.2) | 118.8(94.3-146.3) |
| Tigray | 73.2(55.9-93.5) | 96.9(74.8-122.3) |
| Harari | 63(49-79.9) | 95.7(72.5-123.4) |
| Afar | 112.4(87.3-142.3) | 98.7(76.9-127.3) |
| Somali | 88.3(69-111.4) | 101.7(77.2-130.7) |
| Benishangul-Gumuz | 109.2(82.6-138.8) | 95.8(74.6-124.1) |
| Dire Dawa | 56.2(42.5-71.3) | 85.4(67.3-109.5) |
| Gambella | 47.9(34.6-60.1) | 120.2(95.6-149.5) |
| Ethiopia | 71.8(60.2-82.9) | 100.6(84-121.4) |

SNNP: Southern Nations, Nationalities, and Peoples

Supplemental Table 5: Number and percentage changes of YLL attributable to LRIs in 1990 and 2019 for Ethiopia and its regions, both sexes

| | **all age** | | | **Children younger than 5 years** | | | **People above 70** | | |
|---|---|---|---|---|---|---|---|---|---|
| | YLL, (95%UI),1990 | YLL, (95%UI),2019 | Change,% | YLL, (95%UI),1990 | YLL, (95%UI),2019 | Change,% | YLL, (95%UI),1990 | YLL, (95%UI),2019 | Change,% |
| Addis Ababa | 149543.9(115542.6-195437) | 29703.1(23833.4-36855.7) | 81 | 118961.7(86499.1-162678.7) | 6098.2(3693.1-9483.3) | 95 | 3131.8(2301.7-4374.6) | 5359.8(4334.8-6816.8) | 71** |
| Oromia | 3019031.9(2144916.2-4057496.3) | 981808.4(748459.2-1278135) | 68 | 2680397.2(1832260.4-3680767.1) | 729313.8(506226.4-1021016.9) | 73 | 44619.5(31636-59105.6) | 78166.1(62837.5-93527.9) | 75** |
| Amhara | 1813522.3(1445106.4-2246142.2) | 428277.8(309674.7-583000.2) | 77 | 1560078.3(1201305.4-1993362.8) | 270909.2(158846-416496.1) | 83 | 42578.2(31294.7-56216.9) | 50955.1(39726.1-64844.6) | 19** |
| SNNPs | 1993643.3(1489598.4-2577052.3) | 543135.8(405565.6-711397.4) | 73 | 1785675.1(1284021-2339272.5) | 380166.4(254486.8-541224.9) | 79 | 28746.2(20086.2-39789.1) | 31964.4(25673.6-38778.5) | 11** |
| Tigray | 455613.4(364406.1-569702.1) | 96429.9(74139.8-122350.5) | 79 | 389399.3(296618.1-504230.2) | 49712.6(32198.3-72764.9) | 88 | 9207.1(6568.7-12629.1) | 16086.6(12946.2-19581.8) | 74** |
| Harari | 26755.3(17866.5-36380.3) | 4273.6(2999.1-5798.4) | 85 | 23959.3(15245.4-33513.8) | 2541.6(1449.6-3887.3) | 90 | 106.7(57.6-175.1) | 455.9(361.3-558.3) | 327** |

For peer review only

| | | | | | | | | | |
|---|---|---|---|---|---|---|---|---|---|
| Afar | 124052(89386.4-166880.5) | 39507.6(29535.5-52178.5) | 69 | 99155.3(65972.9-140018.8) | 24998.1(16028.5-37426.3) | 75 | 1121.4(723.7-1722.7) | 1931.7(1521.3-2395.6) | 72** |
| Somali | 281532.2(201275.3-374880.8) | 266379.4(193669.7-357880.7) | 6 | 247138.5(169803.8-339740.3) | 216954(148995.2-307369.5) | 13 | 2256.6(1434.6-3376.4) | 7542.1(5740-9680.2) | 234** |
| BG | 119211.2(83517.3-165095.7) | 42245.6(30388.8-57591.8) | -65 | 105463.5(70756-148983.1) | 32718.1(21576.4-47307.2) | 69 | 1256.7(836.7-1780.3) | 1094.3(857.5-1389.1) | 13 |
| Dire Dawa | 50024.5(33464.5-67580.6) | 7267.7(5038-10264.7) | 86 | 45377.4(28907.2-62914.9) | 4497.4(2411.8-7298.3) | 91 | 439.2(306.6-609.5) | 742.1(586.6-922.8) | 68** |
| Gambella | 37680.8(24783.6-51516.1) | 6064.4(4389.3-7853.6) | 84 | 34899.5(22233.1-48609.4) | 3212.2(1830.4-5034.1) | 91 | 460.7(299.3-663.6) | 457.5(352.6-577.6) | 1 |
| Ethiopia* | 8070611.3(6356905.2-10091563.7) | 2445093.7(1934420.8-3119838.6) | 70 | 7090505.4(5482895.7-9059421.2) | 1721122.3(1231032.1-2362958.7) | 76 | 133924.6(102756.8-170932.9) | 194756.2(165462-225502.1) | 45** |

SNNP: Southern Nations, Nationalities, and Peoples; BG: Benishangul Gumuz; *country's estimate; ***percentage increase between 2019 and 1990*

For peer review only

Supplemental Table 6: Rate and percentage changes of YLL attributable to LRIs in 1990 and 2019 for Ethiopia and its regions, both sexes.

| | **all age** | | | **Children younger than 5 years** | | | **People above 70** | | |
|---|---|---|---|---|---|---|---|---|---|
| **Location** | YLL per 100,000 people,1990 | YLL per 100,000 people, 2019 | Change,% | YLL per 100,000 people,1990 | YLL per 100,000 people, 2019 | Change,% | YLL per 100,000 people,1990 | YLL per 100,000 people, 2019 | Change,% |
| Addis Ababa | 6362.2(5161.9-7847.9) | 1285.6(1065-1561.8) | 80 | 38824.7(28230.1-53092.4) | 6857.1(5545.8-8721.2) | 95 | 13587(9985.4-18978.3) | 6857.1(5545.8-8721.2) | 50 |
| Oromia | 11217.6(8517-14318) | 2433.8(2042.2-2879.5) | 78 | 80610.7(55103.7-110696) | 11119.7(8939.1-13305) | 87 | 18754.2(13297.1-24843) | 11119.7(8939.1-13305) | 41 |
| Amhara | 8690.4(7146.3-10453.8) | 2016.4(1551.4-2541.8) | 77 | 60663.3(46712.5-77511.5) | 9059.7(7063.2-11529.2) | 87 | 15950.4(11723.4-21059.7) | 9059.7(7063.2-11529.2) | 44 |
| SNNPs | 11633(9119.4-14477.9) | 2698.1(2243.5-3250.2) | 77 | 85114.7(61203.2-111502.1) | 11736.6(9426.7-14238.6) | 88 | 18842.6(13166.1-26081) | 11736.6(9426.7-14238.6) | 38 |
| Tigray | 10346.4(8522.9-12340.3) | 1977.1(1593.9-2395) | 81 | 68509.3(52185.7-88712.2) | 10486.8(8439.5-12765.3) | 92 | 19502.8(13914.1-26751.2) | 10486.8(8439.5-12765.3) | 47 |
| Harari | 12826.5(9336.5-16985.4) | 2060.4(1595.6-2623.1) | 84 | 100421.7(63898.7-140467.8) | 9260.4(7338.7-11340.3) | 93 | 11782.6(6360.4-19341.1) | 9260.4(7338.7-11340.3) | 22 |
| Afar | 10993.5(8520.8-14193.6) | 2824.6(2323-3414) | 74 | 63570.5(42296.6-89769) | 11867.1(9346-14716.9) | 86 | 16925.5(10923.7-26001.7) | 11867.1(9346-14716.9) | 30 |
| Somali | 6286.9(4860.5-7970) | 3236.4(2537.4-4006) | 49 | 40088.1(27543.7-55109) | 10246(7797.8-13150.6) | 57 | 11953.6(7599.2-17885.5) | 10246(7797.8-13150.6) | 15 |
| BG | 14965.1(11197.4-19998.6) | 3571.1(2772.4-4510.7) | 76 | 110847.2(74368-156588.4) | 9541.9(7476.9-12112.3) | 83 | 16397.3(10917-23228.5) | 9541.9(7476.9-12112.3) | 42 |

| | | | | | | | | | |
|---|---|---|---|---|---|---|---|---|---|
| Dire Dawa | 11749.7(8605.4-15249.5) | 1832.8(1407.3-2363.1) | 84 | 94905.3(60458.5-131584.5) | 8371(6616.3-10408.2) | 93 | 13207.9(9219.6-18328.2) | 8371(6616.3-10408.2) | 37 |
| Gambella | 13175.8(9291-17411) | 1937.4(1567.6-2344.6) | 85 | 110783.8(70576-154304.1) | 10030.9(7730.2-12664.9) | 96 | 11811.4(7673.5-17012.5) | 10030.9(7730.2-12664.9) | 16 |
| Ethiopia* | 10189.1(8347.5-12201.8) | 2404.5(2059.4-2833.3) | 76 | 72055.4(55718.5-92064) | 10309.9(8759.2-11937.6) | 86 | 17415.9(13362.7-22228.5) | 10309.9(8759.2-11937.6) | 41 |

SNNP: Southern Nations, Nationalities, and Peoples; BG: Benishangul Gumuz; *country's estimate*; **percentage increase between 2019 and 1990*

*This table indicate the rates of the years of life lost due to lower respiratory infection per 100,000 population for the year 1990 and 2019 and the percentage changes between the two years for three age categories ( for all age groups, children younger than 5 years and people above 70 years).*

For peer review only

## Authors contributions

**Providing data or critical feedback on data sources**

Semagn Mekonnen Abate, Mesafint Molla Adane, Addis Aklilu, Dejene Tsegaye Alem, Mulusew A Asemahagn, Hunegnaw Abebe, Melaku Ashagrie Belete, Tekleberhan Hailemariam, Tezera Moshago Berheto, Belay Boda Abule Bodicha, Daniel Baza Gargamo, Alemayehu Hailu, Awoke Misganaw, Mohsen Naghavi, Negussie Boti Sidamo, Yonatan Solomon, Shambel Wedajo, Melat Weldemariam, Amanuel Yigezu, Fentabil Getnet, and Yazachew Yismaw.

**Developing methods or computational machinery**

Semagn Mekonnen Abate, Tezera Moshago Berheto, Alemayehu Hailu, Mohsen Naghavi, Negussie Boti Sidamo, and Amanuel Yigezu.

**Providing critical feedback on methods or results**

Semagn Mekonnen Abate, Mesafint Molla Adane, Gizachew Taddesse Akalu, Addis Aklilu, Dejene Tsegaye Alem, Zeleke Gebru, Mulusew Andualem Asemahagn, Daniel Atlaw, Tewachew Awoke, Hunegnaw Abebe, Melaku Ashagrie Belete, Tekleberhan Hailemariam, Tezera Moshago Berheto, Alemeshet Yirga, Setognal Birara Aychiluhm, Belay Boda Abule Bodicha, Chuchu Churko, Feleke Mekonnen Demeke, Abebaw Alemayehu Desta, Lankamo Ena, Tahir Eyayu, Zinabu Fentaw, Daniel Baza Gargamo, Mesfin Damtew Gebrehiwot, Mathewos Alemu Gebremichael, Melaku Getachew, Ababi Zergaw, Alemayehu Hailu, Getahun Molla, Awoke Misganaw, Mohsen Naghavi, Biniyam Sahiledengle, Bereket Beyene, Migbar Sibhat, Negussie Boti Sidamo, Damtew Damtew Solomon, Yonatan Solomon, Birhanu Wagaye, Shambel Wedajo, Melat Weldemariam, Amanuel Yigezu, Fentabil Getnet, and Yazachew Yismaw.

**Drafting the work or revising is critically for important intellectual content**

Semagn Mekonnen Abate, Gizachew Taddesse Akalu, Mulusew A Asemahagn, Daniel Atlaw, Niguss Cherie Bekele, Melaku Ashagrie Belete, Tezera Moshago Berheto, Setognal Birara Aychiluhm, Belay Boda Abule Bodicha, Chuchu Churko, Tahir Eyayu, Zinabu Fentaw, Daniel Baza Gargamo, Melaku Getachew, Ababi Zergaw, Firehiwot Abebe Gobena, Muluken Argaw Haile, Alemayehu Hailu, Solomon Tessema Memirie, Awoke Misganaw, Mohsen Naghavi, Biniyam Sahiledengle, Bereket Beyene, Negussie Boti Sidamo, Yonatan Solomon, Dereje Mengistu Tolosa, Birhanu Wagaye, Ally Walker, Amanuel Yigezu, and Fentabil Getnet.

**Managing the estimation or publications process**

Semagn Mekonnen Abate, Awoke Misganaw, Mohsen Naghavi, Negussie Boti Sidamo, and Amanuel Yigezu.

For peer review only

STROBE Statement—checklist of items that should be included in reports of observational studies

| | Item No. | Recommendation | Page No. (Line No.) |
|---|---|---|---|
| **Title and abstract** | 1 | (*a*) Indicate the study's design with a commonly used term in the title or the abstract | 1 (1-2) |
| | | (*b*) Provide in the abstract an informative and balanced summary of what was done and what was found | 4 and 5 (73-89) |
| **Introduction** | | | |
| Background/rationale | 2 | Explain the scientific background and rationale for the investigation being reported | 7 (140-147) |
| Objectives | 3 | State specific objectives, including any prespecified hypotheses | 7 ( 148-153) |
| **Methods** | | | |
| Study design | 4 | Present key elements of study design early in the paper | 10 (194-214) |
| Setting | 5 | Describe the setting, locations, and relevant dates, including periods of recruitment, exposure, follow-up, and data collection | 8 and 9 (175-192) |
| Participants | 6 | (*a*) *Cohort study*—Give the eligibility criteria, and the sources and methods of selection of participants. Describe methods of follow-up<br>*Case-control study*—Give the eligibility criteria, and the sources and methods of case ascertainment and control selection. Give the rationale for the choice of cases and controls<br>*Cross-sectional study*—Give the eligibility criteria, and the sources and methods of selection of participants | 8 and 9 (175-192) |
| | | (*b*) *Cohort study*—For matched studies, give matching criteria and number of exposed and unexposed<br>*Case-control study*—For matched studies, give matching criteria and the number of controls per case | |
| Variables | 7 | Clearly define all outcomes, exposures, predictors, potential confounders, and effect modifiers. Give diagnostic criteria, if applicable | 9 and 10 (194-214) |
| Data sources/ measurement | 8* | For each variable of interest, give sources of data and details of methods of assessment (measurement). Describe comparability of assessment methods if there is more than one group | 8 and 9 (175-192) |
| Bias | 9 | Describe any efforts to address potential sources of bias | 10 (211-214) |
| Study size | 10 | Explain how the study size was arrived at | 8 (175-184) |

Continued on next page


For peer review only - http://bmjopen.bmj.com/site/about/guidelines.xhtml

| | | | | |
|---|---|---|---|---|
| Quantitative variables | 11 | Explain how quantitative variables were handled in the analyses. If applicable, describe which groupings were chosen and why | 9 (200-206) | |
| Statistical methods | 12 | (*a*) Describe all statistical methods, including those used to control for confounding | 9 (200-206) | |
| | | (*b*) Describe any methods used to examine subgroups and interactions | 10 (211-214) | |
| | | (*c*) Explain how missing data were addressed | 9 and 10 (194-214) | |
| | | (*d*) *Cohort study*—If applicable, explain how loss to follow-up was addressed<br>*Case-control study*—If applicable, explain how matching of cases and controls was addressed<br>*Cross-sectional study*—If applicable, describe analytical methods taking account of sampling strategy | | |
| | | (*e*) Describe any sensitivity analyses | 10 (211-214) | |
| **Results** | | | | |
| Participants | 13* | (a) Report numbers of individuals at each stage of study—eg numbers potentially eligible, examined for eligibility, confirmed eligible, included in the study, completing follow-up, and analysed | 10 (216-221) | |
| | | (b) Give reasons for non-participation at each stage | | This is a population level study |
| | | (c) Consider use of a flow diagram | | |
| Descriptive data | 14* | (a) Give characteristics of study participants (eg demographic, clinical, social) and information on exposures and potential confounders | 7 and 8 (156-168) | Described in the method section |
| | | (b) Indicate number of participants with missing data for each variable of interest | | This is a population level study |
| | | (c) *Cohort study*—Summarise follow-up time (eg, average and total amount) | 12-19 (255-361 | |
| Outcome data | 15* | *Cohort study*—Report numbers of outcome events or summary measures over time | | |
| | | *Case-control study*—Report numbers in each exposure category, or summary measures of exposure | | |
| | | *Cross-sectional study*—Report numbers of outcome events or summary measures | 12-19 (255-361 | |
| Main results | 16 | (*a*) Give unadjusted estimates and, if applicable, confounder-adjusted estimates and their precision (eg, 95% confidence interval). Make clear which confounders were adjusted for and why they were included | 12-19 (255-361 | |
| | | (*b*) Report category boundaries when continuous variables were categorized | 12-19 (255-361 | |
| | | (*c*) If relevant, consider translating estimates of relative risk into absolute risk for a meaningful time | | |



For peer review only - http://bmjopen.bmj.com/site/about/guidelines.xhtml

| | |
|---|---|
| | period |

Continued on next page


For peer review only - http://bmjopen.bmj.com/site/about/guidelines.xhtml

| | | | |
|---|---|---|---|
| Other analyses | 17 | Report other analyses done—eg analyses of subgroups and interactions, and sensitivity analyses | 12-19 (255-361 |
| **Discussion** | | | |
| Key results | 18 | Summarise key results with reference to study objectives | 19 (363-371) |
| Limitations | 19 | Discuss limitations of the study, taking into account sources of potential bias or imprecision. Discuss both direction and magnitude of any potential bias | 23 and 24 (461-471) |
| Interpretation | 20 | Give a cautious overall interpretation of results considering objectives, limitations, multiplicity of analyses, results from similar studies, and other relevant evidence | 19-23 (373-460) |
| Generalisability | 21 | Discuss the generalisability (external validity) of the study results | 24 (475-486) |
| **Other information** | | | |
| Funding | 22 | Give the source of funding and the role of the funders for the present study and, if applicable, for the original study on which the present article is based | 25 (506-508) |

*Give information separately for cases and controls in case-control studies and, if applicable, for exposed and unexposed groups in cohort and cross-sectional studies.

**Note:** An Explanation and Elaboration article discusses each checklist item and gives methodological background and published examples of transparent reporting. The STROBE checklist is best used in conjunction with this article (freely available on the Web sites of PLoS Medicine at http://www.plosmedicine.org/, Annals of Internal Medicine at http://www.annals.org/, and Epidemiology at http://www.epidem.com/). Information on the STROBE Initiative is available at www.strobe-statement.org.

For peer review only
